# Alterations in characteristics of plastic ingestion and decreasing body condition in beachcast fledgling short-tailed shearwaters (*Ardenna tenuirostris*) at Phillip Island, Australia

**DOI:** 10.1007/s11356-025-36643-6

**Published:** 2025-06-19

**Authors:** Jacinta Patricia Colvin, Peter Dann, Jasmin Hufschmid, Jeff Shimeta, Dayanthi Nugegoda

**Affiliations:** 1 School of Science, RMIT University, Melbourne, VIC Australia; 2 Research Department, Phillip Island Nature Parks, Cowes, VIC Australia; 3 Melbourne Veterinary School, Faculty of Science, University of Melbourne, Werribee, VIC Australia

**Keywords:** Procellariiformes, Seabird, Plastic effects, Microplastics, Pathology, Marine plastic debris, Plastic ingestion, Avian health

## Abstract

Plastic pollution in the marine environment is a major concern for short-tailed shearwaters (*Ardenna tenuirostris*) through high levels of plastic ingestion. Using analysis of gastrointestinal tract contents, rates of plastic ingestion were found to remain relatively high in beachcast fledglings sampled at Phillip Island, Victoria, with 87.5% and 94.7% of birds examined in 2021 and 2022 respectively containing plastic. The two years were the extreme low and high for plastic mass and number of pieces compared to previous beachcast surveys at this site. Pooling 2021 and 2022, an index of body condition for the dead birds was lower for larger plastic loads by mass, but not for the number of pieces. We believe this indicates a link between decreased fledgling viability and retained plastic mass. Changes in proventriculus plastic had the greatest impact on total plastic loads. Using additional data from 2018, a longer timeline was established for plastic loads and intestinal fat scores in these birds, revealing that the mean and maximal dimensions of particles, as well as the proportion of birds ingesting large pieces (> 10.0 mm), increased between 2018 and 2022. Plastic was mostly light-coloured particles, although there was a shift towards darker hues since 2018. Proportions of industrial pellets declined while other plastics rose. Significant necropsy findings (both plastic and non-plastic related) are reported as an overview of pathological changes occurring in these birds. A decline in body condition since 2018 and the increasing size of retained plastics indicate a heightened risk to bird health.

**Supplementary Information:**

The online version contains supplementary material available at 10.1007/s11356-025-36643-6.

## Introduction

Plastic pollution is a known threat to marine biota and a mounting global concern given its generally poor biodegradability combined with increasing production and use (Derraik 2002). Ingestion of plastic by birds has the potential to result in illness or death due to mechanical damage to the gastrointestinal tract (e.g. inflammation, ulceration, obstructions and perforations) (Pierce et al. 2004; Roman et al. 2019a), organ damage from microplastics (Rivers-Auty et al. 2023), and reduced body condition due to ingestion of materials without nutritional value, which may also reduce the capacity of the stomach to contain food before satiation occurs (Carey 2011; Ryan 1988). Plastics may contain or absorb toxicants known to be harmful, including phthalate plasticizers and hydrophobic chemicals such as polychlorinated biphenyls (PCBs) and polybrominated diphenyl ethers (PBDEs) (Hirai et al. 2011; Tanaka et al. 2013; Teuten et al. 2009). Possible links between plastic ingestion and certain persistent organic pollutant (POPs) and metals have been noted in seabirds. This includes PCBs and PBDEs in short-tailed shearwaters (*Ardenna tenuirostris*) (Tanaka et al. 2013; Yamashita et al. 2011), PCBs in great shearwaters (*Ardenna gravis*) (Ryan et al. 1988), PBDEs in northern fulmars (*Fulmarus glacialis*) (Neumann et al 2021), and chromium and silver in flesh-footed shearwaters (*Puffinus carneipes*) (Lavers et al. 2014). These findings suggest that contaminants may potentially leach from plastic when ingested and be absorbed by fauna.

**Fig. 1:**
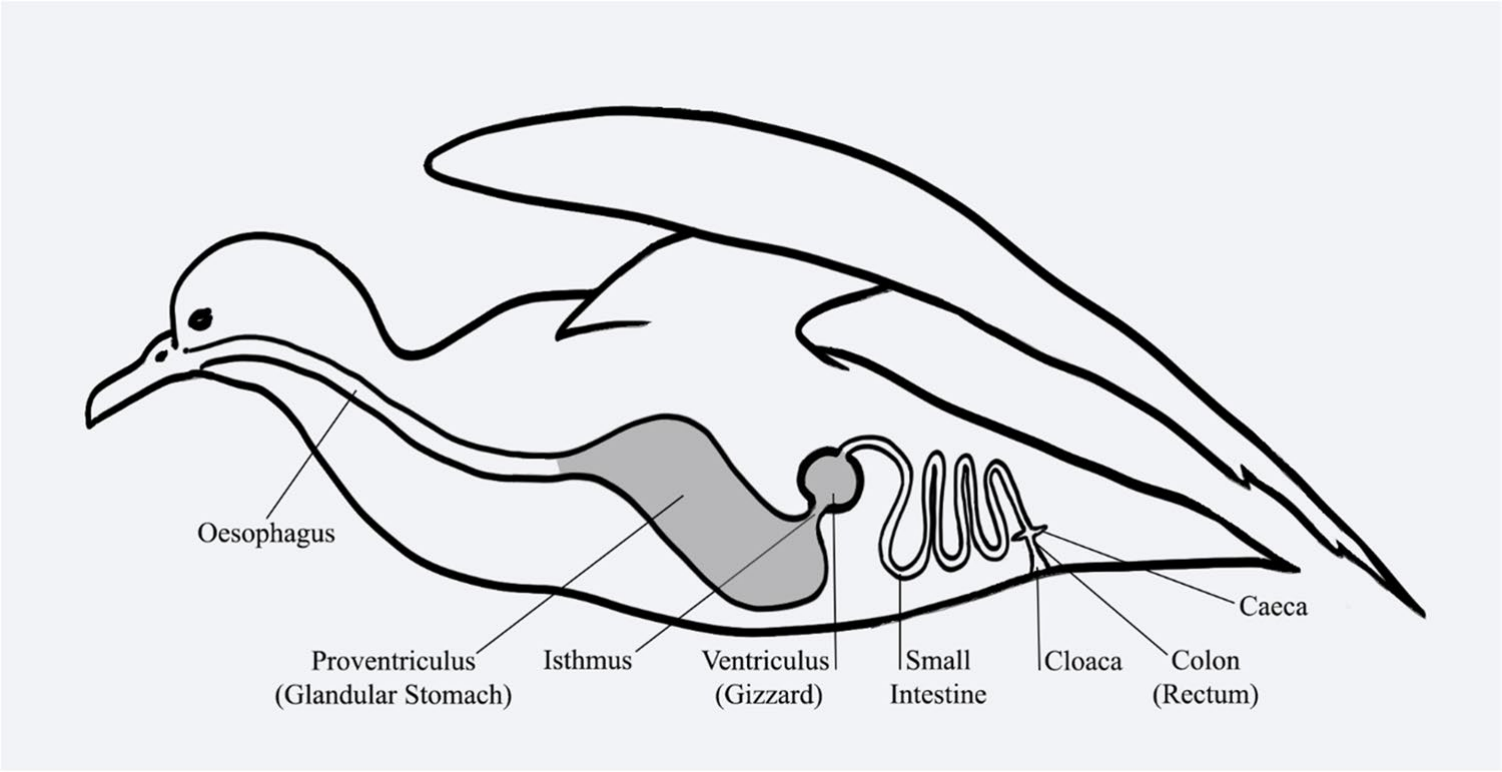
A simplified and stylised diagram of the gastrointestinal tract of a short-tailed shearwater. Plastic is frequently retained in the large, glandular proventriculus and thicker-walled, muscular ventriculus (shaded in grey)

The ingestion of plastics may thus have both direct and indirect health impacts in seabirds, although the former tend to be more easily apparent. Gastrointestinal obstructions, perforations, and ulcerations have been known to occur in seabirds as a result of plastic ingestion (Pierce et al. 2004; Roman et al. 2019a). Procellariiforms have an isthmus juncture between the proventriculus (the large, thin walled, glandular stomach of seabirds) and ventriculus (gizzard) (Fig. 1), a feature which may put them at increased risk of foreign body obstructions when items may become lodged there and are not easily regurgitated (Roman et al. 2019a). Roman et al. (2019a) found that in procellariiform seabirds, obstructions in the gastrointestinal tract leading to death were most commonly located in the ventriculus (the muscular stomach where food is mechanically broken down) or junctures leading in and out of this section of the tract. Not only may the size and composition of the plastic potentially affect its transit time through the gastrointestinal tract (Provencher et al. 2018), but its flexibility may also affect the likelihood of causing mortalities due to obstructions. Although softer plastics, rubbers, foams, and balloon fragments made up only 5.4% of the items recovered from a sample of procellariiform birds, they accounted for 42% of mortalities thought to have been caused by debris ingestion (Roman et al. 2019a). Similarly, all plastics of at least 0.5 mm in size at their greatest dimension recovered from short-tailed shearwaters (*Ardenna tenuirostris*) at Phillip Island in a previous study were located in the proventriculus and ventriculus (Colvin et al. 2020). The location within the gastrointestinal tract of the retained plastics may also influence the type of damage caused. For example, it seems likely that if not regurgitated from the proventriculus, the plastic will be retained in the proximal gastrointestinal tract, potentially allowing a build-up of items in this location until it can be broken into fragments small enough to pass into the small intestine (Provencher et al. 2018). Chicks have a reduced ability to regurgitate, which may further decrease their ability to rid themselves of ingested plastic (Acampora et al. 2014; van Franeker 2004). Therefore, the amount, length of time, and location where debris may be retained could be affected by factors including the bird’s life stage and morphology and the plastic’s size and shape (Acampora et al. 2014; Colvin et al. 2020; Provencher et al. 2018). Subsequently, somewhat unsurprisingly, studies exploring the impacts of plastic ingestion in seabirds have reported differing outcomes. Some studies have reported deleterious effects, such as a negative correlation between body mass and the number of plastic particles noted in shearwaters by Vlietstra and Parga (2002). However, other studies have failed to find significant correlations between plastic loads and body condition (Cousin et al. 2015).

Many seabird species belonging to the Order Procellariiformes (e.g. shearwaters, petrels and albatrosses) have high rates of plastic ingestion (Savoca et al. 2016). This is particularly true for those (such as shearwaters) who hunt by pursuit diving and take advantage of gyres and upwellings where debris is more likely to accumulate (Acampora et al. 2014). Additionally, some procellariiform species appear to utilise dimethyl sulfide (DMS) to locate prey, which likely causes plastic to be mistaken for food items when it becomes biofouled by biota that produce this chemical (Savoca et al. 2016). The short-tailed shearwater (*Ardenna tenuirostris*) is currently classified as ‘least concern’ by the IUCN Redlist, although its numbers are thought to be in decline (BirdLife International 2018). Recent mortality events and disturbances in migration patterns have been observed in some populations (Kuletz et al. 2020; Readfearn 2019; Van Hemert et al. 2021). The abundance of this species and high rates of plastic ingestion make them a suitable study species for monitoring marine plastic from a wildlife health perspective (Skira 1990). Plastic ingestion is commonly noted in the species, with Carey (2011) finding that 100% of the surveyed beachcast fledglings in 2010 on Phillip Island contained plastic within their gastrointestinal tract. Other research showing high instances of plastic ingestion in short-tailed shearwaters includes Cousin et al. (2015), who noted plastic in 96% of pre-fledgling chicks in Tasmania, Australia, during 2012; plastic in 89.5% of harvested fledglings examined during 2017 on Great Dog Island, Australia, by Puskic et al. (2020); and 93.5% of deceased birds necropsied during a 2019 mass mortality event were found to contain plastic in an Alaskan study by Shugart et al. (2023).

In addition to providing important information on health impacts, the surveillance of gastrointestinal contents of seabirds prone to high rates of plastic ingestion can be useful for the identification of changes in the amount and characteristics of plastic in the ocean (Lavers et al. 2018; Ryan et al. 2009). Alternative methods of assessing plastics in the marine environment include ocean trawls and beach surveys, which have their own advantages and disadvantages, such as cost and a host of environmental factors that can affect the recovery of plastics (Ryan et al. 2009). Opportunistic sampling can be employed by utilising deceased beachcast or harvested birds, those accidentally killed during fishing activities or road fatalities, or via inspection of regurgitated pellets from predators that feed on these seabirds (Cousin et al. 2015; Rodríguez et al. 2018; Ryan et al. 2009). Alternatively, planned sampling can be employed by flushing and collecting the stomach contents (Lavers et al. 2018), or collection of birds euthanised for the purpose of that study (Pollet et al. 2024). Monitoring over multiple years allows assessment of trends in the type of plastics being ingested and retained, and of the health of birds being surveyed. At Phillip Island, Victoria, beachcast birds are reliably found at the time of their departure during April–May each year (Carey 2011; Colvin et al. 2020; Rodríguez et al. 2018).

Phillip Island supports a significant population of short-tailed shearwaters, with approximately 450,000 nests present in 2007 (BirdLife International 2024). It has been a site of prior monitoring of plastic ingestion for this species, with previous publications identifying a high percentage of fledglings with plastic in their gastrointestinal tracts (98–100% of beachcast birds in years sampled between 2010 and 2018, and 97% of road-killed fledglings collected over 2015–2016) (Carey 2011; Colvin et al. 2020; Rodríguez et al. 2018). This study aimed primarily to investigate the impacts of plastic ingestion on the health of short-tailed shearwater fledglings. Data were obtained from beachcast birds on Phillip Island between 2018 to 2022. Associations between plastic ingestion and body condition based on amount and size of plastics ingested and the retained location within the gastrointestinal tract were explored, and any changes in the amount or characteristics of plastics recovered over time were analysed. Additionally, potential impacts of retained plastic on the amount of food present within the proventriculus were investigated to determine if greater amounts of plastic decreased the amount of actual food present. A survey of both plastic and non-plastic related pathological lesions based on gross postmortem examination was conducted to assess the general state of health of shearwaters becoming beachcast at this location.

## Methods

Forty-three recently deceased short-tailed shearwater fledglings were collected from the beaches on Phillip Island, Victoria, Australia (coordinates: 38° 32′ 27.3″ S 145° 20′ 09.8″ E) each year in 2021 and 2022 during the peak period of their departure in April–May (May 1–8 in 2022, April 22–28 in 2021). In addition, data on gastrointestinal contents and intestinal fat scores previously collected on 52 birds using the same method and location between April 30 and May 2 in 2018 (Colvin et al. 2020) was included in some of the statistical analysis to investigate more long-term trends. All birds collected had failed to depart the local area for their migration and were unlikely to have eaten since last fed by their parents 2–3 weeks earlier; adult birds generally leave the breeding grounds by mid-April (Department of Natural Resources and Environment Tasmania 2019; Marchant and Higgins 1990).

Necropsies and measurements (body condition and plastic characteristics) on all birds (2018–2022) were performed by the same veterinarian to reduce potential variability on scoring opinions that might occur between different people. Full necropsies were performed as part of the present study on birds collected in 2021 and 2022. Each of these birds was inspected for signs of injury, pathological changes, scavenging and presence of anthropogenic debris within the gastrointestinal tract. A total of seventy-eight necropsies were performed on birds with complete and unperforated gastrointestinal tracts (*n* = 40 and *n* = 38 for 2021 and 2022, respectively. Data from an additional 52 intact gastrointestinal tracts was also included from 2018) and were thus included for further examination of total retained plastic loads. As part of the postmortem examinations in 2021 and 2022, the appearance of the gonads, presence of the bursa of Fabricius, and maturity of feathers (presence of any down, and flight feather maturity) were inspected. Abnormalities were recorded, with ante or perimortem predation rather than scavenging noted when showing signs of bruising or haemorrhage (Dileka et al. 2022; Sorg 2019). Findings are based on gross examination only; no histopathology was performed. A ‘Body Condition Index’ (BCI), scored from 0 to 9, was obtained by adding the three separate condition scores of intestinal fat, pectoral muscle, and subcutaneous fat (each scored from 0 to 3, where 0 is very low and 3 indicates high levels) following van Franeker (2004) where the level of scavenging allowed for an accurate assessment of all three condition scores. Van Franeker (2004) suggested that a BCI of 0–1 indicates a bird is likely to be mortally emaciated, 2–3 is critically underweight, 4–6 indicates moderate body condition, and 7–9 indicates birds have a good body condition. Whole bird mass was not used as a metric of body condition due to partial scavenging of some birds which would have prevented an accurate whole-body weight from being obtained. In contrast, for the samples collected in 2018, body condition could only be assessed based on intestinal fat; therefore, long term (2018–2022) comparisons for body condition could only be made based on intestinal fat scores.

The gastrointestinal contents from the proventriculus and ventriculus were weighed separately. The contents from the proventriculus, ventriculus, and intestines were then assessed while viewing with a dissection microscope in petri dishes with milli-Q water used as required to rinse items so they could be better visualised and separate the likely plastic from the remaining ingesta following Rodriguez et al. (2018). Collected plastic was washed in milli-Q water and air dried until at constant weight. The mass was measured by a digital balance to the nearest 0.1 mg (Carey 2011). The maximum dimension of each piece of plastic to the nearest 0.01 mm was measured using Leica Application Suite (Version 4.9.0) from images obtained from the connected Leica MZ9.5 microscope. Only plastics with a minimum size of 0.5 mm or greater in at least one dimension were included in the research for consistency with the previously published study of 2018 birds (Colvin et al. 2020). Any objects of uncertain type were checked for deformation with a needle heated in flame until red hot and applied to the surface of the object (Mossman 2008). If any doubt remained as to the identity of the particle, it was earmarked to be checked with attenuated total reflection Fourier transform infrared spectroscopy (ATR-FTIR) to confirm the nature of the material. Although not all plastic in the study was confirmed with polymer identification technology, the identity of plastic obtained from 31 of the birds collected in 2018 (163 particles) was confirmed using ATR-FTIR (Colvin et al. 2020) and assisted with ensuring the researcher was selecting for plastic accurately (no misidentification occurred within the tested sample). Analysis was performed using a PerkinElmer Frontier FT-IR/FIR Spectrometer, with a PIKE GladiATR technologies attachment. Samples were prepared for analysis by cleaning with 70% ethanol and air drying. Where the dimensions of the plastic allowed, the sample was incised with a scalpel blade to present a flat, ‘clean’ surface in contact with the crystal (Jung et al. 2018). Analysis of the FTIR spectra was performed using Spectrum software (PerkinElmer Inc. 2015).

Methodology for classifying plastics by shape, colour, and origin was selected to allow comparisons where possible to data collected in 2018 (Colvin et al. 2020) and 2010 (Carey 2011). Collected plastics were categorised by three origin types: user (e.g. fragmented pieces of household items), industrial (e.g. pellets and beads used in cargo packaging and moulding), and other plastics (e.g. synthetic fibres, strings, rubbers and plastic sheets) using methods in Vlietstra and Parga (2002) and Ogi (1990). Particles were also sorted by appearance into foams, fibres, fragments, beads (small spherical objects), and pellets (small, solid, cylindrical objects) following Lusher et al. (2017). Plastics were additionally categorised into highly flexible (such as fibres and plastic films) and rigid plastics to determine if flexibility might be a factor in impacts on health. If the dimensions of the plastic allowed, it was scraped using a scalpel to determine the original colour (Acampora et al. 2014) and then classed following Vlietstra and Parga (2002) into light (white, yellow, and yellow–brown), medium (blue, brown, green and red), and dark (dark blue, dark green, dark red, and grey-black) shades.

Both unpublished and published (Colvin et al. 2020) values used in the analysis of the 2018 dataset within this paper are stated for ease of reference. Statistical analysis was performed using Excel (Microsoft 2022), SPSS Statistics (IBM 2021), and Minitab (Minitab LLC 2021). As assumptions for parametric tests could not be met for the majority of datasets, appropriate non-parametric tests were utilised including: chi-squared (*χ*^2^) for data with categorical/ordinal categories (e.g. comparing proportions of plastic by colour, shape and origin between years); Fisher-Freeman-Halton Exact where some categories had expected frequencies less than five (e.g. trends in fat stores over time); Mann–Whitney *U* (*U*) to compare differences in mean ranks (e.g. comparing the mean body condition index over time, amount and size of plastic between different areas of the gastrointestinal tract, and body condition of high vs lower plastic loaded birds); and Kruskal–Wallis one-way ANOVA (*H*) for analysing > 2 years of continuous data (e.g. maximum plastic dimensions between 2018 and 2022) with a post hoc pairwise test performed if a significant result was present. Kendal’s Tau (τ_b_) was used to perform non-parametric correlations between two variables (e.g. correlations between body condition score and maximum size of retained plastic). A Levene’s test was used where it was necessary to test the null hypothesis that the samples were of the same variance (*s*^*2*^). Outliers in the upper range (those exceeding Q3 + 1.5 * Inter Quartile Range) were calculated for identifying thresholds for birds with very high amounts of plastic ingestion. The significance criterion for all tests was set at *α* = 0.05. All data are presented as means ± standard error (S.E.) unless otherwise indicated.

## Results

### Postmortem summary and body condition

Fifty-three out of eighty-six birds showed at least minor evidence of scavenging. A suspected cause of death could not be definitively determined due to damage caused by scavenging in 20 birds (including all eight birds without complete gastrointestinal tracts.) Confirmation of the precise cause of death is beyond the scope of this study, and findings are based on gross postmortem examination results alone. However, likely cause of death could be estimated in at least some of the birds based on gross pathology findings and included drowning, predation, and trauma due to unidentified causes. Twenty-one individuals contained water in their airways and were considered likely to have drowned. A further two contained pale red fluid, consistent with blood that had been diluted by water in their airways along with injuries consistent with attack by Pacific gulls (*Larus pacificus*) which were observed killing and scavenging grounded and beachcast shearwaters at this location. This suggests that both trauma and water aspiration (either before or during the attack which frequently occurred in or on the water’s edge) contributed to these birds’ deaths. Four birds not only had aspirated water but also showed evidence of trauma (including internal bleeding, bruising and/or skull fractures) of uncertain cause. Although predation might have accounted for some of the injures, other causes, such as damage suffered in the surf (e.g. being washed against rocks), are possible. Twenty-eight birds had damage consistent with attack by gulls (peck marks, beak sized puncture wounds, or were observed killing the birds in question) but had no water within their airways, making it likely that this trauma contributed to the birds’ deaths. Three birds had no aspiration of water, and showed signs of significant trauma (bruising, haemorrhage, fractures) which appeared most typical of blunt force injuries (e.g. falls from a height, washed against rocks etc.). Seven individuals were wet but displayed no evidence of trauma or aspiration of water, and therefore, the cause of death is uncertain. A single bird was small, thin, dry, and unscavenged. It likely succumbed as a result of an infected, flyblown (containing maggots) wound (see Appendix B for detailed necropsy descriptions).

**Fig. 2:**
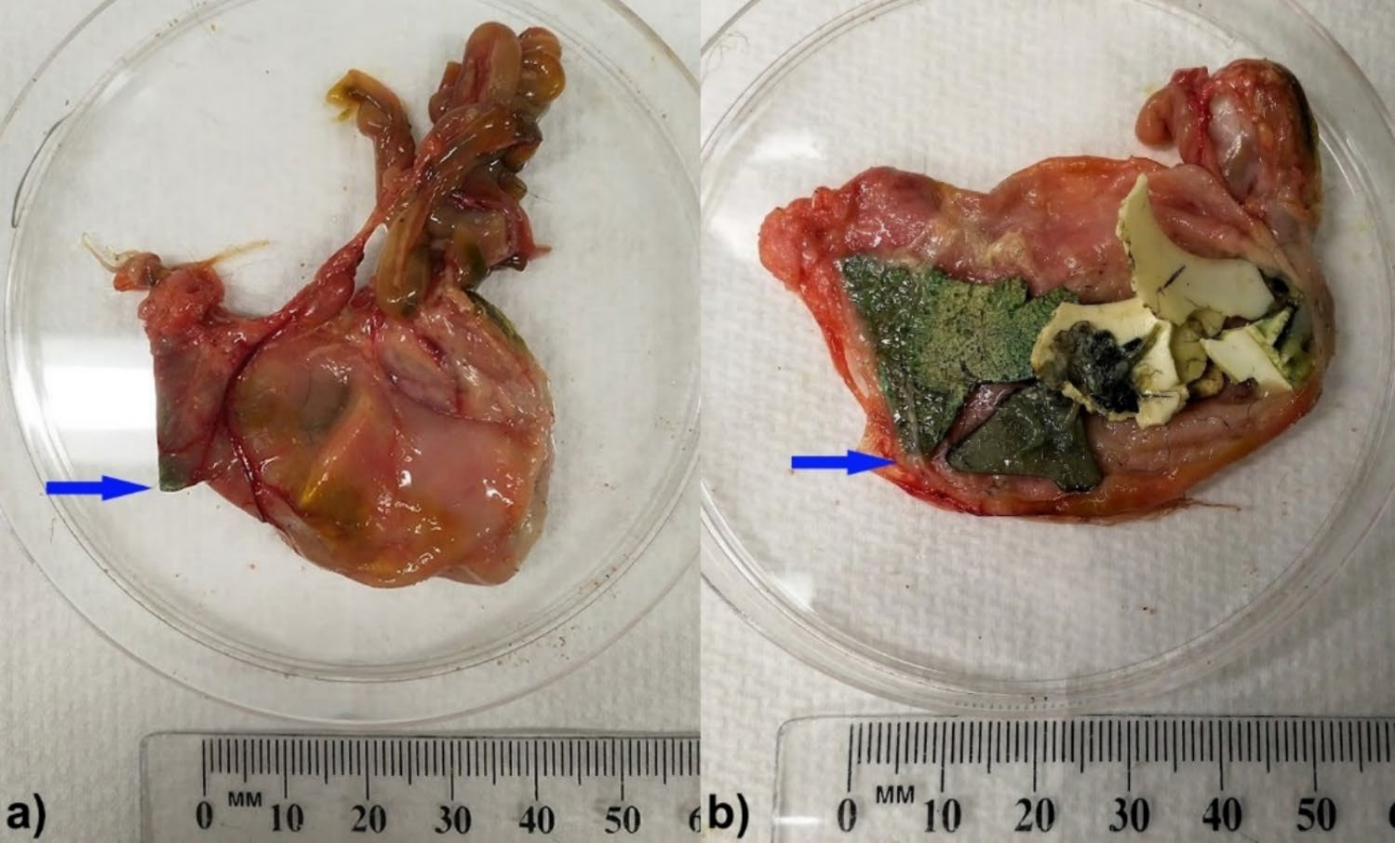
A short-tailed shearwater (*Ardenna tenuirostris*) fledgling containing multiple pieces of plastic, including a large green fragment protruding into the proventricular wall, ulcerating the mucosal surface (arrows). Adhesions on the mucosal surface to the plastic and inflammation with increased blood supply (erythema of tissues and prominent, dilated blood vessels) were present in this area. External (**a**) and dissected (**b**) views are shown

**Fig. 3:**
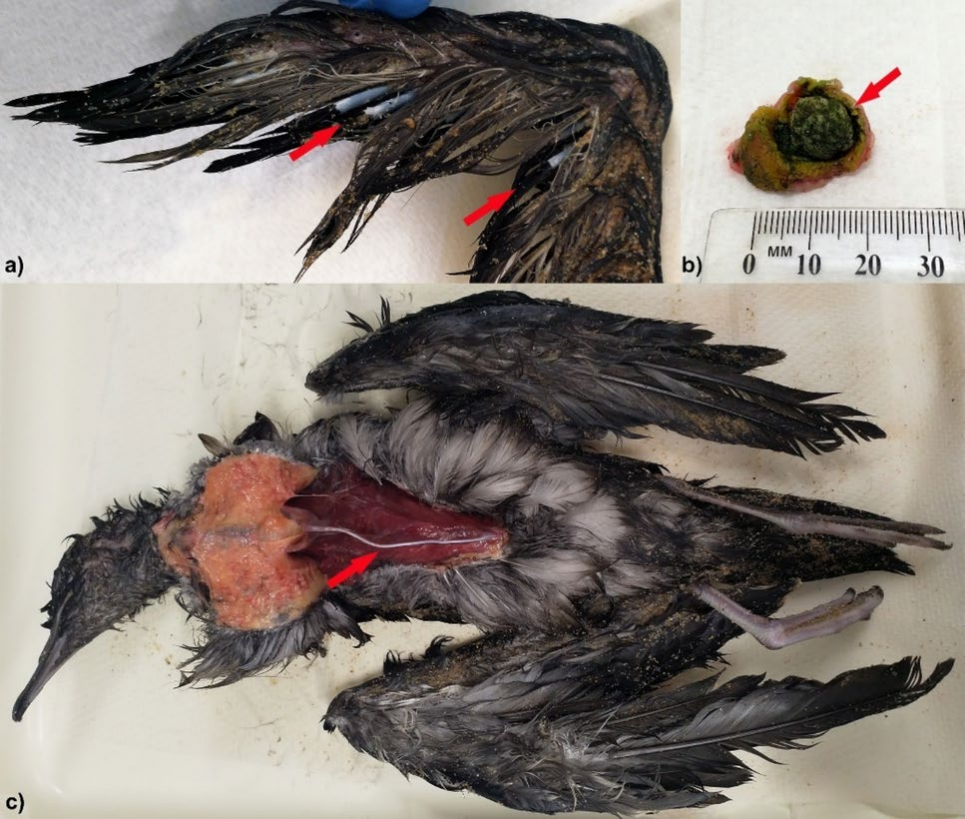
Examples of non-plastic related pathology identified in fledgling short-tailed shearwaters (*Ardenna tenuirostris*) found on Phillip Island, Victoria, including **a** abnormal primary wing feathers (arrows); **b** a piece of pumice (arrow) lodged in the ventriculus resulting in likely obstructive gastrointestinal disease; and **c** a flexible, deformed sternum (arrow) suggestive of metabolic bone disease

Additional pathological changes noted in the beachcast birds included six birds with inflammation of the proventriculus, five of which included ulceration of the mucosa. Two of these birds had a large piece of plastic protruding into the proventricular wall with ulceration and erythema (red discolouration consistent with inflammation) of the tissues. The plastics adhered to the proventriculus with fibrinous attachments. One case appeared close to perforating the wall, and prominent dilated blood vessels were present in this area (Fig. 2). All birds with proventricular ulceration/inflammation contained plastic in this location, and with one exception (BCI of 7), had a BCI of three or less indicating that they were considered critically to mortally underweight. Seven birds had obstructive disease of their gastrointestinal tract caused by foreign body ingestion, five of which obviously involved plastic. One bird had a large piece of cuttlefish lodged in the narrowed, distal section of the proventriculus close to the isthmus. A further six had obstructions noted in their ventriculus. One was caused by a piece of pumice (plastic was also present but not the major cause of the obstruction), the second was due to impacted kelp and plastic (plastic was up to 8.7 mm in largest dimension), the third obstructed due to a tightly packed mixture of plastic, rocks, and cuttlefish fragments in the ventriculus (and also contained a large number of rocks in the proventriculus), and the fourth had multiple pieces of fishing line of up to 73.8 mm in length entangling rocks and shells which would have resulted in a partial obstruction of the ventriculus. The fifth bird had seventeen pieces of plastic in its ventriculus likely causing obstructive disease as there was a very large amount of fluid in the proventriculus, but little ingesta in the intestines. The sixth bird had fifteen pieces of plastic in its ventriculus likely causing a partial obstruction, with little ingesta and areas of gas present in the intestines despite moderate amounts of ingesta still remaining in the proventriculus. Non-gastrointestinal pathology included a chronically infected, flyblown neck wound (as stated above), a poorly healed leg fracture which would have prevented standing/normal swimming and showed signs of development of a pressure sore, a bird with bones that had more flexibility than typical and which clinically resembled metabolic bone disease, and a bird with abnormal primary and secondary flight feathering on both wings (Fig. 3, Appendix B). All birds in the study had small gonads and a bursa of Fabricius indicating that they were immature animals. The presence of non-breeding immature birds older than fledglings cannot be completely ruled out; however, this subset of the population (immature birds ≤ 5 years of age) generally leave by mid-March (Marchant and Higgins 1990). Severe emaciation (BCI = 0) was recorded in 9.8% individuals in 2021 (*n* = 41), and 15.0% of birds in 2022 (*n* = 40).

**Fig. 4:**
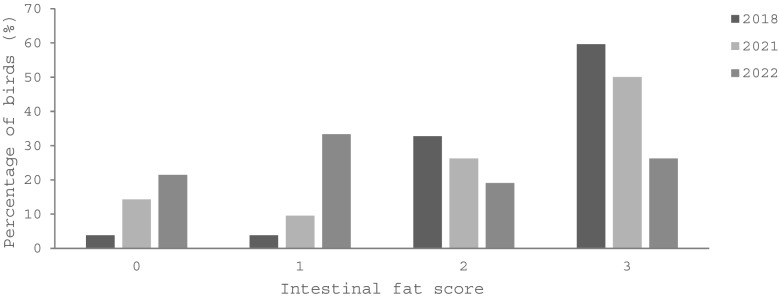
The percentage of beachcast short-tailed shearwaters (*Ardenna tenuirostris*) on Phillip Island in 2018 (*n* = 52) (Colvin et al. 2020), 2021 (*n* = 42), and 2022 (*n* = 42) (this study) categorised by intestinal fat score, as detailed by van Franeker (2004). A score of ‘0’ indicates very little adipose, while a score of ‘3’ indicates large fat deposits were present. The difference among the 3 years was significant (Fisher-Freeman-Halton Exact: 28.2, df: 6, *p* < 0.001)

When assessing body condition, a significant falling trend in intestinal fat stores was observed in the years measured between 2018 and 2022, indicating lowered body condition in beachcast birds at this location over time (Fisher-Freeman-Halton Exact: 28.2, df: 6, *p* < 0.001). Although each year the mean intestinal fat score decreased with a score of 2.5 in 2018 to 1.5 in 2022 (Table 1, Fig. 4, Appendix A-Table 8), differences between individual years were only significant between 2021 and 2022 (Fisher-Freeman-Halton Exact: 2018–2021: 4.9, df: 3,* p* = 0.2; 2021–2022: 9.8, df:3, *p* = 0.02.) When the BCI was considered, the mean decreased from 5.7 (min–max: 0–9) in 2021 to 4.1 (min–max: 0–9) in 2022 (*U* = 620.0,* p* = 0.06, *n* = 81) (Table 1, Appendix A-Table 9). Measurements for BCI (*n* = 81), intestinal fat (*n* = 84), pectoral muscle (*n* = 82), and subcutaneous fat (*n* = 85) levels were highly correlated at *p* < 0.001 (intestinal fat/subcutaneous fat *τ*_b_ = 0.9, intestinal fat/muscle τ_b_ = 0.7, intestinal fat/BCI *τ*_b_ = 0.9, subcutaneous fat/muscle *τ*_b_ = 0.8, subcutaneous fat/BCI *τ*_b_ = 0.9, muscle/BCI *τ*_b_ = 0.8.)

**Table 1:** The number of observations (*n*), median (Med), and mean body condition score measurements for intestinal fat, subcutaneous fat, pectoral muscle, and body condition index occurring in beachcast short-tailed shearwaters (*Ardenna tenuirostris*) collected on Phillip Island in 2018, 2021, and 2022

Year	Intestinal fat (0–3)			Subcutaneous fat (0–3)			Pectoral muscle (0–3)			Body condition index (0–9)		
	Mean	Med	***n***	Mean	Med	***n***	Mean	Med	***n***	Mean	Med	***n***
**2018**	2.5	3.0	52	NA*	NA*	-	NA*	NA*	-	NA*	NA*	-
**2021**	2.1	2.5	42	1.8	2.0	42	1.8	2.0	41	5.7	7.0	41
**2022**	1.5	1.0	42	1.4	1.0	43	1.3	1.0	41	4.1	3.0	40

*NA not available

### Amount and location of retained ingested anthropogenic debris

Almost all birds contained plastic in their proventriculus and/or ventriculus in 2021 (87.5%, *n* = 40) and 2022 (94.7%, *n* = 38). A single bird in 2021 contained one piece of glass which was not included in the plastic analysis and the only other anthropogenic item noted. The most common location to recover plastic was the ventriculus, with 91.4% (*n* = 35; 0–17 pieces) and 97.2% (*n* = 36; 0–17 pieces) of all birds sampled that had retained plastic within their gastrointestinal tract in 2021 and 2022, respectively. For the proventriculus, it was 34.3% (0–7 pieces) and 63.9% (0–13 pieces) in 2021 and 2022, respectively. No plastic (max dimension ≥ 0.5 mm) was found elsewhere in the gastrointestinal tract. The mean number of plastic pieces located in the ventriculus of all sampled birds in 2021 and 2022 (5.3 ± 0.5, median: 4.0, *n* = 78) was significantly larger than in the proventriculus (mean = 1.4 ± 0.3, median: 0.0, *n* = 78) (*U* = 1244.5, *p* < 0.001). However, by contrast, the mean mass of plastic present in the ventriculus (81.7 mg ± 8.1, median: 67.5, *n* = 78) was significantly smaller than that present in the proventriculus (mean = 87.4 mg ± 26.2, median: 0.0, *n* = 78) (*U* = 1820.0, *p* < 0.001).

In total, 203 pieces of plastic were recovered in 2021, giving a mean of 5.1 ± 0.7 pieces per bird (median: 4.0, 0–17 pieces) or 85.8 mg ± 11.8 (median: 66.2 mg, 0–281.1 mg) per bird. A total of 322 pieces of plastic were recovered from birds in 2022. This equates to a significantly higher mean of 8.5 ± 0.9 pieces (median: 8.5, 0–22 pieces) or 257.0 mg ± 52.7 (median: 157.2 mg, 0–1558.7 mg) (particles: *U* = 474.5, *p* = 0.004; mass: *U* = 387.0, *p* < 0.001) per bird (Table 2). When comparing data collected in 2018 (310 pieces, particles mean: 6.0 ± 0.6, median: 5.0, 0–22; mass mean: 107.3 mg ± 15.1, median: 80.8, 0–640.1 mg) with 2022, the increase in plastic ingestion remained significant (particles: *U* = 698.0, *p* = 0.02; mass: *U* = 587.5, *p* = 0.001). Nonetheless, it should be noted that there was a trend for lower plastic ingestion in 2021 compared to 2018 (2021 was a historical low) although the difference was not statistically significant (particles: *U* = 879.0, *p* = 0.2; mass: *U* = 926.0, *p* = 0.4) (Table 2).

**Table 2:** The percentage of beachcast fledgling short-tailed shearwaters (*Ardenna tenuirostris*) found to contain plastic in the gastrointestinal tract and plastic retention per bird by mass and number of pieces by their median (Med) and mean ± S.E over time at Phillip Island, Victoria, during 2018, 2021, and 2022

**Year**	**All birds sampled**						**Only birds with ingested plastic**				
	**Proportion ingesting plastic (%)**	**Pieces (number)**		**Mass (mg)**		***n***	**Pieces (number)**		**Mass (mg)**		***n***
		**Mean**	**Med**	**Mean**	**Med**		**Mean**	**Med**	**Mean**	**Med**	
2018*	98.1	6.0 ± 0.6	5.0	107.3 ± 15.1	80.8	52	6.1 ± 0.6	5.0	109.4 ± 15.3	81.3	51
**2021**	87.5	5.1 ± 0.7	4.0	85.8 ± 11.8	66.2	40	5.8 ± 0.8	5.0	98.0 ± 12.1	83.3	35
**2022**	94.7	8.5 ± 0.9	8.5	257.0 ± 52.7	157.2	38	8.9 ± 0.8	9.5	271.2 ± 54.6	171.9	36

*Calculated from the 2018 raw dataset used with Colvin et al. (2020) with additional unpublished values listed

When only birds containing plastic were considered to check if there were differences in plastic loads between the years among only those who had retained ingested debris (as the percent of birds ingesting plastic each year differed), individuals contained a mean of 5.8 pieces ± 0.8 (median: 5.0, 1–17 pieces) or 98.0 mg ± 12.1 (median: 83.3, 1.1–281.1 mg) of plastic per bird in 2021 (*n* = 35). In 2022 there was a significant rise to a mean of 8.9 pieces ± 0.8 (median: 9.5, 1–22 pieces) (*U* = 399.5, *p* = 0.008) or 271.2 mg ± 54.6 (median: 171.9, 10.7–1558.7 mg) (*U* = 312.0, *p* < 0.001) of plastic (*n* = 36) (Table 2). This rise was also significant between 2018 (particles: mean: 6.1 ± 0.6, median: 5.0, 1–22; mass: mean: 109.4 mg ± 15.3, median: 81.3, 5.9–640.1 mg, *n* = 51) and 2022 (particles: *U* = 595.0, *p* = 0.005, mass: *U* = 484.5,* p* < 0.001). However, the difference was not statistically significant between 2018 and 2021 (particles: *U* = 841.5, *p* = 0.7, mass: *U* = 888.5, *p* = 1.0) (Table 2).

Analysing only birds who had ingested at least one piece of plastic between 2018 and 2022, the year 2021 had both the lowest mean total plastic mass, and the lowest percentage of birds with plastic in the proventriculus or the ventriculus. The year 2022 was the highest for all these parameters. Between 2021 and 2022 the mean total mass of plastic increased by 173.2 mg, while the percentage of birds containing at least once piece of plastic in their proventriculus increased by 29.6%, with a 5.8% increase for the ventriculus. The actual mass of plastic retained in both the proventriculus and ventriculus also followed this pattern with 2022 having highest values, and 2021 the lowest, with an increase of 152.2 mg for the proventriculus, and 21.1 mg for the ventriculus between these 2 years. The change in the mass of plastic present in the proventriculus for the years 2018, 2021, 2022 was significant (*H* = 11.6, df: 2, *p* = 0.003, *n* = 122). The difference was not significant for the ventriculus (*H* = 1.9, df: 2, *p* = 0.4, *n* = 122) (Table 3).

**Table 3:** Location and mass (mg) of plastic within the stomachs of beachcast fledgling short-tailed shearwaters (*Ardenna tenuirostris*) who had ingested at least one piece of plastic at Phillip Island, Victoria, during 2018, 2021, and 2022. The mean mass ± S.E., median (Med), minimum–maximum values, and *n* are presented

Year	**Proventriculus**				**Ventriculus**				
	**% with plastic**	**Mean mass**	**Med**	**Min–Max**	**% with plastic**	**Mean mass**	**Med**	**Min–Max**	***n***
2018*	49.0	22.4 ± 6.0	0.0	0–199.9	98.0	87.0 ± 14.1	57.8	0–617.0	51
2021	34.3	18.9 ± 5.9	0.0	0–127.0	91.4	79.1 ± 10.5	64.8	0–229.1	35
2022	63.9	171.1 ± 53.4	34.2	0–1484.0	97.2	100.2 ± 12.6	99.8	0–339.3	36

*Calculated from the 2018 raw dataset used with Colvin et al. (2020)

### Physical characteristics of the recovered plastics

An analysis of size and colour proportions was conducted on plastic recovered from 2018, 2021, and 2022. Overall (combined 2018: *n* = 309, 2021: *n* = 203, 2022: *n* = 322), the mean maximum dimension of retained plastics was 6.1 mm. The mean maximum dimension increased over time, being 4.6 mm in 2018, 6.3 mm in 2021, and 7.5 mm in 2022, a change that was significant (*H* = 77.6, df: 2,* p* = < 0.001, *n* = 834.) A post hoc pairwise comparison found the difference in size was significant between 2018 and 2021 (*p* < 0.001) but not for 2021 to 2022 (*p* = 0.3). If fibres, string, and plastic film, which were often long but thin and flexible, were excluded from the analysis, the mean maximum dimension of only rigid plastics decreased, with a mean of 4.4 mm in 2018 (max: 10.4 mm, *n* = 304), 5.6 mm in 2021 (max: 15.8 mm, *n* = 198) and 6.8 mm in 2022 (max: 30.5 mm, *n* = 310). The change in size considering only rigid plastics between years remained significant (*H* = 74.4, df: 2,* p* < 0.001, *n* = 812.) A post hoc pairwise comparison for rigid plastics was significant only for the difference between 2018 and 2021 (*p* < 0.001) (2021–2022: *p* = 0.4) (Table 4). The percentage of birds ingesting rigid plastic larger than 10.0 mm rose over time, with 1.9% in 2018 (*n* = 52), 10.0% in 2021 (*n* = 38), and 50.0% in 2022 (*n* = 40).

**Table 4:** The maximum dimensions (mm) of plastic retained by fledgling short-tailed shearwaters (*Ardenna tenuirostris*) in 2018, 2021, 2022, and overall (combined years). There was a statistically significant difference between years when all plastic (*H* = 77.6, df: 2,* p* = < 0.001, *n* = 834) and only rigid plastics (*H* = 74.4, df: 2,* p* < 0.001, *n* = 812) were analysed

Year	2018*	2021	2022	Overall
***All plastic***				
Mean ± S.E	4.6 ± 0.1	6.3 ± 0.4	7.5 ± 0.4	6.1 ± 0.2
Median	4.3	5.4	5.5	5.0
Min–Max	0.5–27.0	0.6–80.9	1.4–73.8	0.5–80.9
*n*	309	203	322	834
***Only rigid plastic***				
Mean ± S.E	4.4 ± 0.1	5.6 ± 0.2	6.8 ± 0.3	5.6 ± 0.1
Median	4.2	5.4	5.4	4.9
Min–Max	0.5–10.4	0.6–15.8	1.4–30.5	0.5–30.5
*n*	304	198	310	812

*Calculated from the 2018 raw dataset used with Colvin et al. (2020) with additional unpublished values listed

Plastics in the proventriculus (mean: 8.4 mm, *n* = 178) tended to have significantly larger maximum dimensions compared to those in the ventriculus (mean: 5.5 mm, *n* = 656) (*U* = 36,727.0, *p* < 0.001, *n* = 834). The maximum dimension of proventriculus plastics rose significantly between 2018 and 2022, with a mean of 4.8 mm in 2018, 7.9 mm in 2021, and 11.5 mm in 2022 (*H* = 54.0, df: 2, *p* < 0.001, *n* = 178). The difference in the maximum dimension of ventriculus plastic between 2018 and 2022 was also significant, increasing from a mean of 4.5 mm in 2018 to a mean of 6.1 mm in both 2021 and 2022 (*H* = 39.9, df: 2, *p* < 0.001, *n* = 656). For both the proventriculus and ventriculus plastic, a post hoc pairwise test showed the change in size only to be significant between 2018 and 2021 (*p* < 0.001) (2021–2022: proventriculus *p* = 0.2, ventriculus *p* = 0.7) (Table 5).

**Table 5:** The maximum dimensions (mm) of plastic retained by fledgling short-tailed shearwaters (*Ardenna tenuirostris*) in their proventriculus and ventriculus during 2018, 2021, 2022, and overall (combined years). There was a statistically significant difference in size between years in both the proventriculus (*H* = 54.0, df: 2, *p* < 0.001, *n* = 178) and ventriculus (*H* = 39.9, df: 2, *p* < 0.001, *n* = 656)

Year	2018	2021	2022	Overall
***Proventriculus***				
Mean ± S.E	4.8 ± 0.3	7.9 ± 0.6	11.5 ± 0.8	8.4 ± 0.5
Median	4.5	7.1	8.3	6.4
Min–Max	0.5–9.8	4.9–17.2	2.4–37.8	0.5–37.8
*n*	67	28	83	178
***Ventriculus***				
Mean ± S.E	4.5 ± 0.1	6.1 ± 0.5	6.1 ± 0.4	5.5 ± 0.2
Median	4.2	5.3	5.0	4.7
Min–Max	1.9–27.0	0.6–80.9	1.4–73.8	0.6–80.9
*n*	242	175	239	656

**Fig. 5:**
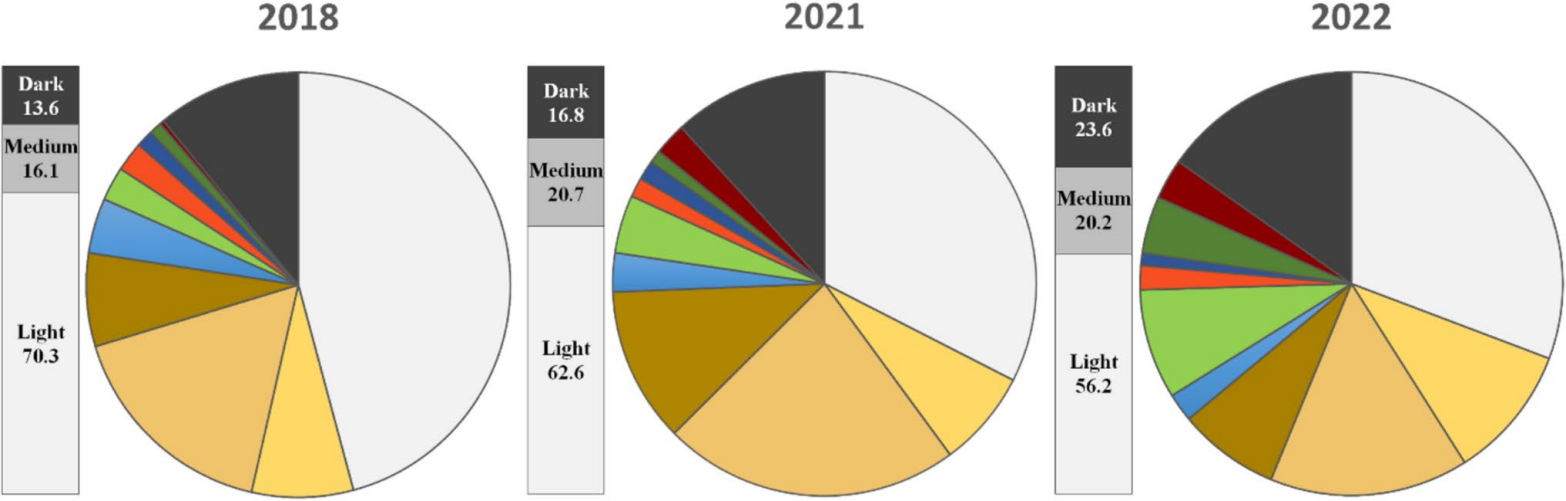
The percentage of plastic particles by colour collected from the ventriculus and proventriculus of beachcast short-tailed shearwater (*Ardenna tenuirostris*) fledglings located on Phillip Island during 2018 (*n* = 310) (Colvin et al. 2020), 2021 (*n* = 203) and 2022 (*n* = 322) (this study). Light shades included white, yellow, and yellow–brown; medium shades were brown, blue, green, and red; while dark shades were comprised of dark blue, dark green, dark red, and grey-black. The difference between light, medium, and dark shades among these years was significant (*χ*.^2^ = 16.0, df: 4, *p* = 0.003)

The greatest proportion of plastics observed in both 2021 and 2022 was light in hue (62.6–56.2%), with white plastic being the most frequent colour in this study (32.5–30.8%). The next most frequently encountered shade was medium-hued plastics (20.7–20.2%), with dark coloured plastics recovered least (16.8–23.6%). Over the sampled years analysed by this author (including previously published data for 2018 birds (Colvin et al. 2020), and this study during 2021 and 2022), there has been a significant decrease in the overall proportion of light-coloured plastics (− 14.1%), while ingestion of dark plastics has increased (+ 10.1%) (*χ*^2^ = 16.0, df: 4, *p* = 0.003) (Fig. 5, Appendix A-Table 10).

**Fig. 6:**
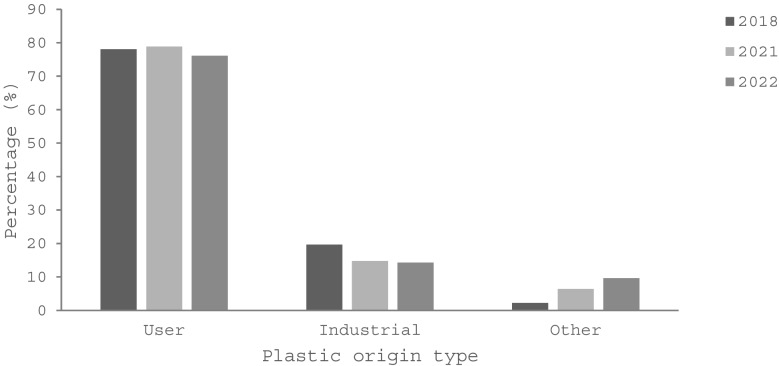
Percentage of ingested plastic types by their origin retained by short-tailed shearwater (*Ardenna tenuirostris*) fledglings collected on Phillip Island in 2018 (*n* = 310) (Colvin et al. 2020), 2021 (*n* = 203) and 2022 (*n* = 322) (this study). There was a continuous increase in other plastics and a decline in industrial plastics over the years sampled. The overall difference in plastic origin types was significant among the years analysed (*χ*.^2^ = 17.5, df: 4, *p* = 0.002)

Over the years of 2018, 2021, and 2022, ‘user plastics’ have been the most frequent type recovered from the stomachs, and ‘other plastics’ the least. From 2021 to 2022, user and industrial plastic declined from 78.8% to 76.1%, and from 14.8% to 14.3%, respectively. Other plastic rose from 6.4% to 9.6% over the same period. The difference in the plastic origin types was significant between the three years of 2018, 2021, and 2022 surveyed (*χ*^2^ = 17.5, df: 4, *p* = 0.002) (year 2018 proportions—user: 78.1%, industrial: 19.7%, other: 2.3%) (Fig. 6).

When classified by shape, fragments consisting of irregular particles made up the majority of the plastic recovered from the stomachs of the birds (2018: 78.4%, 2021: 83.7%, 2022: 83.2%). This was followed by pellets (2018: 14.2%, 2021: 13.3%, 2022: 10.9%), spherical beads (2018: 5.5%, 2021: 1.5%, 2022: 2.5%), and fibres (2018: 1.6%, 2021: 1.5%, 2022: 3.1%). Few to no foams were identified (2018: 0.3%, 2021: 0.0%, 2022: 0.3%) (2018: *n* = 310, 2021: *n* = 203, 2022: *n* = 322) (Table 6). There were no significant differences in the proportions of shape types recovered over the three years (*χ*^2^ = 12.0, df: 8, *p* = 0.2).

**Table 6:** The percentage of plastic particles by shape collected from the proventriculus and ventriculus of fledgling short-tailed shearwaters (*Ardenna tenuirostris*) located on Phillip Island during 2018 (*n* = 310) (Colvin et al. 2020), 2021 (*n* = 203), and 2022 (*n* = 322) (this study). The difference in the proportions of shapes was non-significant among the years analysed (*χ*^2^ = 12.0, df: 8, *p* = 0.2)

Plastic shape	2018 (%)	2021 (%)	2022 (%)
Fragment	78.4	83.7	83.2
Pellet	14.2	13.3	10.9
Bead	5.5	1.5	2.5
Fibre	1.6	1.5	3.1
Foam	0.3	0.0	0.3

### Correlations between plastic ingestion and body condition

**Fig. 7:**
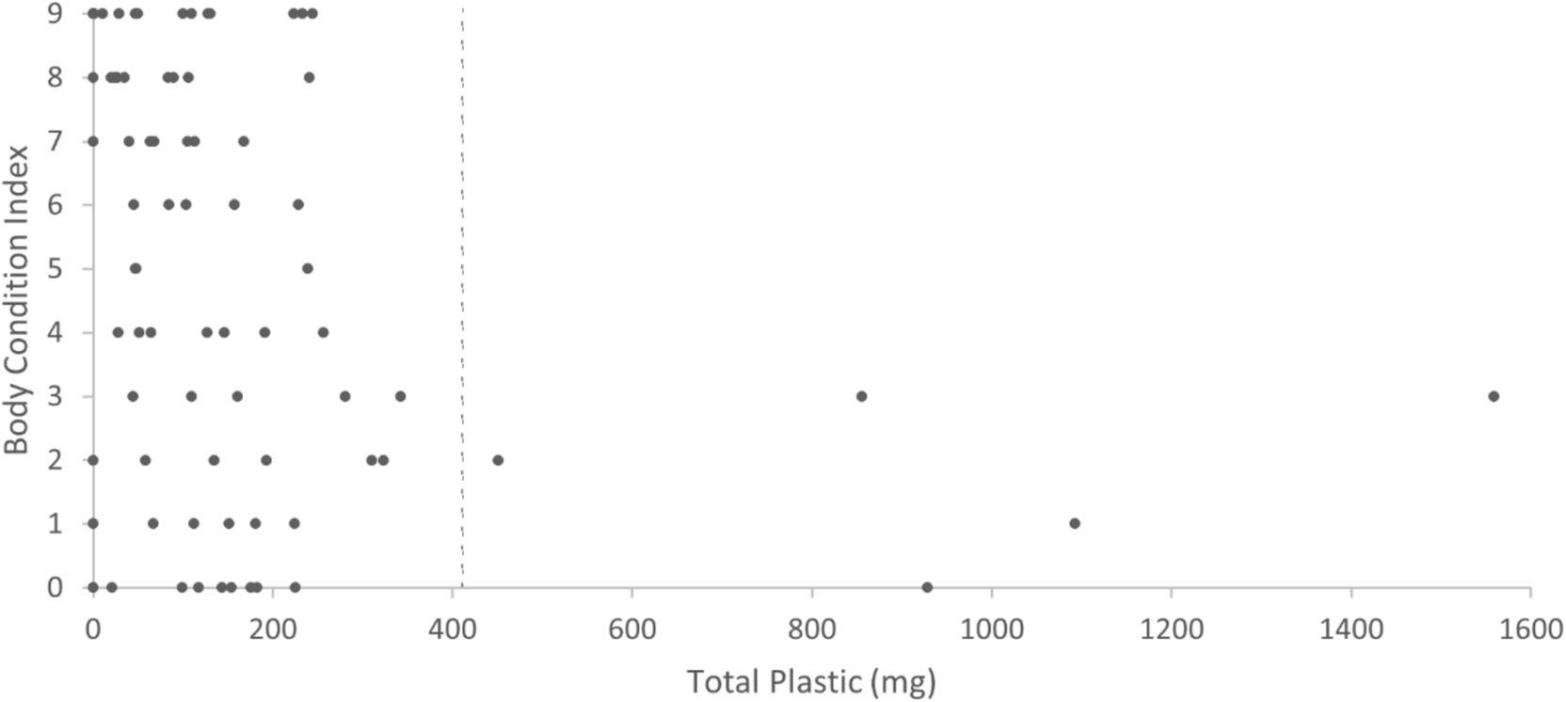
The Body Condition Index of short-tailed shearwater (*Ardenna tenuirostris*) fledglings on Phillip Island in 2021 and 2022 by their total mass of retained plastic (*n* = 76). All birds with ≥ 281 mg of plastic fell in the lower condition scores of 0–3. The dotted line at 412 mg total plastic indicates the threshold for high statistical outliers

The data from 2021 and 2022 was combined to investigate potential correlations of plastic characteristics with BCI. There was a significant inverse correlation between BCI and total mass of retained plastic (*τ*_b_ = − 0.2, *p* = 0.004, *n* = 76). All birds containing 281 mg or more of plastic had a BCI of 3 or below. While the correlation analysis found an overall inverse relationship, it is evident that there are two regions of different response, above and below a threshold level of retained plastic (Fig. 7). To identify the threshold, an outlier test was applied to the entire data set, which showed that all values above 412 mg of total retained plastic are statistical outliers. There was a stark difference between the BCI and the total mass of retained plastic above and below the identified threshold of 412 mg, with a mean BCI of 2 for birds above the threshold compared to a mean of 5 for birds below the threshold (*U* = 81.5, *p* = 0.04, *n* = 76). The variance in the high outlier group for plastic ingestion (*s*^2^ = 1.7) was significantly lower than that found in birds containing a smaller mass of plastic (*s*^2^ = 10.6) (*F* = 8.4, *p* = 0.005). There was no significant correlation between the BCI and plastic mass in the outlier group (*τ*_b_ = 0.1, *p* = 0.8, *n* = 5); however, a negative correlation remained in the birds with a lower mass of plastic ingestion (*τ*_b_ = − 0.2, *p* = 0.02, *n* = 71). If BCI was compared against the number of retained plastic pieces, the correlation was not significant (*τ*_b_ = − 0.1, *p* = 0.1, *n* = 76).

A threshold test identified proventriculus plastic over 174 mg as being high statistical outliers. All birds in this category were in the lower half of the BCI range with scores of 0–4 (mortally emaciated to lower moderate body condition.) A correlation between BCI and mass of only proventriculus plastic remained significant (*τ*_b_ = − 0.2, *p* = 0.02, *n* = 77), and the mean BCI was significantly lower for birds above (mean: 2, *n* = 9), compared to below the threshold (mean: 5, *n* = 68) (*U* = 141.0, *p* = 0.008). The variance in the high outlier group for proventriculus plastic (*s*^2^ = 2.0) was significantly lower than that found in birds containing a smaller mass of plastic (*s*^2^ = 10.6) (*F* = 13.6, *p* < 0.001). All but one of the high outliers for total retained plastic mass also had high proventriculus plastic loads. There was no significant correlation between BCI and ventriculus plastic mass (*τ*_b_ = − 0.1, *p* = 0.1, *n* = 76).

When the maximum dimension of the largest piece of retained plastic ingested by each bird was compared against BCI, no significant correlation was observed (*τ*_b_ = − 0.1, *p* = 0.2, *n* = 76). However, when highly flexible pieces (such as fibres and plastic film) were excluded from the analysis, a significant negative correlation with BCI was found (*τ*_b_ = − 0.2, *p* = 0.03, *n* = 76).

### Plastic ingestion and proventriculus ingesta amount

When birds from 2021 and 2022 were analysed together, they were found to have between 0 and 13,052 mg of ingesta within their proventriculus (*n* = 79). No significant correlations were seen between the BCI and the mass of ingesta remaining in the proventriculus at the time of death (total ingesta (*τ*_b_ = − 0.04, *p* = 0.6, *n* = 77), or the ingesta excluding plastic (*τ*_b_ = − 0.02, *p* = 0.8, *n* = 77). Significant positive correlations were calculated between the amount of plastic in the proventriculus and the total mass of ingesta in the proventriculus (plastic mass: *τ*_b_ = 0.4, *p* < 0.001, *n* = 79, number of pieces: *τ*_b_ = 0.3, *p* < 0.001, *n* = 79). This relationship remained significant even when only the non-plastic portion (i.e. food items, fluid, etc.) of the proventriculus ingesta was considered and compared to the amount of plastic in the proventriculus (plastic mass: *τ*_b_ = 0.3, *p* = 0.001, *n* = 79, number of pieces: *τ*_b_ = 0.3, *p* = 0.003, *n* = 79). There were no significant relationships between the mass of ingesta in the proventriculus and either the total retained plastic (plastic mass: *τ*_b_ = 0.2, *p* = 0.06, *n* = 78, number of pieces: *τ*_b_ = 0.09, *p* = 0.3, *n* = 78) or the ventriculus plastic loads (plastic mass: *τ*_b_ = − 0.06, *p* = 0.4, *n* = 78, number of pieces: *τ*_b_ = − 0.01, *p* = 0.9, *n* = 78). To identify a threshold, an outlier test was performed on the dataset for proventriculus plastic loads which showed that all values above 174 mg of plastic in the proventriculus are statistical outliers (Appendix A-Fig. 8). The mean mass of the entire proventriculus contents (which includes plastic if present) was significantly higher for the high outlier group (1320.7 mg ± 291.1, median: 1396.0 mg, min–max: 259.3–2469.4 mg, *n* = 9), compared to those with lower amounts of proventriculus plastic (mean: 1010.9 mg ± 248.9, median: 287.8 mg, min–max: 0–13052 mg, *n* = 70) (*U* = 161.0, *p* = 0.02, *n* = 79). However, when only the non-plastic portion of the ingesta was considered, the mean mass of the proventriculus contents was lower for birds in the high outlier group (mean: 734.6 mg ± 235.6, median: 535.0 mg, min–max: 2.5–2116.6 mg) compared to lower amounts of proventriculus plastic (mean: 988.9 mg ± 248.8, median: 260.8 mg, min–max: 0–13052 mg) (*U* = 239.0, *p* = 0.2, *n* = 79). The variance was lower for the mass of both the entire proventriculus contents and non-plastic portion of ingesta in birds within the high outlier group for proventriculus plastic loads, although this difference was not statistically significant (Entire proventriculus contents: high outlier proventriculus plastic *s*^2^ = 762,437.2, lower plastic *s*^2^ = 4,338,156.5 (*F* = 0.5, *p* = 0.5); non-plastic ingesta only: high outlier proventriculus plastic *s*^2^ = 499,598.5, lower plastic *s*^2^ = 4,334,341.1 (*F* = 1.2, *p* = 0.3)).

## Discussion

The goal of this research was to investigate the types of injuries and other pathological lesions present amongst beachcast fledgling short-tailed shearwaters and analyse whether the amount and characteristics of the retained ingested plastic had changed over time, and whether this affects the birds’ health. This study assessed data from the years 2018, 2021, and 2022 collected at Phillip Island, Victoria, Australia.

Although detailed reports of potential causes of pathological changes and deaths in the birds as a whole (not limited to examination of the gastrointestinal tract) via postmortem examination do not appear to be published for beachcast fledglings on Phillip Island, a frequent cause of death is thought to be due to wrecks at sea (Rodríguez et al. 2018) which was supported by a finding of 21 out of 66 birds in adequate condition to assess, being found to have aspirated water with drowning as a suspected cause of death, and a further six containing water in their airways with additional traumatic injuries. Shearwaters may also experience predation from animals including Pacific gulls (Leitch et al. 2014; Phillip Island Nature Parks 2023) which was observed to have occurred by the authors. It appears likely that predation is a common contributing cause of death, with twenty-eight birds displaying wounds consistent with attack by predatory birds and no obvious water in their airways. Seven birds had no evidence of trauma or water aspiration; however, all were wet and found near the shoreline indicating probable prior immersion in the ocean. Although a definite cause of death could not be established for these individuals, differentials could include drowning without apparent aspiration of water present on a gross examination (Lunetta et al. 2004), severe stress (Schmidt 1999), or hypothermia (Minka and Ayo 2017). Additional pathological findings likely to have contributed to reduced fitness and resulting mortality included: emaciation, chronic external injuries/infection (infected wounds and poorly healed limb fractures), metabolic bone disease, and one case of abnormal flight feather development which would have severely limited this bird’s ability to fly at the time of death. A definitive cause of the abnormal feather development in this bird is currently uncertain, but could include an infectious agent or an abnormal moult. One example of a virus that can result in abnormal feather development in birds includes circoviruses which although not recorded as affecting short-tailed shearwaters have been detected in a number of both psittacine and non-psittacine species (Doneley 2016; Haddadmarandi et al. 2020; Sarker et al. 2016). Although it is not impossible this was a yearling bird rather than a fledgling that never developed normal primary and secondary feathering, the finding is still abnormal given older birds should already have left the site and normal moulting of wing feathers is not known to occur during April/May (Marchant and Higgins 1990). All birds with inflammation or ulceration in their proventriculus contained plastic in this location, and with a single exception were classed as critically to mortally underweight. The ventriculus was the site of the majority of obstructions, with only one elsewhere occurring in the narrowed, caudal section of the proventriculus close to the isthmus, a site thought to be of increased risk for obstructions in procellariiforms due to their anatomical structure (Roman et al. 2019a). Plastic was obviously involved in five out of the seven cases of obstruction; however, other items including rocks, pumice, cuttlefish, shells, and kelp were also noted in cases of obstructive gastrointestinal disease.

Seabirds can be sensitive sentinels for monitoring plastic in the oceans (Ryan 2008). Overwhelmingly, plastic was the most frequent type of anthropogenic debris retained by sampled shearwaters at this location, with 87.5–94.7% of individuals shown to contain plastic in the sampled years of 2021 and 2022, respectively. Of significant concern is that intestinal fat stores have decreased each year assessed between 2018 and 2022 in the sampled birds, with a significant decrease overall, and between 2021 and 2022. The BCI, which takes into account three different measurements of body conditions (and measures ten categories of body condition rather than four) also showed a decrease from a mean of 5.7 in 2021 to 4.1 in 2022; however, this result was not found to be significant (*p* = 0.057). Given the near borderline significance level, a larger sample, or samples taken over a longer period of time (the greatest level of significance in changes in intestinal fat stores were seen when 2018, 2021, and 2022 results were assessed together) would be recommended to investigate these trends further at this location. Other studies have been conducted analysing the ingested plastic retained by beachcast fledgling short-tailed shearwaters in the same location at their time of migration departure in April–May, which can allow for longer monitoring of this area. When the mass of plastic retained in 2010 (Carey 2011) was compared to 2022, it showed an overall increase, although it is important to note there is yearly variation and it is not a continuous trend. Out of the years recorded at this location, the highest mass of plastic was noted in 2022, followed by 2015–16 (Rodríguez et al. 2018) (Table 2, Table 7) indicating that interannual changes in aspects such as food and plastic availability to the birds where they are choosing to feed may be contributing to variability in the retained plastic loads observed, as a continuous trend is not present. Continuing to monitor the colonies over a longer period of time would be advised. Although Rodríguez et al. (2018) indicated that body condition tends to be poorer and plastic ingestion higher in beachcast birds compared to those dying from other causes (e.g. road-kills), this current study remains a comparison of the same group type throughout this time frame and indicates a deterioration in mean body condition for birds dying on the beaches in this region. This may have additional concerns for this species, as they are required to complete a lengthy flight to the northern hemisphere, where weakness due to poor condition may place them at a disadvantage to successfully complete the migration (Rodríguez et al. 2017, 2018). The results of this paper indicate that an investigation of the colonies as a whole is vital to assess what proportion of the shearwaters are being affected by deteriorating body condition.

**Table 7:** Summary of additional published data regarding the percentage of beachcast fledgling short-tailed shearwaters (*Ardenna tenuirostris*) found to contain plastic in the gastrointestinal tract, and mean plastic ingestion per bird (mass (mg) and number of pieces ± standard deviation) over time at Phillip Island, Victoria (Carey 2011; Rodríguez et al. 2018)

Year	Source	Proportion ingesting plastic (%)	Pieces (mean number)	Pieces (min–max)	Mean mass (mg)	Mass (min–max)	*n*
2010	(Carey 2011)	100	7.6 ± 5.4	-	113 ± 8.2	-	67
2015–16	(Rodríguez et al. 2018)	100	8.23 ± 6.0	1- 26	240 ± 260	5.6–1047.2	26

Body condition is related to an animal’s health and quality, and is frequently used as an important determinant of fitness. Methods of determining body condition using the non-subjective parameters of mass and body lengths may be advantageous from a consistency point of view and due to body mass often having a relatively strong correlation with the mass of body fat; however, it is important that chosen methods are as independent as possible of factors such as gender and age that may otherwise confound comparisons. Additionally, body mass may be affected by variables such as hydration level and differences in the mass of the gastrointestinal contents (Labocha and Hayes 2012; Peig and Green 2010) which could be factors in the birds surveyed in this study, creating differences in body weight that are not related to their muscle size and fat stores (Labocha and Hayes 2012). Body condition scores relating to the appearance and fullness of fat and muscle are a subjective measure that have been utilised in seabirds, with variability minimised by researcher familiarity with the technique (Labocha and Hayes 2012; van Franeker 2004; Cousin et al. 2015). Variability was also reduced by using a single researcher to perform the subjective measurements for all birds within this study. Fat scores often correlate well with total body fat amount; however, this is not the case in every species and may present a possible limitation where correlations have not been confirmed. Although the mass of body fat is continuously distributed, fat scores fall into discrete, qualitative categories. Despite these potential downsides, the use of fat scores appears to correlate well with the mass of body fat, so their use is often seen as appropriate. Using subjective fat scores in models with body mass and morphological measurements may improve the ability to predict total body fat stores (Labocha and Hayes 2012); however, in this study, body mass would have been unable to be obtained accurately in many individuals due to scavenging, which made this measure unsuitable. Cousin et al. (2015) found that body mass was positively correlated with pectoral muscle, subcutaneous, and intestinal fat scores (*p* < 0.001) in short-tailed shearwater pre-fledgling chicks. The BCI combines these three separate measures to give a score between 0 and 9 and has been used when 2021 and 2022 years are being assessed alone for increased accuracy. Although extended analyses including the year 2018 only assess intestinal fat stores (due to a BCI being unavailable for 2018), this measurement was found to be highly correlated with measurements for subcutaneous fat and pectoral muscle, indicating similarities between the three scores if applied consistently.

When considering statistical testing, Kendal’s Tau was selected as part of the analysis for many datasets in this research, including assessing a correlation between BCI and the retained mass of plastic due to it being robust to outliers and appropriate for skewed and/or non-normally distributed data, which can affect many other standard tests (Helsel and Hirsch 1993). Additionally, noting significant correlations, such as that between plastic mass and BCI, graphing and visually assessing the data demonstrated when the possibility of a threshold effect should be investigated with further testing. All birds in this study containing at least 281 mg of plastic (9 out of 76) had a BCI of three or less, indicating they could be considered to be critically to mortally underweight. There are two different regions of response to plastic ingestion on body condition evident in Fig. 7. While neither region by itself shows a visual relationship between BCI and mass of plastic ingested, for birds containing high total levels of plastic (> 412 mg), the mean BCI and variance drop, indicating that all sampled birds in this category had a consistently low body condition. It is therefore possible to conclude from our results that higher plastic loads may be associated with an increased likelihood of poorer body condition. Puskic et al. (2025) proposed that a plastic load of at least 1–3% healthy body mass would be expected to cause negative impacts on body condition in flesh-footed shearwater chicks; however, in this sample of short-tailed shearwater fledglings, the threshold appeared to be lower. At fledging, the last weight in the burrow of short-tailed shearwaters has been recorded as approximately 616 g (Marchant and Higgins 1990), and at the Phillip Island colony Rodriguez et al. (2018) recorded road-killed birds to have a mean body mass of 523 g; however, our calculated threshold level for total plastic load was only 412 mg (~ 0.1% mean fledging body weight). All but one of the birds with high total plastic also had high levels of proventriculus plastic, indicating the amount of plastic retained in this location to be particularly relevant. The mass of plastic appeared to affect body condition more than the number of particles ingested in this study, suggesting plastic mass to be the more reliable indicator for assessing negative effects on individuals in this species.

Ingestion of plastic, which has no nutritional value, has been recorded as a likely contributing factor to decreased body condition in birds (Vlietstra and Parga 2002), and has been implicated in a number of negative health effects including inflammation, obstructions, and mechanical damage in the gastrointestinal tract (Pierce et al. 2004; Roman et al. 2019a), all of which were observed in this study. There was also an apparent reduction of the amount of actual food in the proventriculus when higher amounts of plastic were present in this location, a finding supported by Ryan (1988) who noted the plastic loaded birds in their experiment spent less time feeding and had a reduced food intake compared to the controls. The overall health and body condition of individual seabirds can be multifactorial, and other factors that can affect the health of wild birds and the ability of individual parents to supply sufficient nutrients to their offspring, which has been noted as a factor critical to successful fledging of their chicks (Lavers and Bond 2023; Puskic et al. 2025) may need to be considered. These include whether birds have access to sufficient amounts of regular prey items and access to non-nutritive items such as an increase in pumice ingestion noted in a significant starvation mortality event occurring in Australia during 2013 (Roman et al. 2021), the potential impact of changing conditions and events in the northern hemisphere (such as exposure to harmful algal blooms (2014–2017) (Van Hemert et al. 2021)), observations of late departures from Alaska due to weakness (2019) (Readfearn 2019), record breaking ocean temperatures in the Bering and eastern Chukchi Seas during 2017–2019 which caused changes to seabird distributions (including short-tailed shearwaters) possibly due to shifts in the location and availability of prey, and mortality events (Kuletz et al. 2020). Many of these factors could have affected the overall health and normal migration activity of the short-tailed shearwaters and amounts of plastic ingested (Roman et al. 2021; Van Hemert et al. 2021). In addition to the amount of food and non-nutritive items available to parent birds, their skill in hunting and ability to select for mostly food items will affect the growth and successful fledging of chicks (Puskic et al. 2025). Added to this, multiple factors may contribute to reducing the fitness of an individual while not necessarily being the sole cause of death, including subclinical changes that may not be evident from only measuring external morphometrics. Performing additional monitoring and blood testing in live birds, further testing such as full necropsies and histopathology in deceased animals, or dietary analysis in concert with analysis of the gastrointestinal plastic, may help with determining a more complete picture of what changes (both plastic and non-plastic related) are present. However, especially in the case of wild, unmonitored birds, all variables contributing to their health status can be difficult to account for (Garcês and Pires 2020; Gill et al. 2018; Harr 2006; Jara-Carrasco et al. 2015; Rivers-Auty et al. 2023),

Together with the mass of plastic, the maximum dimension of rigid plastics ingested also appears to be an important factor in body condition, with birds containing larger pieces of plastic by maximum dimension, correlating with a poorer BCI. A significant correlation was not observed if highly flexible plastics were included in the analysis, potentially because they may move more easily through narrowed areas of the gastrointestinal tract (such as from the proventriculus to the ventriculus, and the ventriculus to the intestines) in a way that would be impossible for larger rigid plastics over a certain size to pass through, potentially resulting in them being retained for longer. (The mean number of plastics in the proventriculus were fewer, yet heavier than that of the ventriculus, indicating larger pieces tended to be retained in the proventriculus). Larger pieces of rigid plastic were also observed to be involved in significant damage to the wall of the proventriculus in some birds such as seen in Fig. 2. Although ingestion of larger sized rigid plastics correlated with poorer BCI, it is worth noting that highly flexible plastics can also result in significant impacts on health. Examples include a death recorded by Roman et al. (2019a) due to obstruction of the intestines with a knotted balloon, and in this study an instance of ingested fishing line entangling other hard objects within the ventriculus resulting in a partial obstruction of the tract.

The mass of plastic present in the proventriculus appears to have the greatest effect on the total plastic loads per bird. Total plastic loads were highest in 2022, along with the largest recorded mean mass of plastic in the proventriculus and greatest percentage of birds retaining plastic in this location. Although differences were seen in the ventriculus plastic loads, they were smaller and not statistically significant. Given the proventriculus is relatively thin-walled and relies primarily on chemical digestion via the production of hydrochloric acid and pepsinogen, birds may have a limited ability to excrete plastic if it is unable to be passed through the narrow isthmus to the more muscular ventriculus, where mechanical grinding can occur (Koenig et al. 2016; Provencher et al. 2018) given the high resistance of many plastic types to hydrochloric acid (Evans 1966). This may thereby result in large pieces of plastic remaining in the proventriculus for prolonged periods of time unless regurgitated (Provencher et al. 2018). Although the increasing size of plastics over time is likely a factor in higher rates of retention within the proventriculus, it may not be the sole cause, given the mean size of plastics has risen on each recorded occasion between 2018 and 2022; however, the lowest plastic loads were noted in 2021. Additional contributing causes to the elevated plastic loads noted in 2022 could perhaps indicate the parent birds retaining more than typical amounts in their proventriculus, which is then passed to the chicks during feeding, or a greater amount of marine plastics available to the parents to feed their young when mistaken for food (Acampora et al. 2014; Carey 2011).

The mean and maximum size of plastics increased between 2018 and 2022, with a significant change in the mean maximum diameter occurring between 2018 and 2021. One reason behind the mean increase in plastic size in birds sampled at this site could be attributed in part to the decrease in industrial pellets (pre-production nurdles which are typically have diameters of 1–5 mm) observed between 2018 and 2022 (Colvin et al. 2020; NOAA 2024; Shugart et al. 2023); however, it does not appear to be the sole cause, as the maximum size of rigid plastic being detected has increased by 20.1 mm over this time, and the number of birds ingesting rigid plastic larger than 10.0 mm has risen by 48.1%. In a previous study by Colvin et al. (2020), it was uncommon for rigid plastics with a maximum dimension of more than 10 mm to be retained by short-tailed shearwater fledglings (max 10.4 mm), which agreed with Roman et al. (2019b) who indicated debris larger than 10 mm across are unlikely to be ingested by birds of this head size. By contrast, solid plastics of up to a maximum dimension of 30.5 mm were recovered from shearwaters in 2022, suggesting that the ingestion of large pieces of debris may not have been the birds’ preferred diet unless smaller items were unavailable.

Short-tailed shearwater adults start their migration earlier than their offspring, leaving the chicks to rely on their ingested food and fat stores to survive until fully fledged (Australian Government Australian Antartic Division 2010). This could potentially affect the body condition of individuals depending on the exact age of the bird, as chicks are thought to reach their maximum weight at approximately 50 to 75 days of age (mean mass 859.6 g), before losing weight as they approach fledging (mean last weight in burrow 615–617 g) (Marchant and Higgins 1990). While this may cause some variation between individuals within this study and possibly affect the mean body condition of birds at the peak time of departure as this date varies slightly from year to year, it does not appear to completely explain the decrease in intestinal fat stores between 2018 and 2022 as the earliest deceased bird ‘collection time period’ occurred in 2021. In addition, the amount of ingesta remaining in the proventriculus (which has some function for food storage as well as digestion (Shugart et al. 2023)) at the fledgling’s time of departure could be influenced by multiple factors including the amount fed, time elapsed since the last feed by the parents, gastrointestinal motility and capacity of the stomach until satiation. (Food intake can potentially be lowered by plastic in the gastrointestinal system reducing storage capacity, normal digestive processes and/or appetite). Normal passage of food from the stomach can be negatively affected by obstructive disease (Cornell and Koenig 2015; Furness 1985; Ryan 1988) which was observed in this study due to ingestion of both plastic and non-plastic objects. At lower proventriculus plastic loads, there was a wide range of ingesta amounts (0–13,052 mg) contained within their proventriculus likely because the birds had been able to eat freely if offered food by their parents. At high levels of plastic, the mean mass of the overall proventriculus contents (including plastic) is higher than that of birds with lower plastic loads. However, birds in the high outlier group also tended to have less actual food in their proventriculus than the birds with lower plastic loads. There could be a number of explanations for this including more plastic compared to food being fed to these fledglings by their parents, reduced stomach capacity to accept or retain offered food due to plastic occupying space in the stomach (Ryan 1988), or less inclination to accept offered food due to gastrointestinal discomfort or illness (Gelis 2006). (Five of the nine birds in the high outlier group for proventriculus plastic had irritation and/or ulcerations occurring in proventriculus lining on postmortem examination). Although a significant correlation was present when the non-plastic portion of the stomach contents were considered, there did not appear to be a strong enough difference to establish a significant threshold effect by the mass of ingested plastic. There was no indication that the amount of ingesta remaining in the proventriculus affected the body condition of fledglings within this study at the time they left for their migration; however, the presence of large amounts of plastic in the proventriculus reducing stomach capacity for food might still have the potential to result in negative impacts on juvenile survival rates as the birds begin to forage for themselves. Poor body condition has been linked to reduced nutrient intake in seabirds. Reduced stomach capacity caused by plastic within the gastrointestinal tracts of flesh-footed shearwaters has been proposed to be a significant factor in reducing their body condition and thereby their chances of survival, particularly for juveniles within their first few months at sea where their foraging efficiency may be decreased due to inexperience (Lavers et al. 2014).

The greatest proportion of ingested plastics between 2018 and 2022 has been light in hue, while dark coloured plastics were low. This suggests a colour preference by the birds, a finding mirrored in other studies on this species (Carey 2011; Vlietstra and Parga 2002). Without concurrent ocean trawls, it can be difficult to determine how much is selectivity vs. abundance in this study. There is support for the theory that short-tailed shearwaters are selecting potential prey based on visual clues, since Acampora et al. (2014) conducted necropsies concurrently with ocean trawls and found a significant difference in the colours obtained. Between 2018 and 2022, however, there was a significant difference in the hues of plastic being retrieved from the gastrointestinal tract, with a decrease in lighter plastics and an increase in dark colours. The cause of this is uncertain, but possibilities could relate to a proportional increase in the availability of darker hued debris in the water, or potentially even a change in prey availability that could have caused the birds to shift their colour preference to match their primary food sources in the area (Acampora et al. 2014; Ryan 2008; Vlietstra and Parga 2002). Day (1980) indicated that the colour of plastics consumed by seabirds may be influenced by the resemblance to features of the prey they hunt. Among the species he researched (which included short-tailed shearwaters), dark red particles were all ingested by those who ate crustaceans and proposed this was due to many crustaceans having eye colours of this shade, while dark plastic shades were more likely to be consumed by fish-feeders. By extension of this, it cannot be ruled out that alterations in the colour proportions might reflect a shift in diet (which in this species can include fish, cephalopods and krill) between chosen feeding locations or years (Day 1980; Marchant and Higgins 1990).

User plastic has remained the most common type ingested by short-tailed shearwaters at this location at all measured instances between 2018 and 2022; however, the change in the proportions of user, industrial, and other plastics over this time was significantly different. Fragments were the most frequently observed particle; however, this is likely to reflect the predominance of user plastic (which was mostly fragments) rather than selectivity for irregular shapes. This theory is also supported by reduced proportions of pellets being ingested as the density of industrial plastics compared to other categories of plastics appears to be decreasing in the oceans over time (Van Franeker and Law 2015; Vlietstra and Parga 2002). A decrease in ingested industrial plastic of 5.4% was observed between 2018 and 2022 and is a proportion not dissimilar to the drop in pellets and beads (the shapes most commonly associated with industrial plastic) of 6.3% over the same period. Reviewing data published by Carey (2011), the predominance of user plastic retained by these birds can also be seen to have occurred over the longer period of 13 years (2010 to 2022), with a decreasing trend of industrial (− 14.3%) and rising other plastics (+ 9.0%) over this period.

This research has sought to provide an overview of the types of gross pathological changes (both plastic and non-plastic) occurring in beachcast short-tailed shearwater fledglings and enable ongoing monitoring of plastic ingestion and body condition of these birds at Phillip Island, Australia. Although this research has revealed concerning information regarding declining body condition amongst the beachcast birds, a study of the colonies as a whole would be recommended to determine if and how this is affecting the entire population of short-tailed shearwaters (non-beachcast individuals and other age groups), or indeed other species of procellariiform birds that nest in this region as this may not be an isolated occurrence. Lavers and Bond (2023) noted a decline in body mass and size of flesh-footed shearwaters between 2010 and 2022. Additionally, this study indicated a deepening division over time between those chicks provided with sufficient nutrients by parents to fledge successfully, and those who could not, leading to beachcast birds being 30–60% smaller than individuals found in the general colony population. The increasingly poor condition of beachcast flesh-footed shearwaters (Lavers and Bond 2023) is also apparent in the deteriorating body condition of beachcast short-tailed shearwaters in this study, and it is currently unknown what consequences might result in the adult population given first breeding generally does not occur in short-tailed shearwaters until 5–8 years of age (Marchant and Higgins 1990). Additional beneficial information could also be obtained via monitoring of live birds and histopathology to more precisely establish causes of body metric and pathological changes. Plastic loads in these birds experienced yearly variation, with proventriculus loads appearing to most greatly influence the total plastic amount retained by these birds. There have been significant trends in certain characteristics of plastics during the period of the study with increasing maximum diameters, decreasing industrial/increasing other plastics, and alterations in colour with an increase of darker shades and decrease in light colours. The cause of this is uncertain and requires additional research but could relate to changing availability of plastic or food sources of the birds. Plastic was observed to have the potential to negatively affect these birds, in some cases causing direct damage or obstruction of the gastrointestinal tract. The presence of high amounts of plastic in the proventriculus tended to result in birds having less actual food in that location, which has potential implications for decreased stomach capacity or less actual food being fed to the chicks; however, this study was unable to find a link between the amount of food remaining in the proventriculus and body condition. Follow-up research investigating fledglings old enough to be feeding themselves may be beneficial to investigate this further. As these are wild, untagged birds, there are many different variables which can affect their body condition and general health which may not be able to be monitored or controlled; however, it is worth noting that over a critical mass of plastic, all birds in the study were in poor condition indicating that there may be a threshold effect for plastic loads in this and perhaps other species, over which birds are unlikely to thrive.

Supplementary information.

## Supplementary Information

Below is the link to the electronic supplementary material.

ESM 1 (DOCX 68.8 KB)

ESM 2 (DOCX 1.31 MB)

## Data Availability

The data that support the findings of this study are available from the corresponding author (J. Colvin) upon reasonable request.
