# Supplementary material for: Alterations in characteristics of plastic ingestion and decreasing body condition in beachcast fledgling short-tailed shearwaters (Ardenna tenuirostris) at Phillip Island, Australia
Source: Environ Sci Pollut Res Int. 2025 Jun 19;32(26):15877–95. doi: 10.1007/s11356-025-36643-6 (PMC12238070; doi:10.1007/s11356-025-36643-6)
Supplement: Supplementary file 1 — (DOCX 68.8 KB) [file 11356_2025_36643_MOESM1_ESM.docx]

**Supplementary Material**: Appendix A.

**Table 8** The number of birds each category (0-3) for intestinal fat, subcutaneous fat, pectoral muscle occurring in beachcast short-tailed shearwaters (*Ardenna tenuirostris*) collected on Phillip Island in 2018, 2021 and 2022.

| **Body condition score** | **n** | **0** | **1** | **2** | **3** |
| --- | --- | --- | --- | --- | --- |
| ***Intestinal Fat*** |  |  |  |  |  |
| 2018* | 52 | 2 | 2 | 17 | 31 |
| 2021 | 42 | 6 | 4 | 11 | 21 |
| 2022 | 42 | 9 | 14 | 8 | 11 |
| ***Subcutaneous Fat*** |  |  |  |  |  |
| 2021 | 42 | 7 | 8 | 12 | 15 |
| 2022 | 43 | 12 | 14 | 6 | 11 |
| ***Pectoral Muscle*** |  |  |  |  |  |
| 2021 | 41 | 5 | 10 | 14 | 12 |
| 2022 | 41 | 13 | 12 | 6 | 10 |

**Only intestinal fat scores are available for 2018.*

**Table 9** The number of birds each category (0-9) for the body condition index (BCI) occurring in beachcast short-tailed shearwaters (*Ardenna tenuirostris*) collected on Phillip Island in 2021 (n= 41) and 2022 (n=40).

| **BCI** | **0** | **1** | **2** | **3** | **4** | **5** | **6** | **7** | **8** | **9** |
| --- | --- | --- | --- | --- | --- | --- | --- | --- | --- | --- |
| 2021 | 4 | 3 | 0 | 3 | 3 | 2 | 5 | 5 | 9 | 7 |
| 2022 | 6 | 4 | 7 | 5 | 4 | 1 | 0 | 3 | 1 | 9 |

**Fig. 8** Mass of non-plastic proventriculus contents compared to the mass of retained ingested plastic in the proventriculus of fledgling short-tailed shearwaters (*Ardenna tenuirostris*) on Phillip Island in 2021 and 2022 (τb = 0.27, *p*= 0.001, n= 79). The dotted line at 174 mg proventriculus plastic indicates the threshold for high statistical outliers

**Table 10** The percentage of plastic particles by colour collected from the ventriculus and proventriculus of beachcast short-tailed shearwater (*Ardenna tenuirostris*) fledglings located on Phillip Island during 2018 (n=310) (Colvin et al. 2020)), 2021 (n= 203) and 2022 (n= 322) (This study).

| **Colour** | **2018 (%)** | **2021 (%)** | **2022 (%)** |
| --- | --- | --- | --- |
| Light |  |  |  |
| White | 45.81 | 32.51 | 30.75 |
| Yellow | 7.74 | 7.39 | 10.25 |
| Yellow-brown | 16.77 | 22.66 | 15.22 |
| *Total light* | *70.32* | *62.56* | *56.21* |
| Medium |  |  |  |
| Brown | 7.10 | 11.82 | 7.76 |
| Blue | 4.19 | 2.96 | 2.17 |
| Green | 2.58 | 4.43 | 8.39 |
| Red | 2.26 | 1.48 | 1.86 |
| *Total medium* | *16.13* | *20.69* | *20.19* |
| Dark |  |  |  |
| Dark blue | 1.29 | 1.48 | 0.93 |
| Dark green | 0.97 | 0.99 | 4.35 |
| Dark red | 0.32 | 2.46 | 3.11 |
| Grey-black | 10.97 | 11.82 | 15.22 |
| *Total dark* | *13.55* | *16.75* | *23.60* |
