# Supplementary material for: Alterations in characteristics of plastic ingestion and decreasing body condition in beachcast fledgling short-tailed shearwaters (Ardenna tenuirostris) at Phillip Island, Australia
Source: Environ Sci Pollut Res Int. 2025 Jun 19;32(26):15877–95. doi: 10.1007/s11356-025-36643-6 (PMC12238070; doi:10.1007/s11356-025-36643-6)
Supplement: Supplementary file 2 — (DOCX 1.31 MB) [file 11356_2025_36643_MOESM2_ESM.docx]

**Supplementary Material**: **Appendix B.**

**Summary of significant necropsy findings for beachcast short-tailed shearwaters *(Ardenna tenuirostris)* at Phillip Island, Victoria, Australia in 2022 (A) and 2021 (B).**

Site: Birds were collected from Woolamai beach on Phillip Island, Victoria, Australia, a surf beach with shoreline areas of sand and rocks. Short-tailed shearwaters nest in the sand dunes and cliffs along its length.

General necropsy notes: Presence of plastic was confirmed by microscope examination of the gastrointestinal contents as outlined in the methodology of the main paper. All birds were wet when collected unless otherwise stated. All birds showed evidence of probable recent feather development (presence of a feather sheath and/or blood in the feather shaft) of at least some primary/secondary flight feathers. Necropsy codes are modified from van Franeker (2004).

*Freshness codes- **FFF:** “Very fresh”. Eyes bright and shiny. **FF:** “Fresh”. Eyes dull and a bit shrunken, but tissue in mouth looks fresh and not discoloured. FF was also used if mild autolysis of organs was noted even in the absence of eye changes.

*Completeness codes- **CC:** “Complete”. Body and plumage fully intact with no scavenging. **C:** “Near complete”. Lightly damaged or scavenged, but major feather areas are present. Organs are undamaged to mostly intact. **I:** “Incomplete”. Serious damage or scavenging. Major feather areas or some organs may be significantly damaged or missing. **II:** “Parts only”. Whole body sections missing. (e.g. Only wings and breastbone recovered.)

**A) Necropsy reports for the year 2022.**

**SH-PI-22-1**

| **Collection Year** | | | 2022 | | | | | | | **Bird ID** | | | | SH-PI-22-1 | | | | | |
| --- | --- | --- | --- | --- | --- | --- | --- | --- | --- | --- | --- | --- | --- | --- | --- | --- | --- | --- | --- |
| **Freshness** | | | FFF (Very fresh) | | | | | | | **Completeness** | | | | I | | | | | |
| **Measurements** | | Culmen length | | | | | | | 3.3 | | | Bill width | | | | | 0.65 | | |
| **(cm)** | | Bill depth | | | | | | | 0.9 | | | Head length | | | | | - | | |
|  | | Tarsometatarsus length | | | | | | | 5.0 | | | Radius/Ulna length | | | | | 9.7 | | |
|  | | Heart base-apex | | | | | | | - | | |  | | | | |  | | |
| **Body Condition** | Intestinal fat (0-3) | | | | 2 | Subcutaneous fat (0-3) | | | | | 2 | Breast muscle (0-3) | | | - | BCI (0-9) | | | **-** |
| **Gastrointestinal plastic?** | | Proventriculus | | | | | Y | Ventriculus | | | | Y | **Bursa of Fabricius?** | | | | | Y | |
| **Complete GIT?** | | Y | | **If no, details:** | | | | | | | | | | | | | | | |

**Summary of abnormal/significant findings if present:**

**Scavenging:** Left lung removed, partially scavenged brain and most of breast muscle removed. Fractured skull.

**Internal:** Wet heavy right lung, with obvious water in lung/air sacs indicating likely pre-mortem aspiration of water. Only a small amount of material in the proventriculus, much of which was plastic + a tiny amount of feathers fragments.

**SH-PI-22-2**

| **Collection Year** | | | 2022 | | | | | | | **Bird ID** | | | | SH-PI-22-2 | | | | | |
| --- | --- | --- | --- | --- | --- | --- | --- | --- | --- | --- | --- | --- | --- | --- | --- | --- | --- | --- | --- |
| **Freshness** | | | FFF (Very fresh) | | | | | | | **Completeness** | | | | I | | | | | |
| **Measurements** | | Culmen length | | | | | | | 3.1 | | | Bill width | | | | | 0.65 | | |
| **(cm)** | | Bill depth | | | | | | | 0.9 | | | Head length | | | | | - | | |
|  | | Tarsometatarsus length | | | | | | | 5.2 | | | Radius/Ulna length | | | | | 10.5 | | |
|  | | Heart base-apex | | | | | | | - | | |  | | | | |  | | |
| **Body Condition** | Intestinal fat (0-3) | | | | 3 | Subcutaneous fat (0-3) | | | | | 3 | Breast muscle (0-3) | | | 3 | BCI (0-9) | | | **9** |
| **Gastrointestinal plastic?** | | Proventriculus | | | | | N | Ventriculus | | | | Y | **Bursa of Fabricius?** | | | | | Y | |
| **Complete GIT?** | | Y | | **If no, details:** | | | | | | | | | | | | | | | |

**Summary of abnormal/significant findings if present:**

**Scavenging:** Damage to the right chest area with right lung and liver removed. Damage to heart.

**External:** Fat, large, well developed bird.

**Internal:** No obvious water in left lung, however significant damage in mid to upper coelomic cavity including air sacs and heart due to scavenging and the right lung was missing. Empty proventriculus. Ventriculus contained plastic, several squid beaks, a rock, and a small amount of sand.

**SH-PI-22-3**

| **Collection Year** | | | 2022 | | | | | | | **Bird ID** | | | | SH-PI-22-3 | | | | | |
| --- | --- | --- | --- | --- | --- | --- | --- | --- | --- | --- | --- | --- | --- | --- | --- | --- | --- | --- | --- |
| **Freshness** | | | FFF (Very fresh) | | | | | | | **Completeness** | | | | I | | | | | |
| **Measurements** | | Culmen length | | | | | | | 3.4 | | | Bill width | | | | | 0.6 | | |
| **(cm)** | | Bill depth | | | | | | | 0.9 | | | Head length | | | | | - | | |
|  | | Tarsometatarsus length | | | | | | | 5.3 | | | Radius/Ulna length | | | | | 9.9 | | |
|  | | Heart base-apex | | | | | | | - | | |  | | | | |  | | |
| **Body Condition** | Intestinal fat (0-3) | | | | 2 | Subcutaneous fat (0-3) | | | | | 2 | Breast muscle (0-3) | | | - | BCI (0-9) | | | **-** |
| **Gastrointestinal plastic?** | | Proventriculus | | | | | - | Ventriculus | | | | - | **Bursa of Fabricius?** | | | | | Y | |
| **Complete GIT?** | | N | | **If no, details:** Missing lower oesophagus, proventriculus, ventriculus and some of intestines. | | | | | | | | | | | | | | | |

**Summary of abnormal/significant findings if present:**

**Scavenging:** Badly scavenged with the proventriculus, ventriculus, upper intestines and breast muscle missing. (No plastic analysis done due to this.) Skull fractured and brain removed. Left lung and kidney removed. Mild damage to liver.

**External:** Fairly large bird.

**Internal:** No obvious water in the airways, however there was significant damage due to scavenging present including a missing lung making this difficult to confirm. No plastic analysis due to scavenged gastrointestinal tract.

**SH-PI-22-4**

| **Collection Year** | | | 2022 | | | | | | | **Bird ID** | | | | SH-PI-22-4 | | | | | |
| --- | --- | --- | --- | --- | --- | --- | --- | --- | --- | --- | --- | --- | --- | --- | --- | --- | --- | --- | --- |
| **Freshness** | | | FFF (Very fresh) | | | | | | | **Completeness** | | | | I | | | | | |
| **Measurements** | | Culmen length | | | | | | | 3.35 | | | Bill width | | | | | 0.6 | | |
| **(cm)** | | Bill depth | | | | | | | 0.9 | | | Head length | | | | | - | | |
|  | | Tarsometatarsus length | | | | | | | 5.1 | | | Radius/Ulna length | | | | | 9.7 | | |
|  | | Heart base-apex | | | | | | | - | | |  | | | | |  | | |
| **Body Condition** | Intestinal fat (0-3) | | | | 0 | Subcutaneous fat (0-3) | | | | | 0 | Breast muscle (0-3) | | | 0 | BCI (0-9) | | | **0** |
| **Gastrointestinal plastic?** | | Proventriculus | | | | | N | Ventriculus | | | | Y | **Bursa of Fabricius?** | | | | | Y | |
| **Complete GIT?** | | Y | | **If no, details:** | | | | | | | | | | | | | | | |

**Summary of abnormal/significant findings if present:**

**Scavenging:** Brain removed.

**External:** Emaciated. Puncture wound in the right chest area into the coelomic cavity with evidence of bleeding. Skull had been fractured (frontal and parietal bones). Peck marks on head.

**Internal:** No obvious water in airways. Small amount of ingesta in the proventriculus. Ventriculus felt firmly packed and full of ingesta which contains plastic. Reasonable amount of ingesta in the intestines.

**SH-PI-22-5**

| **Collection Year** | | | 2022 | | | | | | | **Bird ID** | | | | SH-PI-22-5 | | | | | |
| --- | --- | --- | --- | --- | --- | --- | --- | --- | --- | --- | --- | --- | --- | --- | --- | --- | --- | --- | --- |
| **Freshness** | | | FFF (Very fresh) | | | | | | | **Completeness** | | | | CC | | | | | |
| **Measurements** | | Culmen length | | | | | | | 3.2 | | | Bill width | | | | | 0.6 | | |
| **(cm)** | | Bill depth | | | | | | | 0.85 | | | Head length | | | | | - | | |
|  | | Tarsometatarsus length | | | | | | | 5.05 | | | Radius/Ulna length | | | | | 9.4 | | |
|  | | Heart base-apex | | | | | | |  | | |  | | | | |  | | |
| **Body Condition** | Intestinal fat (0-3) | | | | 1 | Subcutaneous fat (0-3) | | | | | 1 | Breast muscle (0-3) | | | 0 | BCI (0-9) | | | **2** |
| **Gastrointestinal plastic?** | | Proventriculus | | | | | N | Ventriculus | | | | N | **Bursa of Fabricius?** | | | | | Y | |
| **Complete GIT?** | | Y | | **If no, details:** | | | | | | | | | | | | | | | |

**Summary of abnormal/significant findings if present:**

**Scavenging:** Not scavenged.

**External:** Very thin. Skull fracture (frontal and parietal) with haemorrhage present. Uncertain cause of skull fracture (predator vs impact of other nature.)

**Internal:** Very poor muscling and low amounts of body fat. Liver was on the small side, but normal in colour. Lungs were dense and wet and indicate likely pre-mortem aspiration of water. A moderate amount of green ingesta containing lots of downy feathers was in the proventriculus. A small amount of green ingesta was in the ventriculus and contained squid beaks and a piece of cuttlefish.

**SH-PI-22-6**

| **Collection Year** | | | 2022 | | | | | | | **Bird ID** | | | | SH-PI-22-6 | | | | | |
| --- | --- | --- | --- | --- | --- | --- | --- | --- | --- | --- | --- | --- | --- | --- | --- | --- | --- | --- | --- |
| **Freshness** | | | FFF (Very fresh) | | | | | | | **Completeness** | | | | C | | | | | |
| **Measurements** | | Culmen length | | | | | | | 3.0 | | | Bill width | | | | | 0.75 | | |
| **(cm)** | | Bill depth | | | | | | | 0.8 | | | Head length | | | | | - | | |
|  | | Tarsometatarsus length | | | | | | | 4.8 | | | Radius/Ulna length | | | | | 9.3 | | |
|  | | Heart base-apex | | | | | | | - | | |  | | | | |  | | |
| **Body Condition** | Intestinal fat (0-3) | | | | 0 | Subcutaneous fat (0-3) | | | | | 0 | Breast muscle (0-3) | | | 1 | BCI (0-9) | | | **1** |
| **Gastrointestinal plastic?** | | Proventriculus | | | | | Y | Ventriculus | | | | Y | **Bursa of Fabricius?** | | | | | Y | |
| **Complete GIT?** | | Y | | **If no, details:** | | | | | | | | | | | | | | | |

**Summary of abnormal/significant findings if present:**

**Scavenging:** Minor scavenging of breast muscle.

**External:** Emaciated.

**Internal:** No obvious water in airways. Puncture hole into the coelomic cavity in right chest area with evidence of haemorrhage/bleeding in muscle on right chest. Green ingesta containing plastic and a number of small rocks was present in the proventriculus. The ventriculus was very full and contained many rocks and pieces of plastic.

**SH-PI-22-7**

| **Collection Year** | | | 2022 | | | | | | | **Bird ID** | | | | SH-PI-22-7 | | | | | |
| --- | --- | --- | --- | --- | --- | --- | --- | --- | --- | --- | --- | --- | --- | --- | --- | --- | --- | --- | --- |
| **Freshness** | | | FFF (Very fresh) | | | | | | | **Completeness** | | | | I | | | | | |
| **Measurements** | | Culmen length | | | | | | | 2.9 | | | Bill width | | | | | 0.6 | | |
| **(cm)** | | Bill depth | | | | | | | 0.85 | | | Head length | | | | | - | | |
|  | | Tarsometatarsus length | | | | | | | 4.9 | | | Radius/Ulna length | | | | | 9.4 | | |
|  | | Heart base-apex | | | | | | | - | | |  | | | | |  | | |
| **Body Condition** | Intestinal fat (0-3) | | | | 0 | Subcutaneous fat (0-3) | | | | | 0 | Breast muscle (0-3) | | | 0 | BCI (0-9) | | | **0** |
| **Gastrointestinal plastic?** | | Proventriculus | | | | | Y | Ventriculus | | | | Y | **Bursa of Fabricius?** | | | | | Y | |
| **Complete GIT?** | | Y | | **If no, details:** | | | | | | | | | | | | | | | |

**Summary of abnormal/significant findings if present:**

**Scavenging:** Brain removed.

**External:** Emaciated. Fractured skull (frontal and parietal bones) with haemorrhage. Peck marks on head.

**Internal:** Heart chambers distended with blood. Liver a bit enlarged with rounded edges and mild lighter blotchiness in colouration. No obvious water in the airways, however a small amount of blood was present in the airways, mainly in the abdominal air sacs. Proventriculus contained almost no food, only plastic. Ventriculus contained mainly plastic with some rocks and squid beaks identifiable.

**SH-PI-22-8**

| **Collection Year** | | | 2022 | | | | | | | **Bird ID** | | | | SH-PI-22-8 | | | | | |
| --- | --- | --- | --- | --- | --- | --- | --- | --- | --- | --- | --- | --- | --- | --- | --- | --- | --- | --- | --- |
| **Freshness** | | | FF (Fresh) | | | | | | | **Completeness** | | | | C | | | | | |
| **Measurements** | | Culmen length | | | | | | | 3.4 | | | Bill width | | | | | 0.65 | | |
| **(cm)** | | Bill depth | | | | | | | 0.8 | | | Head length | | | | | - | | |
|  | | Tarsometatarsus length | | | | | | | 4.8 | | | Radius/Ulna length | | | | | 9.25 | | |
|  | | Heart base-apex | | | | | | | - | | |  | | | | |  | | |
| **Body Condition** | Intestinal fat (0-3) | | | | 1 | Subcutaneous fat (0-3) | | | | | 0 | Breast muscle (0-3) | | | 0 | BCI (0-9) | | | **1** |
| **Gastrointestinal plastic?** | | Proventriculus | | | | | N | Ventriculus | | | | Y | **Bursa of Fabricius?** | | | | | Y | |
| **Complete GIT?** | | Y | | **If no, details:** | | | | | | | | | | | | | | | |

**Summary of abnormal/significant findings if present:**

**Scavenging:** Some removal of muscle and skin in the right chest area. Suspect this bird may have died on the previous day due to scavenging damage being present even though this animal was collected before dawn and mild autolytic changes were present in the ventriculus.

**External:** Emaciated. Skin trauma right chest (peck marks/tearing.)

**Internal:** Trauma to right chest area (skin and muscle) most consistent with a predatory bird. No obvious water in airways. Liver on the small side. Tissues appeared to have minimal obvious autolysis apart from the lining of the ventriculus. Proventriculus contained a fairly large piece of cuttlefish, some feather fragments, green ingesta and thick mucus like material. Ventriculus contained plastic, squid beaks, a rock and a small amount of other ingesta.

**SH-PI-22-9**

| **Collection Year** | | | 2022 | | | | | | | **Bird ID** | | | | SH-PI-22-9 | | | | | |
| --- | --- | --- | --- | --- | --- | --- | --- | --- | --- | --- | --- | --- | --- | --- | --- | --- | --- | --- | --- |
| **Freshness** | | | FFF (Very fresh) | | | | | | | **Completeness** | | | | C | | | | | |
| **Measurements** | | Culmen length | | | | | | | 3.2 | | | Bill width | | | | | 0.6 | | |
| **(cm)** | | Bill depth | | | | | | | 0.8 | | | Head length | | | | | - | | |
|  | | Tarsometatarsus length | | | | | | | 4.8 | | | Radius/Ulna length | | | | | 9.8 | | |
|  | | Heart base-apex | | | | | | | - | | |  | | | | |  | | |
| **Body Condition** | Intestinal fat (0-3) | | | | 2 | Subcutaneous fat (0-3) | | | | | 1 | Breast muscle (0-3) | | | 1 | BCI (0-9) | | | **4** |
| **Gastrointestinal plastic?** | | Proventriculus | | | | | Y | Ventriculus | | | | Y | **Bursa of Fabricius?** | | | | | Y | |
| **Complete GIT?** | | Y | | **If no, details:** | | | | | | | | | | | | | | | |

**Summary of abnormal/significant findings if present:**

**Scavenging:** Minimal. Minor soft tissue removal around the head.

**External:** Low-moderate body condition. Bruising and bleeding relating to skin wounds (mostly small peck like marks) about the head. Blood was present in the nose and mouth.

**Internal:** Intracranial haemorrhages. Lungs are very wet containing watery blood. Watery blood was also present in the trachea and throughout the air sacs. (Likely aspiration of water and blood.) Liver was tan-yellow in colour, otherwise normal in appearance. Heart was large and chambers are distended with blood. Proventriculus contained a mucous like material, plastic (including a large piece) and green ingesta with feather fragments. Ventriculus contained numerous small rocks and four pieces of plastic.

**SH-PI-22-10**

| **Collection Year** | | | 2022 | | | | | | | **Bird ID** | | | | SH-PI-22-10 | | | | | |
| --- | --- | --- | --- | --- | --- | --- | --- | --- | --- | --- | --- | --- | --- | --- | --- | --- | --- | --- | --- |
| **Freshness** | | | FFF (Very fresh) | | | | | | | **Completeness** | | | | I | | | | | |
| **Measurements** | | Culmen length | | | | | | | - | | | Bill width | | | | | - | | |
| **(cm)** | | Bill depth | | | | | | | - | | | Head length | | | | | - | | |
|  | | Tarsometatarsus length | | | | | | | 5.1 | | | Radius/Ulna length | | | | | 10.1 | | |
|  | | Heart base-apex | | | | | | | - | | |  | | | | |  | | |
| **Body Condition** | Intestinal fat (0-3) | | | | 3 | Subcutaneous fat (0-3) | | | | | 3 | Breast muscle (0-3) | | | 3 | BCI (0-9) | | | **9** |
| **Gastrointestinal plastic?** | | Proventriculus | | | | | N | Ventriculus | | | | Y | **Bursa of Fabricius?** | | | | | Y | |
| **Complete GIT?** | | Y | | **If no, details:** | | | | | | | | | | | | | | | |

**Summary of abnormal/significant findings if present:**

**Scavenging:** The head was severely scavenged with the top of the skull and beak missing. Brain scavenged. The rest of the body was intact.

**External:** Large well grown appearing bird in fat body condition. Severe damage to head with scavenging evident. Some bleeding. A few feather lice present.

**Internal:** No obvious water in airways. Proventriculus empty of food or plastic. Ventriculus contained 10 pieces of plastic, algae, squid beaks and cuttlefish.

**SH-PI-22-11**

| **Collection Year** | | | 2022 | | | | | | | **Bird ID** | | | | SH-PI-22-11 | | | | | |
| --- | --- | --- | --- | --- | --- | --- | --- | --- | --- | --- | --- | --- | --- | --- | --- | --- | --- | --- | --- |
| **Freshness** | | | FF (Fresh) | | | | | | | **Completeness** | | | | I | | | | | |
| **Measurements** | | Culmen length | | | | | | | - | | | Bill width | | | | | - | | |
| **(cm)** | | Bill depth | | | | | | | - | | | Head length | | | | | - | | |
|  | | Tarsometatarsus length | | | | | | | 4.8 | | | Radius/Ulna length | | | | | 9.75 | | |
|  | | Heart base-apex | | | | | | | 2.2 | | |  | | | | |  | | |
| **Body Condition** | Intestinal fat (0-3) | | | | 0 | Subcutaneous fat (0-3) | | | | | 0 | Breast muscle (0-3) | | | 0 | BCI (0-9) | | | **0** |
| **Gastrointestinal plastic?** | | Proventriculus | | | | | Y | Ventriculus | | | | Y | **Bursa of Fabricius?** | | | | | Y | |
| **Complete GIT?** | | Y | | **If no, details:** | | | | | | | | | | | | | | | |

**Summary of abnormal/significant findings if present:**

**Scavenging:** Most of head missing including beak. Brain scavenged. Removal some soft tissues (predominantly breast muscle) over the chest opening the coelomic cavity in this area. Damage to air sacs present in cranial chest area.

**External:** Emaciated. Primary wing feathers were small but are not blood feathers. Feather sheath still covered some of the barbs apart from the most distal primary feather on each wing.

**Internal:** Heart and liver were on the smaller side. Lungs were very wet and contained water. Kidneys were friable and appear likely to be mildly autolysed. The distal proventriculus had a 1cm diameter ulcer in the mucosa (not perforated.) A large piece of plastic was located against the area of the ulceration and had fibrinous attachments anchoring the plastic to the wall of the proventriculus. The area appeared inflamed with increased erythema. Minimal food in proventriculus, mucous like material was present. Ventriculus had pieces of what most resembled fishing line entangled around ingesta and debris including two rocks and a shell. This was likely interfering with normal digestion and passage of food from the ventriculus.

**SH-PI-22-12**

| **Collection Year** | | | 2022 | | | | | | | **Bird ID** | | | | SH-PI-22-12 | | | | | |
| --- | --- | --- | --- | --- | --- | --- | --- | --- | --- | --- | --- | --- | --- | --- | --- | --- | --- | --- | --- |
| **Freshness** | | | FFF (Very fresh) | | | | | | | **Completeness** | | | | CC | | | | | |
| **Measurements** | | Culmen length | | | | | | | 3.5 | | | Bill width | | | | | 0.7 | | |
| **(cm)** | | Bill depth | | | | | | | 0.9 | | | Head length | | | | | - | | |
|  | | Tarsometatarsus length | | | | | | | 5.0 | | | Radius/Ulna length | | | | | 9.8 | | |
|  | | Heart base-apex | | | | | | | 2.1 | | |  | | | | |  | | |
| **Body Condition** | Intestinal fat (0-3) | | | | 1 | Subcutaneous fat (0-3) | | | | | 1 | Breast muscle (0-3) | | | 0 | BCI (0-9) | | | **2** |
| **Gastrointestinal plastic?** | | Proventriculus | | | | | Y | Ventriculus | | | | Y | **Bursa of Fabricius?** | | | | | Y | |
| **Complete GIT?** | | Y | | **If no, details:** | | | | | | | | | | | | | | | |

**Summary of abnormal/significant findings if present:**

**Scavenging:** Not Scavenged.

**External:** Very thin. Bruising on the right side of the head from unknown cause of trauma. Quite a bit of blood remaining in the feather shafts of primary wing feathers.

**Internal:** No obvious fluid in airways. Free blood was present in the cranial coelomic cavity. Heart was on the smaller side. A large piece of plastic and three wooden fragments were in the proventriculus which was empty otherwise. Shallow ulcer was present in the proventriculus 0.75cm x 0.5cm diameter. Ventriculus contained a fair amount of ingesta with a piece of plastic.

**SH-PI-22-13**

| **Collection Year** | | | 2022 | | | | | | | **Bird ID** | | | | SH-PI-22-13 | | | | | |
| --- | --- | --- | --- | --- | --- | --- | --- | --- | --- | --- | --- | --- | --- | --- | --- | --- | --- | --- | --- |
| **Freshness** | | | FFF (Very fresh) | | | | | | | **Completeness** | | | | I | | | | | |
| **Measurements** | | Culmen length | | | | | | | 3.0 | | | Bill width | | | | | 0.5 | | |
| **(cm)** | | Bill depth | | | | | | | 0.8 | | | Head length | | | | | 7.45 | | |
|  | | Tarsometatarsus length | | | | | | | 4.85 | | | Radius/Ulna length | | | | | 9.8 | | |
|  | | Heart base-apex | | | | | | | - | | |  | | | | |  | | |
| **Body Condition** | Intestinal fat (0-3) | | | | 0 | Subcutaneous fat (0-3) | | | | | 1 | Breast muscle (0-3) | | | 1 | BCI (0-9) | | | **2** |
| **Gastrointestinal plastic?** | | Proventriculus | | | | | Y | Ventriculus | | | | Y | **Bursa of Fabricius?** | | | | | Y | |
| **Complete GIT?** | | Y | | **If no, details:** | | | | | | | | | | | | | | | |

**Summary of abnormal/significant findings if present:**

**Scavenging:** Some scavenging of breast muscle with the coelomic cavity opened and damage to air sacs. Part of heart removed, mild lung damage.

**External:** Very thin.

**Internal:** Free watery bloody fluid was present in the coelomic cavity. Degree of scavenging and location on shore makes it difficult to be certain if water entered before or after death on exam. Plastic in proventriculus (including large pieces) and squid beaks identified in ingesta. Ventriculus had large number of plastic pieces and ingesta containing squid beaks. Quite a bit of blood remaining in the immature primary flight feather shafts.

**SH-PI-22-14**

| **Collection Year** | | | 2022 | | | | | | | **Bird ID** | | | | SH-PI-22-14 | | | | | |
| --- | --- | --- | --- | --- | --- | --- | --- | --- | --- | --- | --- | --- | --- | --- | --- | --- | --- | --- | --- |
| **Freshness** | | | FFF (Very fresh) | | | | | | | **Completeness** | | | | C | | | | | |
| **Measurements** | | Culmen length | | | | | | | 3.2 | | | Bill width | | | | | 0.6 | | |
| **(cm)** | | Bill depth | | | | | | | 0.9 | | | Head length | | | | | 7.6 | | |
|  | | Tarsometatarsus length | | | | | | | 4.7 | | | Radius/Ulna length | | | | | 9.5 | | |
|  | | Heart base-apex | | | | | | | 2.5 | | |  | | | | |  | | |
| **Body Condition** | Intestinal fat (0-3) | | | | 1 | Subcutaneous fat (0-3) | | | | | 0 | Breast muscle (0-3) | | | 0 | BCI (0-9) | | | **1** |
| **Gastrointestinal plastic?** | | Proventriculus | | | | | Y | Ventriculus | | | | Y | **Bursa of Fabricius?** | | | | | Y | |
| **Complete GIT?** | | Y | | **If no, details:** | | | | | | | | | | | | | | | |

**Summary of abnormal/significant findings if present:**

**Scavenging:** Fractured skull with open cranial cavity and some damage to brain.

**External:** Emaciated.

**Internal:** Lungs heavy and wet indicating likely aspiration of water. Proventriculus empty apart from a piece of plastic. Ventriculus contains many plastic pieces.

**SH-PI-22-15**

| **Collection Year** | | | 2022 | | | | | | | **Bird ID** | | | | SH-PI-22-15 | | | | | |
| --- | --- | --- | --- | --- | --- | --- | --- | --- | --- | --- | --- | --- | --- | --- | --- | --- | --- | --- | --- |
| **Freshness** | | | FF (Fresh) | | | | | | | **Completeness** | | | | I | | | | | |
| **Measurements** | | Culmen length | | | | | | | 3.3 | | | Bill width | | | | | 0.7 | | |
| **(cm)** | | Bill depth | | | | | | | 0.8 | | | Head length | | | | | 8.1 | | |
|  | | Tarsometatarsus length | | | | | | | 5.4 | | | Radius/Ulna length | | | | | 10.4 | | |
|  | | Heart base-apex | | | | | | | - | | |  | | | | |  | | |
| **Body Condition** | Intestinal fat (0-3) | | | | 3 | Subcutaneous fat (0-3) | | | | | 3 | Breast muscle (0-3) | | | 3 | BCI (0-9) | | | **9** |
| **Gastrointestinal plastic?** | | Proventriculus | | | | | N | Ventriculus | | | | Y | **Bursa of Fabricius?** | | | | | Y | |
| **Complete GIT?** | | Y | | **If no, details:** | | | | | | | | | | | | | | | |

**Summary of abnormal/significant findings if present:**

**Scavenging:** Soft tissue scavenged in neck. Damage to heart. Uncertain if all damage was postmortem.

**External:** Fat body condition. Fairly large well grown bird with primary flight feathers only containing blood at the ends of the feather shaft and no retained sheath covering the barbs.

**Internal:** Larger than typical but otherwise normal in appearance liver and kidneys. Prominent spleen. No obvious water in the airways. Proventriculus was empty. Ventriculus contained a piece of plastic, algae, squid beaks and cuttlefish. Mucosal lining of ventriculus had started to separate in places (likely post-mortem change.)

**SH-PI-22-16**

| **Collection Year** | | | 2022 | | | | | | | **Bird ID** | | | | SH-PI-22-16 | | | | | |
| --- | --- | --- | --- | --- | --- | --- | --- | --- | --- | --- | --- | --- | --- | --- | --- | --- | --- | --- | --- |
| **Freshness** | | | FFF (Very fresh) | | | | | | | **Completeness** | | | | CC | | | | | |
| **Measurements** | | Culmen length | | | | | | | 3.3 | | | Bill width | | | | | 0.6 | | |
| **(cm)** | | Bill depth | | | | | | | 0.8 | | | Head length | | | | | 8.2 | | |
|  | | Tarsometatarsus length | | | | | | | 5.0 | | | Radius/Ulna length | | | | | 10.5 | | |
|  | | Heart base-apex | | | | | | | 2.3 | | |  | | | | |  | | |
| **Body Condition** | Intestinal fat (0-3) | | | | 2 | Subcutaneous fat (0-3) | | | | | 1 | Breast muscle (0-3) | | | 1 | BCI (0-9) | | | **4** |
| **Gastrointestinal plastic?** | | Proventriculus | | | | | N | Ventriculus | | | | Y | **Bursa of Fabricius?** | | | | | Y | |
| **Complete GIT?** | | Y | | **If no, details:** | | | | | | | | | | | | | | | |

**Summary of abnormal/significant findings if present:**

**Scavenging:** Not scavenged.

**External:** Low-Moderate body condition. No obvious signs of trauma.

**Internal:** Higher grade of intestinal fat than subcutaneous fat present. Liver and kidneys are on the smaller side. No obvious fluid in airways. Proventriculus: empty. Ventriculus: Very little food, mostly plastic with a few small rocks.

**SH-PI-22-17**

| **Collection Year** | | | 2022 | | | | | | | **Bird ID** | | | | SH-PI-22-17 | | | | | |
| --- | --- | --- | --- | --- | --- | --- | --- | --- | --- | --- | --- | --- | --- | --- | --- | --- | --- | --- | --- |
| **Freshness** | | | FFF (Very fresh) | | | | | | | **Completeness** | | | | C | | | | | |
| **Measurements** | | Culmen length | | | | | | | 3.25 | | | Bill width | | | | | 0.7 | | |
| **(cm)** | | Bill depth | | | | | | | 0.8 | | | Head length | | | | | 7.5 | | |
|  | | Tarsometatarsus length | | | | | | | 4.9 | | | Radius/Ulna length | | | | | 9.4 | | |
|  | | Heart base-apex | | | | | | | 2.1 | | |  | | | | |  | | |
| **Body Condition** | Intestinal fat (0-3) | | | | 1 | Subcutaneous fat (0-3) | | | | | 0 | Breast muscle (0-3) | | | 0 | BCI (0-9) | | | **1** |
| **Gastrointestinal plastic?** | | Proventriculus | | | | | Y | Ventriculus | | | | Y | **Bursa of Fabricius?** | | | | | Y | |
| **Complete GIT?** | | Y | | **If no, details:** | | | | | | | | | | | | | | | |

**Summary of abnormal/significant findings if present:**

**Scavenging:** Damage to brain. Part of brain likely removed.

**External:** Emaciated. Some damage to skin with bleeding and bruising on head. Suspected predatory bird attack from appearance of wounds.

**Internal:** Multiple skull fractures (frontal, parietal) with opening of the cranial cavity. Brain was severely damaged, so was lacking normal structure/mushy and haemorrhagic. Liver and kidneys on the smaller side but otherwise normal in external appearance. No obvious fluid in airways. Proventriculus contained plastic (some large in size), green ingesta and two small sticks. Ventriculus contained plastic (some large in size), a disintegrating wooden fragment, a small rock, and a small amount of green ingesta.

**SH-PI-22-18**

| **Collection Year** | | | 2022 | | | | | | | **Bird ID** | | | | SH-PI-22-18 | | | | | |
| --- | --- | --- | --- | --- | --- | --- | --- | --- | --- | --- | --- | --- | --- | --- | --- | --- | --- | --- | --- |
| **Freshness** | | | FFF (Very fresh) | | | | | | | **Completeness** | | | | C | | | | | |
| **Measurements** | | Culmen length | | | | | | | 3.45 | | | Bill width | | | | | 0.7 | | |
| **(cm)** | | Bill depth | | | | | | | 0.85 | | | Head length | | | | | 8.2 | | |
|  | | Tarsometatarsus length | | | | | | | 4.8 | | | Radius/Ulna length | | | | | 9.5 | | |
|  | | Heart base-apex | | | | | | | 2.5 | | |  | | | | |  | | |
| **Body Condition** | Intestinal fat (0-3) | | | | 3 | Subcutaneous fat (0-3) | | | | | 3 | Breast muscle (0-3) | | | 3 | BCI (0-9) | | | **9** |
| **Gastrointestinal plastic?** | | Proventriculus | | | | | Y | Ventriculus | | | | Y | **Bursa of Fabricius?** | | | | | Y | |
| **Complete GIT?** | | Y | | **If no, details:** | | | | | | | | | | | | | | | |

**Summary of abnormal/significant findings if present:**

**Scavenging:** Mild scavenging of cranial left chest/pectoral muscle area with an opening into the coelomic cavity.

**External:** Bird appeared fat and well grown. No retained feather sheaths long enough to cover the barbs of the primary wing feathers.

**Internal:** Wet lungs with water also present in air sacs. Very little blood present in the heart chambers. Proventriculus contained plastic, feathers, a few squid beaks and lots of green ingesta. Ventriculus contained many plastic pieces, squid beaks, small stones, sand, cuttlefish bone and green ingesta.

**SH-PI-22-19**

| **Collection Year** | | | 2022 | | | | | | | **Bird ID** | | | | SH-PI-22-19 | | | | | |
| --- | --- | --- | --- | --- | --- | --- | --- | --- | --- | --- | --- | --- | --- | --- | --- | --- | --- | --- | --- |
| **Freshness** | | | FFF (Very fresh) | | | | | | | **Completeness** | | | | CC | | | | | |
| **Measurements** | | Culmen length | | | | | | | 3.3 | | | Bill width | | | | | 0.7 | | |
| **(cm)** | | Bill depth | | | | | | | 0.85 | | | Head length | | | | | 7.95 | | |
|  | | Tarsometatarsus length | | | | | | | 5.0 | | | Radius/Ulna length | | | | | 10.3 | | |
|  | | Heart base-apex | | | | | | | 2.5 | | |  | | | | |  | | |
| **Body Condition** | Intestinal fat (0-3) | | | | 2 | Subcutaneous fat (0-3) | | | | | 2 | Breast muscle (0-3) | | | 1 | BCI (0-9) | | | **5** |
| **Gastrointestinal plastic?** | | Proventriculus | | | | | N | Ventriculus | | | | Y | **Bursa of Fabricius?** | | | | | Y | |
| **Complete GIT?** | | Y | | **If no, details:** | | | | | | | | | | | | | | | |

**Summary of abnormal/significant findings if present:**

**Scavenging:** Not Scavenged.

**External:** Moderate body condition. Bruising on head and proximal right wing in the humerus region. Unbroken skin.

**Internal:** Fractured skull (frontal and parietal bones.) No obvious wing fractures- soft tissue trauma to the proximal right wing area. Water in lungs. Proventriculus was empty. Little ingesta in the intestines. A large, rounded piece of pumice (1cm x 1cm x 0.7cm diameter) took up most of the space inside the ventriculus and would likely have interfered with normal digestion/passage of food. Plastic was present in the ventriculus with a small amount of feather fragments and green ingesta. (The pumice would have been the major cause of the obstruction due to its size and shape even though plastic was also present.)


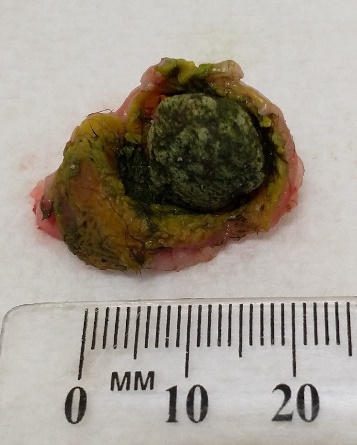


*Pumice in the ventriculus of SH-PI-22-19.*

**SH-PI-22-20**

| **Collection Year** | | | 2022 | | | | | | | **Bird ID** | | | | SH-PI-22-20 | | | | | |
| --- | --- | --- | --- | --- | --- | --- | --- | --- | --- | --- | --- | --- | --- | --- | --- | --- | --- | --- | --- |
| **Freshness** | | | FFF (Very fresh) | | | | | | | **Completeness** | | | | C | | | | | |
| **Measurements** | | Culmen length | | | | | | | 3.25 | | | Bill width | | | | | 0.7 | | |
| **(cm)** | | Bill depth | | | | | | | 0.9 | | | Head length | | | | | 7.9 | | |
|  | | Tarsometatarsus length | | | | | | | 5.0 | | | Radius/Ulna length | | | | | 10.5 | | |
|  | | Heart base-apex | | | | | | | 3.0 | | |  | | | | |  | | |
| **Body Condition** | Intestinal fat (0-3) | | | | 3 | Subcutaneous fat (0-3) | | | | | 3 | Breast muscle (0-3) | | | 3 | BCI (0-9) | | | **9** |
| **Gastrointestinal plastic?** | | Proventriculus | | | | | N | Ventriculus | | | | Y | **Bursa of Fabricius?** | | | | | Y | |
| **Complete GIT?** | | Y | | **If no, details:** | | | | | | | | | | | | | | | |

**Summary of abnormal/significant findings if present:**

**Scavenging:** Some removal of pectoral muscle on right and left side with opening into coelomic cavity in cranial right chest area.

**External:** Fairly large, well grown appearing bird. Fat body condition. Trauma/peck marks/bruising on head. Primary wing feathers almost mature with just a small amount of blood in the tips of the quills and no feather sheath covering the feather barbs. The second most distal primary wing feather was noticeably different in length with the right being 0.8cm longer than the left.

**Internal:** No obvious water in airways. Proventriculus has no plastic and contained a small amount of green ingesta with five small rocks. Ventriculus contained plastic and numerous small rocks, but little other ingesta.

**SH-PI-22-21**

| **Collection Year** | | | 2022 | | | | | | | **Bird ID** | | | | SH-PI-22-21 | | | | | |
| --- | --- | --- | --- | --- | --- | --- | --- | --- | --- | --- | --- | --- | --- | --- | --- | --- | --- | --- | --- |
| **Freshness** | | | FFF (Very fresh) | | | | | | | **Completeness** | | | | CC | | | | | |
| **Measurements** | | Culmen length | | | | | | | 3.0 | | | Bill width | | | | | 0.6 | | |
| **(cm)** | | Bill depth | | | | | | | 0.7 | | | Head length | | | | | 7.3 | | |
|  | | Tarsometatarsus length | | | | | | | 4.8 | | | Radius/Ulna length | | | | | 9.65 | | |
|  | | Heart base-apex | | | | | | | 2.3 | | |  | | | | |  | | |
| **Body Condition** | Intestinal fat (0-3) | | | | 0 | Subcutaneous fat (0-3) | | | | | 0 | Breast muscle (0-3) | | | 0 | BCI (0-9) | | | **0** |
| **Gastrointestinal plastic?** | | Proventriculus | | | | | Y | Ventriculus | | | | Y | **Bursa of Fabricius?** | | | | | Y | |
| **Complete GIT?** | | Y | | **If no, details:** | | | | | | | | | | | | | | | |

**Summary of abnormal/significant findings if present:**

**Scavenging:** Not scavenged.

**External:** Emaciated. Bruising around head.

**Internal:** Bones appear under-mineralised being more flexible than typical, especially the sternum which was rubbery and not straight. A small amount of water present in airways. Fractured skull (frontal and parietal bones.) Proventriculus contains plastic, feather fragments and mucous with a small number of squid beaks. Ventriculus contains plastic, squid beaks, feather fragments and a small amount of green ingesta.


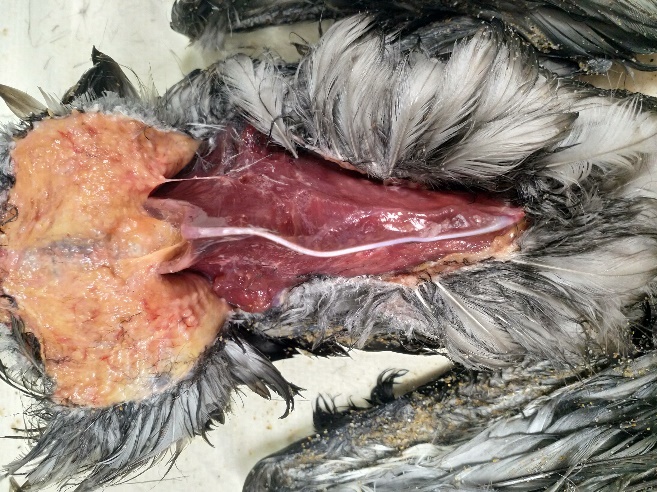


*Ventral chest area showing the flexible sternum of SH-PI-22-21.*

**SH-PI-22-22**

| **Collection Year** | | | 2022 | | | | | | | **Bird ID** | | | | SH-PI-22-22 | | | | | |
| --- | --- | --- | --- | --- | --- | --- | --- | --- | --- | --- | --- | --- | --- | --- | --- | --- | --- | --- | --- |
| **Freshness** | | | FFF (Very fresh) | | | | | | | **Completeness** | | | | I | | | | | |
| **Measurements** | | Culmen length | | | | | | | 3.3 | | | Bill width | | | | | 0.7 | | |
| **(cm)** | | Bill depth | | | | | | | 0.9 | | | Head length | | | | | 8.2 | | |
|  | | Tarsometatarsus length | | | | | | | 5.1 | | | Radius/Ulna length | | | | | 10.7 | | |
|  | | Heart base-apex | | | | | | | - | | |  | | | | |  | | |
| **Body Condition** | Intestinal fat (0-3) | | | | - | Subcutaneous fat (0-3) | | | | | 2 | Breast muscle (0-3) | | | 2 | BCI (0-9) | | | **-** |
| **Gastrointestinal plastic?** | | Proventriculus | | | | | NA | Ventriculus | | | | NA | **Bursa of Fabricius?** | | | | | Y | |
| **Complete GIT?** | | N | | **If no, details:** Missing proventriculus, ventriculus, most of small intestine. | | | | | | | | | | | | | | | |

**Summary of abnormal/significant findings if present:**

**Scavenging:** Heavily scavenged. Skull was fractured opening the cranial cavity with most of brain removed. Most of subcutaneous fat and pectoral muscle over right chest area removed. Some scavenging damage elsewhere to skin/muscle/fat on back and wings. Heart and right lung removed. Hole into the coelomic cavity in the right chest area. Cranial sections of both kidneys were missing. Gastrointestinal tract incomplete: mid-distal oesophagus, proventriculus, ventriculus and most of the small intestine removed. Liver and much of the abdominal fat is missing.

**External:** Complete fracture of the upper right femur. Degree of scavenging makes it difficult to be certain if this occurred before or after death.

**Internal:** Degree of damage and removal of multiple organs/body tissues during scavenging makes this bird difficult to assess. Remaining lung was heavy and wet that may indicate water aspiration, however there was also some free watery blood in the body cavity making it uncertain if predation was also a factor. No obvious sand contamination inside the coelomic cavity itself.

**SH-PI-22-23**

| **Collection Year** | | | 2022 | | | | | | | **Bird ID** | | | | SH-PI-22-23 | | | | | |
| --- | --- | --- | --- | --- | --- | --- | --- | --- | --- | --- | --- | --- | --- | --- | --- | --- | --- | --- | --- |
| **Freshness** | | | FFF (Very fresh) | | | | | | | **Completeness** | | | | CC | | | | | |
| **Measurements** | | Culmen length | | | | | | | 3.15 | | | Bill width | | | | | 0.65 | | |
| **(cm)** | | Bill depth | | | | | | | 0.9 | | | Head length | | | | | 7.7 | | |
|  | | Tarsometatarsus length | | | | | | | 5.0 | | | Radius/Ulna length | | | | | 9.4 | | |
|  | | Heart base-apex | | | | | | | 2.4 | | |  | | | | |  | | |
| **Body Condition** | Intestinal fat (0-3) | | | | 1 | Subcutaneous fat (0-3) | | | | | 1 | Breast muscle (0-3) | | | 0 | BCI (0-9) | | | **2** |
| **Gastrointestinal plastic?** | | Proventriculus | | | | | Y | Ventriculus | | | | Y | **Bursa of Fabricius?** | | | | | Y | |
| **Complete GIT?** | | Y | | **If no, details:** | | | | | | | | | | | | | | | |

**Summary of abnormal/significant findings if present:**

**Scavenging:** Not scavenged.

**External:** Very thin. Bruising present in proximal right wing over humerus area with peck like marks in skin. Broken neck with bruising.

**Internal:** No obvious water in airways. Liver on the smaller side but normal in gross appearance otherwise. Only a small amount of blood in the heart chambers. Minimal ingesta in the intestines. Proventriculus contained plastic (including a large piece), some wooden fragments and food ingesta present. The mucosa of the proximal proventriculus appears mildly irritated with increased erythema in the area. A small shallow ulcer is present in this area. Ventriculus contains plastic, 3 wooden fragments, small rocks and liquid ingesta.

**SH-PI-22-24**

| **Collection Year** | | | 2022 | | | | | | | **Bird ID** | | | | SH-PI-22-24 | | | | | |
| --- | --- | --- | --- | --- | --- | --- | --- | --- | --- | --- | --- | --- | --- | --- | --- | --- | --- | --- | --- |
| **Freshness** | | | FFF (Very fresh) | | | | | | | **Completeness** | | | | C | | | | | |
| **Measurements** | | Culmen length | | | | | | | 3.15 | | | Bill width | | | | | 0.75 | | |
| **(cm)** | | Bill depth | | | | | | | 0.9 | | | Head length | | | | | 8.1 | | |
|  | | Tarsometatarsus length | | | | | | | 5.0 | | | Radius/Ulna length | | | | | 9.95 | | |
|  | | Heart base-apex | | | | | | | 2.3 | | |  | | | | |  | | |
| **Body Condition** | Intestinal fat (0-3) | | | | 2 | Subcutaneous fat (0-3) | | | | | 1 | Breast muscle (0-3) | | | 1 | BCI (0-9) | | | **4** |
| **Gastrointestinal plastic?** | | Proventriculus | | | | | Y | Ventriculus | | | | N | **Bursa of Fabricius?** | | | | | Y | |
| **Complete GIT?** | | Y | | **If no, details:** | | | | | | | | | | | | | | | |

**Summary of abnormal/significant findings if present:**

**Scavenging:** Some scavenging of pectoral muscle on the left side of the chest with a hole opened into the coelomic cavity.

**External:** Externally appeared in underweight body condition. (Although score 2 intestinal fat present.)

**Internal:** Trauma to the left chest, some blood clots in muscle edges cranially. Liver on the smaller side but otherwise grossly normal in appearance. No obvious water in airways. Proventriculus contained many pieces of plastic and was otherwise empty apart from 2 squid beaks. Ingesta present in ventriculus without plastic.

**SH-PI-22-25**

| **Collection Year** | | | 2022 | | | | | | | **Bird ID** | | | | SH-PI-22-25 | | | | | |
| --- | --- | --- | --- | --- | --- | --- | --- | --- | --- | --- | --- | --- | --- | --- | --- | --- | --- | --- | --- |
| **Freshness** | | | FFF (Very fresh) | | | | | | | **Completeness** | | | | I | | | | | |
| **Measurements** | | Culmen length | | | | | | | 3.25 | | | Bill width | | | | | 0.7 | | |
| **(cm)** | | Bill depth | | | | | | | 0.9 | | | Head length | | | | | 8.0 | | |
|  | | Tarsometatarsus length | | | | | | | 5.1 | | | Radius/Ulna length | | | | | 9.8 | | |
|  | | Heart base-apex | | | | | | | 2.9 | | |  | | | | |  | | |
| **Body Condition** | Intestinal fat (0-3) | | | | 3 | Subcutaneous fat (0-3) | | | | | 3 | Breast muscle (0-3) | | | 3 | BCI (0-9) | | | **9** |
| **Gastrointestinal plastic?** | | Proventriculus | | | | | Y | Ventriculus | | | | Y | **Bursa of Fabricius?** | | | | | Y | |
| **Complete GIT?** | | Y | | **If no, details:** | | | | | | | | | | | | | | | |

**Summary of abnormal/significant findings if present:**

**Scavenging:** Significant scavenging of breast muscle and subcutaneous fat on the right chest with an opening into the coelomic cavity in this region. Damage to air sacs. Most of left lung and some of the liver had been removed.

**External:** Fat body condition. All primary and secondary wing feathers are very small blood feathers and enclosed in a feather sheath. Bird would have had difficulty flying in current state. Flight feathers on wings (primary and secondary) appear very abnormal compared to all other birds assessed. Feathers are a longer and more normal in appearance on tail. Powder down difficult to assess due to bird having been immersed in seawater. Beak and claws not obviously abnormal.

**Internal:** Large amount of free blood present in the coelomic cavity and evidence of haemorrhage associated with the damaged organs (both lungs, liver). Suggestive of significant bleeding while still alive due to injuries, likely including those occurring during predation. Prominent spleen. Plastic in both proventriculus and ventriculus (large number of pieces in ventriculus.) Large numbers of squid beaks in ventriculus.


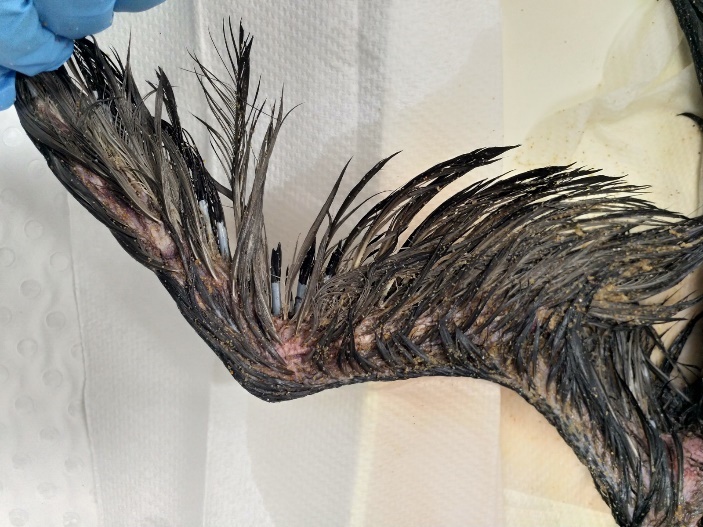


*Poorly developed flight feathers on the left wing (ventral view) of* *SH-PI-22-25. Small blood feathers largely encased in feather sheaths.*


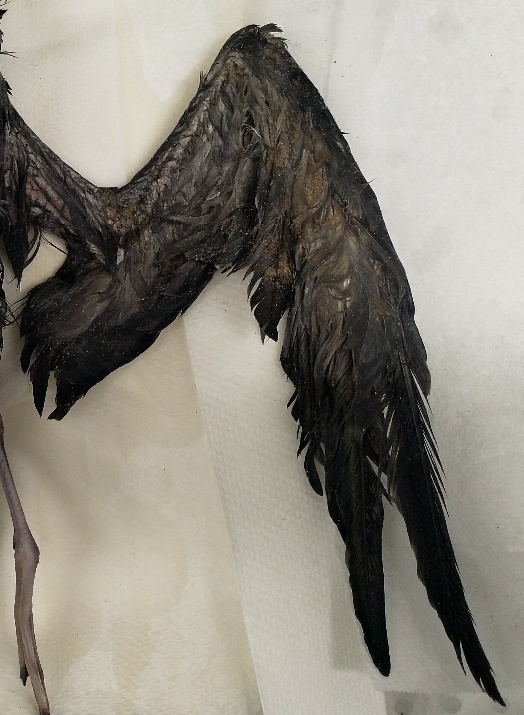


*Comparison photo of the ventral left wing from a different bird (SH-PI-22-26) showing an example of flight feathers which are more typical in appearance for shearwaters collected for this study.*

**SH-PI-22-26**

| **Collection Year** | | | 2022 | | | | | | | **Bird ID** | | | | SH-PI-22-26 | | | | | |
| --- | --- | --- | --- | --- | --- | --- | --- | --- | --- | --- | --- | --- | --- | --- | --- | --- | --- | --- | --- |
| **Freshness** | | | FFF (Very fresh) | | | | | | | **Completeness** | | | | CC | | | | | |
| **Measurements** | | Culmen length | | | | | | | 3.15 | | | Bill width | | | | | 0.8 | | |
| **(cm)** | | Bill depth | | | | | | | 0.85 | | | Head length | | | | | 7.9 | | |
|  | | Tarsometatarsus length | | | | | | | 5.1 | | | Radius/Ulna length | | | | | 10.6 | | |
|  | | Heart base-apex | | | | | | | 2.5 | | |  | | | | |  | | |
| **Body Condition** | Intestinal fat (0-3) | | | | 3 | Subcutaneous fat (0-3) | | | | | 3 | Breast muscle (0-3) | | | 2 | BCI (0-9) | | | **8** |
| **Gastrointestinal plastic?** | | Proventriculus | | | | | Y | Ventriculus | | | | Y | **Bursa of Fabricius?** | | | | | Y | |
| **Complete GIT?** | | Y | | **If no, details:** | | | | | | | | | | | | | | | |

**Summary of abnormal/significant findings if present:**

**Scavenging:** Not scavenged.

**External:** Fat body condition. No evidence of trauma.

**Internal:** No obvious water in airways. Liver on the smaller side but otherwise normal in appearance. Proventriculus distended with a large amount of green ingesta that also contained some feather fragments, oil droplets and plastic. Ventriculus contained plastic, a cuttlefish fragment, and large numbers of squid beaks. Minimal ingesta present in the small intestine. Could not identify an obvious foreign body obstruction present at the time of necropsy.

**SH-PI-22-27**

| **Collection Year** | | | 2022 | | | | | | | **Bird ID** | | | | SH-PI-22-27 | | | | | |
| --- | --- | --- | --- | --- | --- | --- | --- | --- | --- | --- | --- | --- | --- | --- | --- | --- | --- | --- | --- |
| **Freshness** | | | FFF (Very fresh) | | | | | | | **Completeness** | | | | CC | | | | | |
| **Measurements** | | Culmen length | | | | | | | 3.3 | | | Bill width | | | | | 0.7 | | |
| **(cm)** | | Bill depth | | | | | | | 0.8 | | | Head length | | | | | 7.9 | | |
|  | | Tarsometatarsus length | | | | | | | 5.1 | | | Radius/Ulna length | | | | | 9.6 | | |
|  | | Heart base-apex | | | | | | | 2.6 | | |  | | | | |  | | |
| **Body Condition** | Intestinal fat (0-3) | | | | 0 | Subcutaneous fat (0-3) | | | | | 0 | Breast muscle (0-3) | | | 0 | BCI (0-9) | | | **0** |
| **Gastrointestinal plastic?** | | Proventriculus | | | | | N | Ventriculus | | | | Y | **Bursa of Fabricius?** | | | | | Y | |
| **Complete GIT?** | | Y | | **If no, details:** | | | | | | | | | | | | | | | |

**Summary of abnormal/significant findings if present:**

**Scavenging:** Not scavenged.

**External:** Emaciated. No evidence of trauma.

**Internal:** Large amount of water in lungs, heavy and wet. Heart chambers distended with blood. Not a lot of ingesta in the gastrointestinal tract. Proventriculus was empty apart from a small piece of bark. Plastic and many squid beaks present in the ventriculus.

**SH-PI-22-28**

| **Collection Year** | | | 2022 | | | | | | | **Bird ID** | | | | SH-PI-22-28 | | | | | |
| --- | --- | --- | --- | --- | --- | --- | --- | --- | --- | --- | --- | --- | --- | --- | --- | --- | --- | --- | --- |
| **Freshness** | | | FFF (Very fresh) | | | | | | | **Completeness** | | | | C | | | | | |
| **Measurements** | | Culmen length | | | | | | | 3.2 | | | Bill width | | | | | 0.7 | | |
| **(cm)** | | Bill depth | | | | | | | 0.7 | | | Head length | | | | | 7.1 | | |
|  | | Tarsometatarsus length | | | | | | | 5.1 | | | Radius/Ulna length | | | | | 9.7 | | |
|  | | Heart base-apex | | | | | | | 2.2 | | |  | | | | |  | | |
| **Body Condition** | Intestinal fat (0-3) | | | | 1 | Subcutaneous fat (0-3) | | | | | 1 | Breast muscle (0-3) | | | 1 | BCI (0-9) | | | **3** |
| **Gastrointestinal plastic?** | | Proventriculus | | | | | N | Ventriculus | | | | Y | **Bursa of Fabricius?** | | | | | Y | |
| **Complete GIT?** | | Y | | **If no, details:** | | | | | | | | | | | | | | | |

**Summary of abnormal/significant findings if present:**

**Scavenging:** Part of brain removed.

**External:** Thin. Trauma/bruising to head.

**Internal:** Frontal bone fracture. Open cranial cavity with haemorrhage. Brain damaged due to scavenging. Liver on the smaller side but otherwise normal in appearance. No obvious water in airways. Only small amount of blood in the heart chambers. Proventriculus contained a large number of rocks with no plastic and little other ingesta. Ventriculus feels hard and impacted with large numbers of rocks, but also contained some plastic, cuttlefish pieces and other ingesta. Not a lot of ingesta in the intestine. Likely obstructive disease occurring in the ventriculus +/- proventriculus. No obvious ulcerations of gastrointestinal tract.


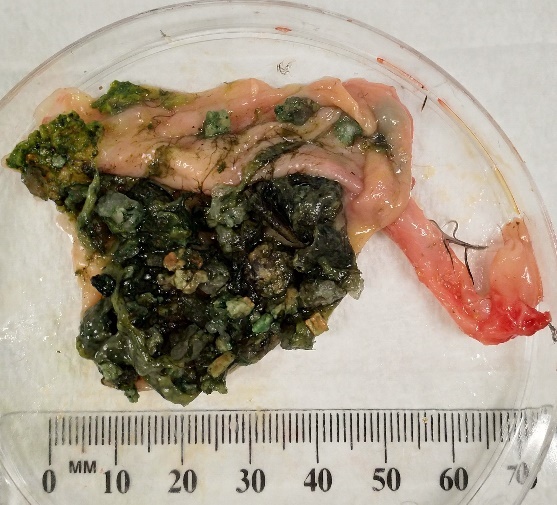


*Dissected proventriculus of SH-PI-22-28 containing large numbers of rocks.*

**SH-PI-22-29**

| **Collection Year** | | | 2022 | | | | | | | **Bird ID** | | | | SH-PI-22-29 | | | | | |
| --- | --- | --- | --- | --- | --- | --- | --- | --- | --- | --- | --- | --- | --- | --- | --- | --- | --- | --- | --- |
| **Freshness** | | | FFF (Very fresh) | | | | | | | **Completeness** | | | | I | | | | | |
| **Measurements** | | Culmen length | | | | | | | 3.4 | | | Bill width | | | | | 0.7 | | |
| **(cm)** | | Bill depth | | | | | | | 0.8 | | | Head length | | | | | - | | |
|  | | Tarsometatarsus length | | | | | | | 5.0 | | | Radius/Ulna length | | | | | 9.8 | | |
|  | | Heart base-apex | | | | | | | 2.7 | | |  | | | | |  | | |
| **Body Condition** | Intestinal fat (0-3) | | | | 2 | Subcutaneous fat (0-3) | | | | | 2 | Breast muscle (0-3) | | | 3 | BCI (0-9) | | | **7** |
| **Gastrointestinal plastic?** | | Proventriculus | | | | | N | Ventriculus | | | | N | **Bursa of Fabricius?** | | | | | Y | |
| **Complete GIT?** | | Y | | **If no, details:** | | | | | | | | | | | | | | | |

**Summary of abnormal/significant findings if present:**

**Scavenging:** Large amount of brain removed. Some pectoral muscle removed from left chest.

**External:** Good body condition. Bruising around head. Badly fractured skull involving parietal and frontal bones with open fractures into the cranial cavity.

**Internal:** No obvious water in airways. Liver is on the smaller side and a lighter tan in colour but not rounded in appearance. No ingested plastic noted. Feather pieces, three wooden fragments, squid beaks and liquid green ingesta present in proventriculus. Minimal ingesta in ventriculus.

**SH-PI-22-30**

| **Collection Year** | | | 2022 | | | | | | | **Bird ID** | | | | SH-PI-22-30 | | | | | |
| --- | --- | --- | --- | --- | --- | --- | --- | --- | --- | --- | --- | --- | --- | --- | --- | --- | --- | --- | --- |
| **Freshness** | | | FFF (Very fresh) | | | | | | | **Completeness** | | | | I | | | | | |
| **Measurements** | | Culmen length | | | | | | | 3.2 | | | Bill width | | | | | 0.65 | | |
| **(cm)** | | Bill depth | | | | | | | 0.8 | | | Head length | | | | | - | | |
|  | | Tarsometatarsus length | | | | | | | 5.1 | | | Radius/Ulna length | | | | | 9.7 | | |
|  | | Heart base-apex | | | | | | | 2.2 | | |  | | | | |  | | |
| **Body Condition** | Intestinal fat (0-3) | | | | 0 | Subcutaneous fat (0-3) | | | | | 0 | Breast muscle (0-3) | | | 0 | BCI (0-9) | | | **0** |
| **Gastrointestinal plastic?** | | Proventriculus | | | | | Y | Ventriculus | | | | Y | **Bursa of Fabricius?** | | | | | Y | |
| **Complete GIT?** | | Y | | **If no, details:** | | | | | | | | | | | | | | | |

**Summary of abnormal/significant findings if present:**

**Scavenging:** Most of brain removed.

**External:** Emaciated. Brusing on head with badly fractured skull involving frontal and parietal bones. Open fractures into cranial cavity.

**Internal:** No obvious water in airways. Heart and liver were on the smaller side. Colour was normal apart from bile staining of part of the liver which was in contact with the gall bladder. Gall bladder was very large and full of bile. Intestines were thin and contained little ingesta. Multiple large to very large pieces of plastic, a large piece of cuttlefish and a squid beak were present in the proventriculus. The proventriculus wall was almost translucent in places where the plastic was pushing against it due to their size, but no ulcerations identified. Only a small amount of other ingesta present was in the proventriculus. Ingesta containing plastic was present in the ventriculus.


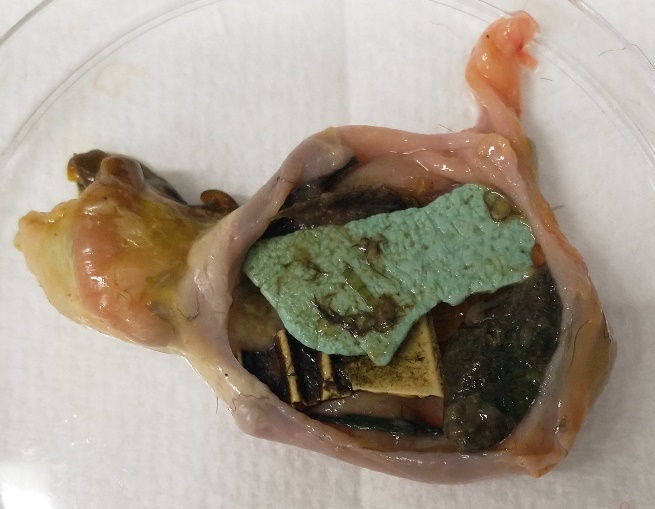

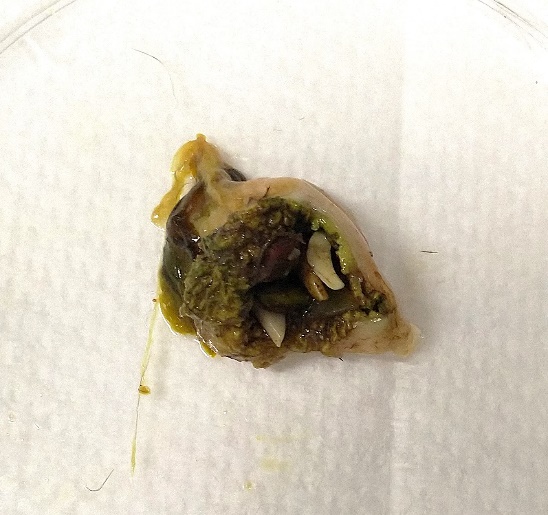


*Multiple pieces of plastic in the dissected proventriculus (left) and ventriculus (right) of SH-PI-22-30.*

**SH-PI-22-31**

| **Collection Year** | | | 2022 | | | | | | | **Bird ID** | | | | SH-PI-22-31 | | | | | |
| --- | --- | --- | --- | --- | --- | --- | --- | --- | --- | --- | --- | --- | --- | --- | --- | --- | --- | --- | --- |
| **Freshness** | | | FFF (Very fresh) | | | | | | | **Completeness** | | | | CC | | | | | |
| **Measurements** | | Culmen length | | | | | | | 3.7 | | | Bill width | | | | | 0.7 | | |
| **(cm)** | | Bill depth | | | | | | | 0.8 | | | Head length | | | | | 7.9 | | |
|  | | Tarsometatarsus length | | | | | | | 5.1 | | | Radius/Ulna length | | | | | 10.0 | | |
|  | | Heart base-apex | | | | | | | 2.5 | | |  | | | | |  | | |
| **Body Condition** | Intestinal fat (0-3) | | | | 1 | Subcutaneous fat (0-3) | | | | | 1 | Breast muscle (0-3) | | | 0 | BCI (0-9) | | | **2** |
| **Gastrointestinal plastic?** | | Proventriculus | | | | | Y | Ventriculus | | | | Y | **Bursa of Fabricius?** | | | | | Y | |
| **Complete GIT?** | | Y | | **If no, details:** | | | | | | | | | | | | | | | |

**Summary of abnormal/significant findings if present:**

**Scavenging:** Not Scavenged.

**External:** Thin.

**Internal:** Lungs wet, water in air sacs. Liver on the smaller side but otherwise normal in appearance. Prominent spleen. Green ingesta and plastic in proventriculus and ventriculus.

**SH-PI-22-32**

| **Collection Year** | | | 2022 | | | | | | | **Bird ID** | | | | SH-PI-22-32 | | | | | |
| --- | --- | --- | --- | --- | --- | --- | --- | --- | --- | --- | --- | --- | --- | --- | --- | --- | --- | --- | --- |
| **Freshness** | | | FFF (Very fresh) | | | | | | | **Completeness** | | | | C | | | | | |
| **Measurements** | | Culmen length | | | | | | | 3.5 | | | Bill width | | | | | 0.7 | | |
| **(cm)** | | Bill depth | | | | | | | 0.85 | | | Head length | | | | | 7.8 | | |
|  | | Tarsometatarsus length | | | | | | | 4.95 | | | Radius/Ulna length | | | | | 9.5 | | |
|  | | Heart base-apex | | | | | | | 2.4 | | |  | | | | |  | | |
| **Body Condition** | Intestinal fat (0-3) | | | | 0 | Subcutaneous fat (0-3) | | | | | 1 | Breast muscle (0-3) | | | 2 | BCI (0-9) | | | **3** |
| **Gastrointestinal plastic?** | | Proventriculus | | | | | Y | Ventriculus | | | | Y | **Bursa of Fabricius?** | | | | | Y | |
| **Complete GIT?** | | Y | | **If no, details:** | | | | | | | | | | | | | | | |

**Summary of abnormal/significant findings if present:**

**Scavenging:** Minimal removal of soft tissues (skin/muscle) on head.

**External:** Thin. Wounds resembling peck wounds with damage to skin and underlying tissues on both sides of the head with bruising.

**Internal:** Two fractures of the skull involving the frontal and parietal bones. Haemorrhages present in the brain. Liver on the smaller side but normal in appearance otherwise. Multiple pieces of plastic, many large to very large present in the proventriculus. One piece was adhered to the mucosal wall with fibrinous attachments and has formed a deep ulcer associated with a pointed area of the plastic. (Not perforated.) Inflammation with increased blood supply (erythema of tissues and prominent, dilated blood vessels) was present associated with this area. Otherwise the proventriculus contained a small amount of mucous and green ingesta with feather and wood fragments. Ventriculus contains plastic and a small number of squid beaks and cuttlefish. Although this bird had reasonable breast muscle (2/3), fat stores were low (0/3 intestinal fat, 1/3 subcutaneous fat).


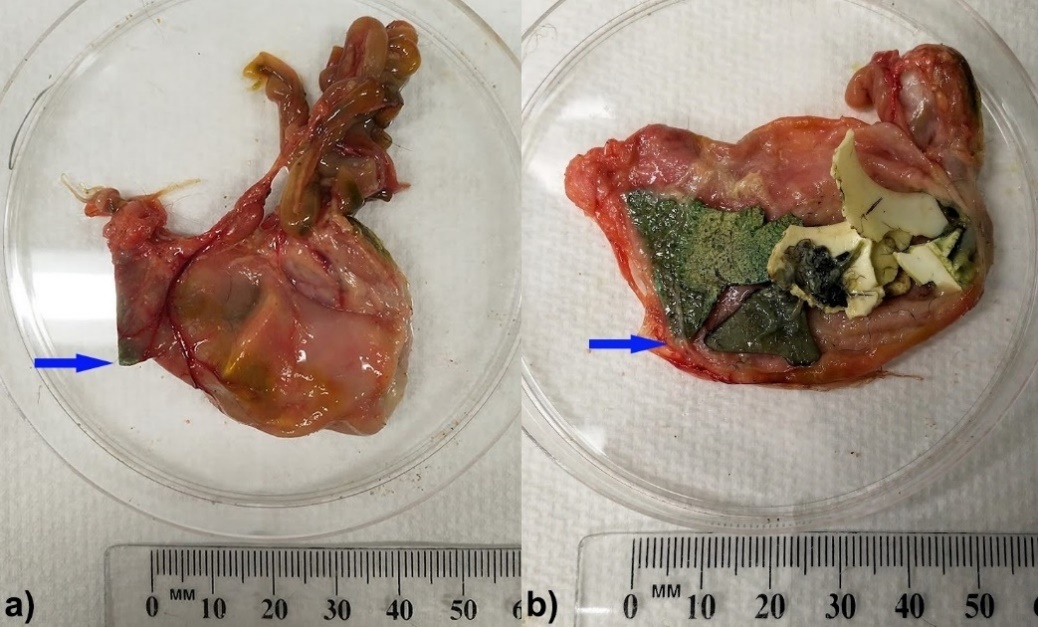


*External (a) and internal/dissected (b) views indicating a plastic fragment protruding into the proventriculus wall ulcerating the mucosal surface (arrows). Adhesions on the mucosal surface to the plastic, and inflammation with increased blood supply (erythema of tissues and prominent, dilated blood vessels) were present in this area. Proventriculus is from bird SH-PI-22-32.*

**SH-PI-22-33**

| **Collection Year** | | | 2022 | | | | | | | **Bird ID** | | | | SH-PI-22-33 | | | | | |
| --- | --- | --- | --- | --- | --- | --- | --- | --- | --- | --- | --- | --- | --- | --- | --- | --- | --- | --- | --- |
| **Freshness** | | | FFF (Very fresh) | | | | | | | **Completeness** | | | | I | | | | | |
| **Measurements** | | Culmen length | | | | | | | 3.4 | | | Bill width | | | | | 0.85 | | |
| **(cm)** | | Bill depth | | | | | | | 0.9 | | | Head length | | | | | 8.25 | | |
|  | | Tarsometatarsus length | | | | | | | 5.3 | | | Radius/Ulna length | | | | | 10.3 | | |
|  | | Heart base-apex | | | | | | | - | | |  | | | | |  | | |
| **Body Condition** | Intestinal fat (0-3) | | | | 3 | Subcutaneous fat (0-3) | | | | | 3 | Breast muscle (0-3) | | | 3 | BCI (0-9) | | | **9** |
| **Gastrointestinal plastic?** | | Proventriculus | | | | | - | Ventriculus | | | | - | **Bursa of Fabricius?** | | | | | Y | |
| **Complete GIT?** | | N | | **If no, details:** No proventriculus and ventriculus. | | | | | | | | | | | | | | | |

**Summary of abnormal/significant findings if present:**

**Scavenging:** Most of brain and right lung removed. Most of left breast muscle removed with an opening into the coelomic cavity in left chest area. Heart, proventriculus and ventriculus missing.

**External:** Fat body condition. Well grown looking bird with long, relatively mature looking primary/secondary flight feathers. (No retained feather sheath covering barbs). Skull (frontal bone) had been fractured with open fractures into the cranial cavity. Uncertain if occurred pre or postmortem.

**Internal:** No obvious water in the remaining airways however some damage had occurred during scavenging, particularly to air sacs and the right lung was missing so this could not be completely assessed. Most of brain removed, and the structure of the remaining brain tissue was damaged and mushy. Liver large in size, but otherwise normal in appearance. No plastic analysis due to missing proventriculus and ventriculus.

**SH-PI-22-34**

| **Collection Year** | | | 2022 | | | | | | | **Bird ID** | | | | SH-PI-22-34 | | | | | |
| --- | --- | --- | --- | --- | --- | --- | --- | --- | --- | --- | --- | --- | --- | --- | --- | --- | --- | --- | --- |
| **Freshness** | | | FFF (Very fresh) | | | | | | | **Completeness** | | | | CC | | | | | |
| **Measurements** | | Culmen length | | | | | | | 3.25 | | | Bill width | | | | | 0.8 | | |
| **(cm)** | | Bill depth | | | | | | | 0.8 | | | Head length | | | | | 7.65 | | |
|  | | Tarsometatarsus length | | | | | | | 5.1 | | | Radius/Ulna length | | | | | 9.6 | | |
|  | | Heart base-apex | | | | | | | 2.9 | | |  | | | | |  | | |
| **Body Condition** | Intestinal fat (0-3) | | | | 2 | Subcutaneous fat (0-3) | | | | | 3 | Breast muscle (0-3) | | | 2 | BCI (0-9) | | | **7** |
| **Gastrointestinal plastic?** | | Proventriculus | | | | | Y | Ventriculus | | | | Y | **Bursa of Fabricius?** | | | | | Y | |
| **Complete GIT?** | | Y | | **If no, details:** | | | | | | | | | | | | | | | |

**Summary of abnormal/significant findings if present:**

**Scavenging:** Not scavenged.

**External:** Good body condition. No obvious signs of trauma.

**Internal:** No obvious water in airways. Likely livor mortis in the right lung. Liver on the smaller side but appearance otherwise normal. Moderate amounts of ingesta in the intestines. Proventriculus contained plastic, many feather fragments, some mucous and a large amount of liquid green ingesta. Ventriculus contained plastic, feather fragments, a piece of cuttlefish, and green ingesta.

**SH-PI-22-35**

| **Collection Year** | | | 2022 | | | | | | | **Bird ID** | | | | SH-PI-22-36 | | | | | |
| --- | --- | --- | --- | --- | --- | --- | --- | --- | --- | --- | --- | --- | --- | --- | --- | --- | --- | --- | --- |
| **Freshness** | | | FFF (Very fresh) | | | | | | | **Completeness** | | | | C | | | | | |
| **Measurements** | | Culmen length | | | | | | | 2.7 | | | Bill width | | | | | 0.7 | | |
| **(cm)** | | Bill depth | | | | | | | 0.8 | | | Head length | | | | | 6.75 | | |
|  | | Tarsometatarsus length | | | | | | | 4.1 | | | Radius/Ulna length | | | | | 8.2 | | |
|  | | Heart base-apex | | | | | | | 2.4 | | |  | | | | |  | | |
| **Body Condition** | Intestinal fat (0-3) | | | | 3 | Subcutaneous fat (0-3) | | | | | 2 | Breast muscle (0-3) | | | 2 | BCI (0-9) | | | **7** |
| **Gastrointestinal plastic?** | | Proventriculus | | | | | Y | Ventriculus | | | | Y | **Bursa of Fabricius?** | | | | | Y | |
| **Complete GIT?** | | Y | | **If no, details:** | | | | | | | | | | | | | | | |

**Summary of abnormal/significant findings if present:**

**Scavenging:** Removal of much of the right pectoral muscle and subcutaneous fat in the right chest. Hole present into the coelomic cavity in the right chest area.

**External:** Good body condition. Small bird. Wounds, some resembling peck marks on the head with bruising.

**Internal:** No obvious water in airways. Very small liver for size, otherwise normal in appearance. Very large amount of green ingesta in the proventriculus containing squid beaks, feather pieces, bark and plastic. A piece of cuttlefish appeared to be lodged in the distal proventriculus/isthmus area likely causing obstructive disease. No ulcerations were noted. Plastic and only a small amount of ingesta were present in the ventriculus. Moderate amounts of ingesta were present in the intestines.

**SH-PI-22-36**

| **Collection Year** | | | 2022 | | | | | | | **Bird ID** | | | | SH-PI-22-36 | | | | | |
| --- | --- | --- | --- | --- | --- | --- | --- | --- | --- | --- | --- | --- | --- | --- | --- | --- | --- | --- | --- |
| **Freshness** | | | FFF (Very fresh) | | | | | | | **Completeness** | | | | C | | | | | |
| **Measurements** | | Culmen length | | | | | | | 3.15 | | | Bill width | | | | | 0.6 | | |
| **(cm)** | | Bill depth | | | | | | | 0.8 | | | Head length | | | | | - | | |
|  | | Tarsometatarsus length | | | | | | | 4.8 | | | Radius/Ulna length | | | | | 9.1 | | |
|  | | Heart base-apex | | | | | | | 2.4 | | |  | | | | |  | | |
| **Body Condition** | Intestinal fat (0-3) | | | | 1 | Subcutaneous fat (0-3) | | | | | 1 | Breast muscle (0-3) | | | 1 | BCI (0-9) | | | **3** |
| **Gastrointestinal plastic?** | | Proventriculus | | | | | Y | Ventriculus | | | | Y | **Bursa of Fabricius?** | | | | | Y | |
| **Complete GIT?** | | Y | | **If no, details:** | | | | | | | | | | | | | | | |

**Summary of abnormal/significant findings if present:**

**Scavenging:** Very minor removal of soft tissues around head (skin/muscle). Seen attacked by a Pacific Gull and likely died from head injuries sustained.

**External:** Very thin. Small bird. Bleeding, bruising and trauma around head. Blood present in nostrils

**Internal:** Badly fractured skull involving frontal, parietal and occipital bones. Bone fragments have been pushed into the cranial cavity and caused extensive bleeding and damage to the structure to the brain. No obvious water, but blood was present in the lungs and trachea. Likely aspirated from the bleeding on the head. Liver on the smaller side but otherwise normal in appearance. Large pieces of plastic and green ingesta were present in the proventriculus. Ventriculus contained plastic, rocks and green ingesta.

**SH-PI-22-37**

| **Collection Year** | | | 2022 | | | | | | | **Bird ID** | | | | SH-PI-22-37 | | | | | |
| --- | --- | --- | --- | --- | --- | --- | --- | --- | --- | --- | --- | --- | --- | --- | --- | --- | --- | --- | --- |
| **Freshness** | | | FFF (Very fresh) | | | | | | | **Completeness** | | | | CC | | | | | |
| **Measurements** | | Culmen length | | | | | | | 3.15 | | | Bill width | | | | | 0.7 | | |
| **(cm)** | | Bill depth | | | | | | | 0.9 | | | Head length | | | | | 7.7 | | |
|  | | Tarsometatarsus length | | | | | | | 4.5 | | | Radius/Ulna length | | | | | 8.8 | | |
|  | | Heart base-apex | | | | | | | 2.4 | | |  | | | | |  | | |
| **Body Condition** | Intestinal fat (0-3) | | | | 1 | Subcutaneous fat (0-3) | | | | | 1 | Breast muscle (0-3) | | | 2 | BCI (0-9) | | | **4** |
| **Gastrointestinal plastic?** | | Proventriculus | | | | | Y | Ventriculus | | | | Y | **Bursa of Fabricius?** | | | | | Y | |
| **Complete GIT?** | | Y | | **If no, details:** | | | | | | | | | | | | | | | |

**Summary of abnormal/significant findings if present:**

**Scavenging:** Not scavenged.

**External:** Low-moderate body condition. Trauma to the back of the head resembling peck marks with bruising, without fracturing the skull. Old poorly healed fracture of the left distal tibiotarsus close to the tibiotarsal-tarsometatarsal joint. Still has movement in the joint, however leg has healed deviated laterally and rotated which would make it impossible to stand on this leg normally. A pressure sore was developing on the left leg in the lower, caudal/distal tibiotarsus area.

**Internal:** No obvious water in the airways. Large amount of blood in the heart chambers. Proventriculus contained plastic, a few rocks and a small amount of mucous. Ventriculus contained plastic (some large) and quite a few rocks with little other ingesta.


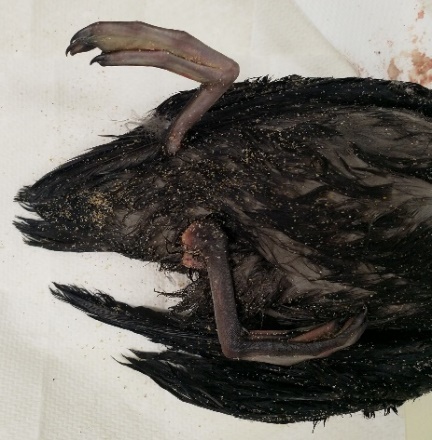


*Poorly healed left leg fracture of SH-PI-22-37.*

**SH-PI-22-38**

| **Collection Year** | | | 2022 | | | | | | | **Bird ID** | | | | SH-PI-22-38 | | | | | |
| --- | --- | --- | --- | --- | --- | --- | --- | --- | --- | --- | --- | --- | --- | --- | --- | --- | --- | --- | --- |
| **Freshness** | | | FF (Fesh) | | | | | | | **Completeness** | | | | I | | | | | |
| **Measurements** | | Culmen length | | | | | | | 3.0 | | | Bill width | | | | | 0.8 | | |
| **(cm)** | | Bill depth | | | | | | | 0.8 | | | Head length | | | | | 7.6 | | |
|  | | Tarsometatarsus length | | | | | | | 4.8 | | | Radius/Ulna length | | | | | 9.0 | | |
|  | | Heart base-apex | | | | | | | 2.1 | | |  | | | | |  | | |
| **Body Condition** | Intestinal fat (0-3) | | | | 3 | Subcutaneous fat (0-3) | | | | | 3 | Breast muscle (0-3) | | | 3 | BCI (0-9) | | | **9** |
| **Gastrointestinal plastic?** | | Proventriculus | | | | | - | Ventriculus | | | | - | **Bursa of Fabricius?** | | | | | Y | |
| **Complete GIT?** | | N | | **If no, details:** Proventriculus, ventriculus and part of intestines missing. | | | | | | | | | | | | | | | |

**Summary of abnormal/significant findings if present:**

**Scavenging:** Extensive scavenging around the chest and neck removing quite a bit of muscle in this area. Chest area had been opened and the proventriculus and ventriculus removed. Parts of the intestine had been removed with broken pieces remaining and all of the terminal small intestine to cloaca left in situ.

**External:** Fat body condition. Most flight feathers still have some blood in the shaft but otherwise look relatively mature apart from the most distal feather on each wing which still have a feather sheath covering barbs.

**Internal:** No obvious water in the lungs. Some ingesta and sand contamination in the coelomic cavity. Kidneys and liver appear mildly autolysed. No plastic analysis due to scavenging of the proventriculus and ventriculus.

**SH-PI-22-39**

| **Collection Year** | | | 2022 | | | | | | | **Bird ID** | | | | SH-PI-22-39 | | | | | |
| --- | --- | --- | --- | --- | --- | --- | --- | --- | --- | --- | --- | --- | --- | --- | --- | --- | --- | --- | --- |
| **Freshness** | | | FFF (Very fresh) | | | | | | | **Completeness** | | | | CC | | | | | |
| **Measurements** | | Culmen length | | | | | | | 3.4 | | | Bill width | | | | | 0.7 | | |
| **(cm)** | | Bill depth | | | | | | | 0.8 | | | Head length | | | | | 8.3 | | |
|  | | Tarsometatarsus length | | | | | | | 5.6 | | | Radius/Ulna length | | | | | 10.9 | | |
|  | | Heart base-apex | | | | | | | 3.2 | | |  | | | | |  | | |
| **Body Condition** | Intestinal fat (0-3) | | | | 3 | Subcutaneous fat (0-3) | | | | | 3 | Breast muscle (0-3) | | | 3 | BCI (0-9) | | | **9** |
| **Gastrointestinal plastic?** | | Proventriculus | | | | | N | Ventriculus | | | | Y | **Bursa of Fabricius?** | | | | | Y | |
| **Complete GIT?** | | Y | | **If no, details:** | | | | | | | | | | | | | | | |

**Summary of abnormal/significant findings if present:**

**Scavenging:** Not scavenged.

**External:** Large, fat, well grown appearing bird. Relatively mature appearing flight feathers. Blood in the nostrils. Point of haemorrhage and bruising over the right breast area (skin, subcutaneous, muscle). No apparent injuries most typical of gull attacks seen. Bird was found high on the beach and dry.

**Internal:** Blood was present in both lungs, air sacs and free in the coelomic cavity. Blood in nostrils possibly came from the lower airways as no obvious head trauma was identified. Proventriculus empty. Ventriculus contained plastic, squid beaks and a small amount of other ingesta.

**SH-PI-22-40**

| **Collection Year** | | | 2022 | | | | | | | **Bird ID** | | | | SH-PI-22-40 | | | | | |
| --- | --- | --- | --- | --- | --- | --- | --- | --- | --- | --- | --- | --- | --- | --- | --- | --- | --- | --- | --- |
| **Freshness** | | | FFF (Very fresh) | | | | | | | **Completeness** | | | | I | | | | | |
| **Measurements** | | Culmen length | | | | | | | 3.4 | | | Bill width | | | | | 0.7 | | |
| **(cm)** | | Bill depth | | | | | | | 0.7 | | | Head length | | | | | 7.6 | | |
|  | | Tarsometatarsus length | | | | | | | 4.8 | | | Radius/Ulna length | | | | | 9.0 | | |
|  | | Heart base-apex | | | | | | | - | | |  | | | | |  | | |
| **Body Condition** | Intestinal fat (0-3) | | | | 1 | Subcutaneous fat (0-3) | | | | | 1 | Breast muscle (0-3) | | | 1 | BCI (0-9) | | | **3** |
| **Gastrointestinal plastic?** | | Proventriculus | | | | | - | Ventriculus | | | | - | **Bursa of Fabricius?** | | | | | Y | |
| **Complete GIT?** | | N | | **If no, details:** Proventriculus and ventriculus missing. | | | | | | | | | | | | | | | |

**Summary of abnormal/significant findings if present:**

**Scavenging:** Significant amounts of scavenging with mainly muscle removed from the left back and part of the left chest with a hole made into the coelomic cavity in the left chest area. Missing proventriculus, ventriculus, liver and heart. Located on shoreline.

**External:** Very thin.

**Internal:** Difficult to assess due to degree of scavenging damage. Lungs and coelomic cavity contain water however it cannot be certain in this case if this occurred pre or postmortem. No plastic analysis due to scavenging of proventriculus and ventriculus.

**SH-PI-22-41**

| **Collection Year** | | | 2022 | | | | | | | **Bird ID** | | | | SH-PI-22-41 | | | | | |
| --- | --- | --- | --- | --- | --- | --- | --- | --- | --- | --- | --- | --- | --- | --- | --- | --- | --- | --- | --- |
| **Freshness** | | | FFF (Very fresh) | | | | | | | **Completeness** | | | | C | | | | | |
| **Measurements** | | Culmen length | | | | | | | 3.4 | | | Bill width | | | | | 0.8 | | |
| **(cm)** | | Bill depth | | | | | | | 0.9 | | | Head length | | | | | 8.0 | | |
|  | | Tarsometatarsus length | | | | | | | 5.15 | | | Radius/Ulna length | | | | | 10.25 | | |
|  | | Heart base-apex | | | | | | | 2.7 | | |  | | | | |  | | |
| **Body Condition** | Intestinal fat (0-3) | | | | 1 | Subcutaneous fat (0-3) | | | | | 0 | Breast muscle (0-3) | | | 1 | BCI (0-9) | | | **2** |
| **Gastrointestinal plastic?** | | Proventriculus | | | | | N | Ventriculus | | | | Y | **Bursa of Fabricius?** | | | | | Y | |
| **Complete GIT?** | | Y | | **If no, details:** | | | | | | | | | | | | | | | |

**Summary of abnormal/significant findings if present:**

**Scavenging:** Some pectoral muscle removed from left chest area with a hole made into the coelomic cavity.

**External:** Very thin. Bruising/peck marks on the caudal head.

**Internal:** Blood present in trachea, watery blood present in lungs. Proventriculus was empty. Ventriculus contained green ingesta with plastic and squid beaks.

**SH-PI-22-42**

| **Collection Year** | | | 2022 | | | | | | | **Bird ID** | | | | SH-PI-22-42 | | | | | |
| --- | --- | --- | --- | --- | --- | --- | --- | --- | --- | --- | --- | --- | --- | --- | --- | --- | --- | --- | --- |
| **Freshness** | | | FFF (Very fresh) | | | | | | | **Completeness** | | | | I | | | | | |
| **Measurements** | | Culmen length | | | | | | | 3.2 | | | Bill width | | | | | 0.7 | | |
| **(cm)** | | Bill depth | | | | | | | 0.8 | | | Head length | | | | | 7.9 | | |
|  | | Tarsometatarsus length | | | | | | | 5.2 | | | Radius/Ulna length | | | | | 10.3 | | |
|  | | Heart base-apex | | | | | | | 2.4 | | |  | | | | |  | | |
| **Body Condition** | Intestinal fat (0-3) | | | | 1 | Subcutaneous fat (0-3) | | | | | 1 | Breast muscle (0-3) | | | 1 | BCI (0-9) | | | **3** |
| **Gastrointestinal plastic?** | | Proventriculus | | | | | Y | Ventriculus | | | | Y | **Bursa of Fabricius?** | | | | | Y | |
| **Complete GIT?** | | Y | | **If no, details:** | | | | | | | | | | | | | | | |

**Summary of abnormal/significant findings if present:**

**Scavenging:** Most of brain removed.

**External:** Thin. Fractured skull (frontal bones) with bruising.

**Internal:** No obvious water in airways. Right kidney was larger (than the left) and had an uneven, slightly lumpy surface. It was not discoloured. Proventriculus contained large pieces of plastic, with a smaller amount of green ingesta containing feather and vegetation fragments. The mucosal lining of the proventriculus was not ulcerated but appears inflamed as the colour was a deeper pink to red in appearance compared to normal. The ventriculus contained plastic, a large piece of cuttlefish, squid beaks and a small amount of sand and other ingesta.

**SH-PI-22-43**

| **Collection Year** | | | 2022 | | | | | | | **Bird ID** | | | | SH-PI-22-43 | | | | | |
| --- | --- | --- | --- | --- | --- | --- | --- | --- | --- | --- | --- | --- | --- | --- | --- | --- | --- | --- | --- |
| **Freshness** | | | FF (Fresh) | | | | | | | **Completeness** | | | | I | | | | | |
| **Measurements** | | Culmen length | | | | | | | - | | | Bill width | | | | | - | | |
| **(cm)** | | Bill depth | | | | | | | - | | | Head length | | | | | - | | |
|  | | Tarsometatarsus length | | | | | | | 4.8 | | | Radius/Ulna length | | | | | 9.8 | | |
|  | | Heart base-apex | | | | | | | 2.6 | | |  | | | | |  | | |
| **Body Condition** | Intestinal fat (0-3) | | | | 1 | Subcutaneous fat (0-3) | | | | | 0 | Breast muscle (0-3) | | | 1 | BCI (0-9) | | | **2** |
| **Gastrointestinal plastic?** | | Proventriculus | | | | | N | Ventriculus | | | | Y | **Bursa of Fabricius?** | | | | | Y | |
| **Complete GIT?** | | Y | | **If no, details:** | | | | | | | | | | | | | | | |

**Summary of abnormal/significant findings if present:**

**Scavenging:** The dorsal head and top beak are missing, with removal of the brain and part of the tongue. Muscle on neck partially scavenged with an opening into the body cavity via thoracic inlet area.

**External:** Very thin.

**Internal:** Lungs contained water. Liver is small and gall bladder empty, normal appearance otherwise. Although the other internal organs look grossly ok, kidneys are showing signs of autolysis and were quite friable on removal during the necropsy. Little ingesta in the intestines. Proventriculus was empty. Ventriculus contained many pieces of plastic and some green ingesta with a few squid beaks.

**B) Necropsy reports for the year 2021.**

**ST-Wol-1**

| **Collection Year** | | | 2021 | | | | | | **Bird ID** | | | | ST-Wol-1 | | | | |
| --- | --- | --- | --- | --- | --- | --- | --- | --- | --- | --- | --- | --- | --- | --- | --- | --- | --- |
| **Freshness** | | | FF (Fresh) | | | | | | **Completeness** | | | | C | | | | |
| **Body Condition** | Intestinal fat (0-3) | | | | 0 | Subcutaneous fat (0-3) | | | | 0 | Breast muscle (0-3) | | | 0 | BCI (0-9) | | **0** |
| **Gastrointestinal plastic?** | | Proventriculus | | | | | N | Ventriculus | | | N | **Bursa of Fabricius?** | | | | Y | |
| **Complete GIT?** | | Y | | **If no, details:** | | | | | | | | | | | | | |

**Summary of abnormal/significant findings if present:**

**Scavenging:** Not scavenged.

**External:** Emaciated. Very small bird, looks immature. Chronic wound in the right lower neck area. Maggots were present in the wound. The bird was present on the upper beach close to the edge of the sand dunes and was dry.

**Internal:** Maggots were present in the right upper pectoral muscle and interclavicular air sac. No water observed in airways. Kidneys were large and pale with multiple small dark spots present on the right kidney. Some autolysis of kidneys appears to be present. Empty proventriculus and ventriculus. Some ingesta present in the intestines.

**ST-Wol-2**

| **Collection Year** | | | 2021 | | | | | | **Bird ID** | | | | ST-Wol-2 | | | | |
| --- | --- | --- | --- | --- | --- | --- | --- | --- | --- | --- | --- | --- | --- | --- | --- | --- | --- |
| **Freshness** | | | FFF (Very fresh) | | | | | | **Completeness** | | | | CC | | | | |
| **Body Condition** | Intestinal fat (0-3) | | | | 2 | Subcutaneous fat (0-3) | | | | 2 | Breast muscle (0-3) | | | 2 | BCI (0-9) | | **6** |
| **Gastrointestinal plastic?** | | Proventriculus | | | | | N | Ventriculus | | | Y | **Bursa of Fabricius?** | | | | Y | |
| **Complete GIT?** | | Y | | **If no, details:** | | | | | | | | | | | | | |

**Summary of abnormal/significant findings if present:**

**Scavenging:** Not scavenged.

**External:** No obvious trauma. High-moderate body condition.

**Internal:** No obvious water in airways. Proventriculus contains some ingesta without plastic. Ventriculus contains little food, mostly plastic with a few rocks.

**ST-Wol-3**

| **Collection Year** | | | 2021 | | | | | | **Bird ID** | | | | ST-Wol-3 | | | | |
| --- | --- | --- | --- | --- | --- | --- | --- | --- | --- | --- | --- | --- | --- | --- | --- | --- | --- |
| **Freshness** | | | FFF (Very fresh) | | | | | | **Completeness** | | | | CC | | | | |
| **Body Condition** | Intestinal fat (0-3) | | | | 3 | Subcutaneous fat (0-3) | | | | 3 | Breast muscle (0-3) | | | 3 | BCI (0-9) | | **9** |
| **Gastrointestinal plastic?** | | Proventriculus | | | | | N | Ventriculus | | | Y | **Bursa of Fabricius?** | | | | Y | |
| **Complete GIT?** | | Y | | **If no, details:** | | | | | | | | | | | | | |

**Summary of abnormal/significant findings if present:**

**Scavenging:** Not scavenged.

**External:** Very fat body condition. No obvious signs of trauma.

**Internal:** No obvious water in airways. Ingesta present in proventriculus without plastic. Small amount of food present in the proventriculus with small amount of plastic, one rock and a squid beak.

**ST-Wol-4**

| **Collection Year** | | | 2021 | | | | | | **Bird ID** | | | | ST-Wol-4 | | | | |
| --- | --- | --- | --- | --- | --- | --- | --- | --- | --- | --- | --- | --- | --- | --- | --- | --- | --- |
| **Freshness** | | | FFF (Very fresh) | | | | | | **Completeness** | | | | CC | | | | |
| **Body Condition** | Intestinal fat (0-3) | | | | 1 | Subcutaneous fat (0-3) | | | | 1 | Breast muscle (0-3) | | | 1 | BCI (0-9) | | **3** |
| **Gastrointestinal plastic?** | | Proventriculus | | | | | Y | Ventriculus | | | Y | **Bursa of Fabricius?** | | | | Y | |
| **Complete GIT?** | | Y | | **If no, details:** | | | | | | | | | | | | | |

**Summary of abnormal/significant findings if present:**

**Scavenging:** Not scavenged.

**External:** Thin. No obvious signs of trauma.

**Internal:** Water in lungs. Moderate amount of ingesta containing plastic in the proventriculus. A large number of plastics in the ventriculus with minimal other ingesta present.

**ST-Wol-5**

| **Collection Year** | | | 2021 | | | | | | **Bird ID** | | | | ST-Wol-5 | | | | |
| --- | --- | --- | --- | --- | --- | --- | --- | --- | --- | --- | --- | --- | --- | --- | --- | --- | --- |
| **Freshness** | | | FFF (Very fresh) | | | | | | **Completeness** | | | | CC | | | | |
| **Body Condition** | Intestinal fat (0-3) | | | | 3 | Subcutaneous fat (0-3) | | | | 3 | Breast muscle (0-3) | | | 3 | BCI (0-9) | | **9** |
| **Gastrointestinal plastic?** | | Proventriculus | | | | | N | Ventriculus | | | Y | **Bursa of Fabricius?** | | | | Y | |
| **Complete GIT?** | | Y | | **If no, details:** | | | | | | | | | | | | | |

**Summary of abnormal/significant findings if present:**

**Scavenging:** Not scavenged.

**External:** Very fat body condition. Bruised right tongue with an abrasion present. Bruising on head.

**Internal:** No obvious water in airways. No obvious trauma apart from the head. Bleeding cranial cavity. Proventriculus almost empty with no plastic. Ventriculus empty apart from three pieces of plastic and one rock.

**ST-Wol-6**

| **Collection Year** | | | 2021 | | | | | | **Bird ID** | | | | ST-Wol-6 | | | | |
| --- | --- | --- | --- | --- | --- | --- | --- | --- | --- | --- | --- | --- | --- | --- | --- | --- | --- |
| **Freshness** | | | FFF (Very fresh) | | | | | | **Completeness** | | | | CC | | | | |
| **Body Condition** | Intestinal fat (0-3) | | | | 1 | Subcutaneous fat (0-3) | | | | 1 | Breast muscle (0-3) | | | 1 | BCI (0-9) | | **3** |
| **Gastrointestinal plastic?** | | Proventriculus | | | | | Y | Ventriculus | | | Y | **Bursa of Fabricius?** | | | | Y | |
| **Complete GIT?** | | Y | | **If no, details:** | | | | | | | | | | | | | |

**Summary of abnormal/significant findings if present:**

**Scavenging:** Not scavenged.

**External:** Thin. No obvious trauma.

**Internal:** Water present in lungs. Liver small but otherwise normal in appearance. Little blood in the heart chambers. A very large amount of ingesta (oil and food) was present in the proventriculus with a single piece of plastic (medium size). Many pieces of plastic in the ventriculus, one rock, little other ingesta.

**ST-Wol-7**

| **Collection Year** | | | 2021 | | | | | | **Bird ID** | | | | ST-Wol-7 | | | | |
| --- | --- | --- | --- | --- | --- | --- | --- | --- | --- | --- | --- | --- | --- | --- | --- | --- | --- |
| **Freshness** | | | FFF (Very fresh) | | | | | | **Completeness** | | | | CC | | | | |
| **Body Condition** | Intestinal fat (0-3) | | | | 1 | Subcutaneous fat (0-3) | | | | 1 | Breast muscle (0-3) | | | 1 | BCI (0-9) | | **3** |
| **Gastrointestinal plastic?** | | Proventriculus | | | | | N | Ventriculus | | | Y | **Bursa of Fabricius?** | | | | Y | |
| **Complete GIT?** | | Y | | **If no, details:** | | | | | | | | | | | | | |

**Summary of abnormal/significant findings if present:**

**Scavenging:** Not scavenged.

**External:** Thin. No obvious trauma.

**Internal:** Water in lungs. Little blood in heart chambers. Proventriculus empty. Ventriculus empty apart from two pieces of plastic.

**ST-Wol-8**

| **Collection Year** | | | 2021 | | | | | | **Bird ID** | | | | ST-Wol-8 | | | | |
| --- | --- | --- | --- | --- | --- | --- | --- | --- | --- | --- | --- | --- | --- | --- | --- | --- | --- |
| **Freshness** | | | FFF (Very fresh) | | | | | | **Completeness** | | | | CC | | | | |
| **Body Condition** | Intestinal fat (0-3) | | | | 2 | Subcutaneous fat (0-3) | | | | 1 | Breast muscle (0-3) | | | 1 | BCI (0-9) | | **4** |
| **Gastrointestinal plastic?** | | Proventriculus | | | | | Y | Ventriculus | | | N | **Bursa of Fabricius?** | | | | Y | |
| **Complete GIT?** | | Y | | **If no, details:** | | | | | | | | | | | | | |

**Summary of abnormal/significant findings if present:**

**Scavenging:** Not scavenged.

**External:** Low-moderate body condition. No obvious trauma.

**Internal:** Water in lungs. Little blood in heart chambers. One reasonable sized piece of plastic in the proventriculus with moderate amounts of other ingesta. Ventriculus contained a small amount of ingesta with eight squid beaks (no plastic).

**ST-Wol-9**

| **Collection Year** | | | 2021 | | | | | | **Bird ID** | | | | ST-Wol-9 | | | | |
| --- | --- | --- | --- | --- | --- | --- | --- | --- | --- | --- | --- | --- | --- | --- | --- | --- | --- |
| **Freshness** | | | FFF (Very fresh) | | | | | | **Completeness** | | | | CC | | | | |
| **Body Condition** | Intestinal fat (0-3) | | | | 2 | Subcutaneous fat (0-3) | | | | 1 | Breast muscle (0-3) | | | 1 | BCI (0-9) | | **4** |
| **Gastrointestinal plastic?** | | Proventriculus | | | | | N | Ventriculus | | | Y | **Bursa of Fabricius?** | | | | Y | |
| **Complete GIT?** | | Y | | **If no, details:** | | | | | | | | | | | | | |

**Summary of abnormal/significant findings if present:**

**Scavenging:** Not scavenged.

**External:** Low-moderate body condition. Peck like marks on the head with bruising.

**Internal:** No obvious water in airways. Bleeding in cerebral cavity. Little blood in heart chambers. Proventriculus only had a small amount of ingesta and no plastic. Ventriculus had a large amount of ingesta and contains plastic.

**ST-Wol-10**

| **Collection Year** | | | 2021 | | | | | | **Bird ID** | | | | ST-Wol-10 | | | | |
| --- | --- | --- | --- | --- | --- | --- | --- | --- | --- | --- | --- | --- | --- | --- | --- | --- | --- |
| **Freshness** | | | FFF (Very fresh) | | | | | | **Completeness** | | | | CC | | | | |
| **Body Condition** | Intestinal fat (0-3) | | | | 3 | Subcutaneous fat (0-3) | | | | 3 | Breast muscle (0-3) | | | 1 | BCI (0-9) | | **7** |
| **Gastrointestinal plastic?** | | Proventriculus | | | | | N | Ventriculus | | | Y | **Bursa of Fabricius?** | | | | Y | |
| **Complete GIT?** | | Y | | **If no, details:** | | | | | | | | | | | | | |

**Summary of abnormal/significant findings if present:**

**Scavenging:** Not scavenged.

**External:** Good body condition. Peck marks and bruising on head. Was seen to have been attacked by gulls.

**Internal:** No obvious water in airways. Little blood in heart chambers. Although this bird had large fat stores, its pectoral muscle size was low (1/3). The proventriculus was distended with fluid that appeared to be mostly oil, no plastic. Low amounts of ingesta in the intestine. Little food, but a large number of plastic pieces were present in the ventriculus and suspected to be causing obstructive disease.

**ST-Wol-11**

| **Collection Year** | | | 2021 | | | | | | **Bird ID** | | | | ST-Wol-11 | | | | |
| --- | --- | --- | --- | --- | --- | --- | --- | --- | --- | --- | --- | --- | --- | --- | --- | --- | --- |
| **Freshness** | | | FFF (Very fresh) | | | | | | **Completeness** | | | | C | | | | |
| **Body Condition** | Intestinal fat (0-3) | | | | 3 | Subcutaneous fat (0-3) | | | | 3 | Breast muscle (0-3) | | | 2 | BCI (0-9) | | **8** |
| **Gastrointestinal plastic?** | | Proventriculus | | | | | Y | Ventriculus | | | N | **Bursa of Fabricius?** | | | | Y | |
| **Complete GIT?** | | Y | | **If no, details:** | | | | | | | | | | | | | |

**Summary of abnormal/significant findings if present:**

**Scavenging:** Some breast muscle removed with an opening into the coelomic cavity. Some damage to lungs, heart and air sacs in cranial cavity, minimal tissue removed. Uncertain if all damage done by gulls was all post-mortem, however since most of blood has remained in heart chambers and there is minimal blood in the coelomic cavity, it appears likely it had clotted there before damage was done to this area.

**External:** Fat body condition.

**Internal:** No obvious fluid in airways (but some damage to parts of the lungs and air sacs due to scavenging). Gastrointestinal tract intact. Moderate amount of ingesta in the proventriculus with a reasonable sized piece of plastic (broken piece of cylindrical user plastic). Ventriculus was empty. Reasonable amounts of ingesta in the intestines.

**ST-Wol-12**

| **Collection Year** | | | 2021 | | | | | | **Bird ID** | | | | ST-Wol-12 | | | | |
| --- | --- | --- | --- | --- | --- | --- | --- | --- | --- | --- | --- | --- | --- | --- | --- | --- | --- |
| **Freshness** | | | FFF (Very fresh) | | | | | | **Completeness** | | | | I | | | | |
| **Body Condition** | Intestinal fat (0-3) | | | | 3 | Subcutaneous fat (0-3) | | | | 3 | Breast muscle (0-3) | | | - | BCI (0-9) | | **-** |
| **Gastrointestinal plastic?** | | Proventriculus | | | | | N | Ventriculus | | | Y | **Bursa of Fabricius?** | | | | Y | |
| **Complete GIT?** | | Y | | **If no, details:** | | | | | | | | | | | | | |

**Summary of abnormal/significant findings if present:**

**Scavenging:** Most of breast muscle removed (cannot breast muscle score). Some of the subcutaneous fat removed in the upper chest area. Hole into the upper coelomic cavity. Uncertain if all damage was postmortem.

**External:** Well developed, large, fat bird.

**Internal:** Internal organs intact. No obvious water in airways. Right kidney slightly smaller than left and a lighter tan in colour. Proventriculus empty. Ventriculus contains moderate ingesta with plastic, rocks and large numbers of squid beaks. Reasonable amount of ingesta in the intestines.

**ST-Wol-13**

| **Collection Year** | | | 2021 | | | | | | **Bird ID** | | | | ST-Wol-13 | | | | |
| --- | --- | --- | --- | --- | --- | --- | --- | --- | --- | --- | --- | --- | --- | --- | --- | --- | --- |
| **Freshness** | | | FFF (Very fresh) | | | | | | **Completeness** | | | | I | | | | |
| **Body Condition** | Intestinal fat (0-3) | | | | 3 | Subcutaneous fat (0-3) | | | | 2 | Breast muscle (0-3) | | | 2 | BCI (0-9) | | **7** |
| **Gastrointestinal plastic?** | | Proventriculus | | | | | - | Ventriculus | | | - | **Bursa of Fabricius?** | | | | Y | |
| **Complete GIT?** | | N | | **If no, details:** Mid oesophagus to ventriculus missing. | | | | | | | | | | | | | |

**Summary of abnormal/significant findings if present:**

**Scavenging:** Removed a significant amount of subcutaneous fat (especially over right breast), some of the pectoral muscle (mostly on the right side), and a hole was present into the coelomic cavity. Right lung and the gastrointestinal tract from mid oesophagus to the ventriculus was removed. Left lung is damaged. Uncertain if all damage was postmortem.

**External:** Good body condition.

**Internal:** Degree of damage to airways makes it difficult to assess aspiration of water. Liver is on the smaller side but otherwise normal in appearance. Little blood in the heart chambers. No plastic analysis due to damage to gut.

**ST-Wol-14**

| **Collection Year** | | | 2021 | | | | | | **Bird ID** | | | | ST-Wol-14 | | | | |
| --- | --- | --- | --- | --- | --- | --- | --- | --- | --- | --- | --- | --- | --- | --- | --- | --- | --- |
| **Freshness** | | | FFF (Very fresh) | | | | | | **Completeness** | | | | CC | | | | |
| **Body Condition** | Intestinal fat (0-3) | | | | 0 | Subcutaneous fat (0-3) | | | | 0 | Breast muscle (0-3) | | | 1 | BCI (0-9) | | **1** |
| **Gastrointestinal plastic?** | | Proventriculus | | | | | N | Ventriculus | | | N | **Bursa of Fabricius?** | | | | Y | |
| **Complete GIT?** | | Y | | **If no, details:** | | | | | | | | | | | | | |

**Summary of abnormal/significant findings if present:**

**Scavenging:** Not scavenged.

**External:** Emaciated. No obvious signs of trauma.

**Internal:** Water in lungs and air sacs. Liver a bit larger than usual but otherwise normal in appearance. Kidneys are large and a darker red hue than typical. Proventriculus almost empty with no plastic. Ventriculus contained a moderate amount of ingesta, with large numbers of squid beaks and small rocks. No plastic. Low amounts of ingesta in the intestines.

**ST-Wol-15**

| **Collection Year** | | | 2021 | | | | | | **Bird ID** | | | | ST-Wol-15 | | | | |
| --- | --- | --- | --- | --- | --- | --- | --- | --- | --- | --- | --- | --- | --- | --- | --- | --- | --- |
| **Freshness** | | | FFF (Very fresh) | | | | | | **Completeness** | | | | CC | | | | |
| **Body Condition** | Intestinal fat (0-3) | | | | 3 | Subcutaneous fat (0-3) | | | | 3 | Breast muscle (0-3) | | | 2 | BCI (0-9) | | **8** |
| **Gastrointestinal plastic?** | | Proventriculus | | | | | N | Ventriculus | | | Y | **Bursa of Fabricius?** | | | | Y | |
| **Complete GIT?** | | Y | | **If no, details:** | | | | | | | | | | | | | |

**Summary of abnormal/significant findings if present:**

**Scavenging:** Not scavenged.

**External:** Fat body condition. No obvious trauma.

**Internal:** Water in air sacs. Heart chambers distended with blood. Liver larger than typical but otherwise normal in appearance. Proventriculus empty. Ventriculus contained one plastic pellet, large numbers or rocks, and little other ingesta. Reasonable amount of ingesta in intestine.

**ST-Wol-16**

| **Collection Year** | | | 2021 | | | | | | **Bird ID** | | | | ST-Wol-16 | | | | |
| --- | --- | --- | --- | --- | --- | --- | --- | --- | --- | --- | --- | --- | --- | --- | --- | --- | --- |
| **Freshness** | | | FFF (Very fresh) | | | | | | **Completeness** | | | | CC | | | | |
| **Body Condition** | Intestinal fat (0-3) | | | | 3 | Subcutaneous fat (0-3) | | | | 2 | Breast muscle (0-3) | | | 2 | BCI (0-9) | | **7** |
| **Gastrointestinal plastic?** | | Proventriculus | | | | | Y | Ventriculus | | | Y | **Bursa of Fabricius?** | | | | Y | |
| **Complete GIT?** | | Y | | **If no, details:** | | | | | | | | | | | | | |

**Summary of abnormal/significant findings if present:**

**Scavenging:** Not scavenged.

**External:** Good body condition. Trauma to head with peck marks and bruising.

**Internal:** Skull fracture (frontal bone) with haemorrhage. No obvious water in airways. Moderate amounts of ingesta in the proventriculus with one piece of plastic. Moderate amounts of ingesta in the ventriculus with plastic. Moderate ingesta in intestines.

**ST-Wol-17**

| **Collection Year** | | | 2021 | | | | | | **Bird ID** | | | | ST-Wol-17 | | | | |
| --- | --- | --- | --- | --- | --- | --- | --- | --- | --- | --- | --- | --- | --- | --- | --- | --- | --- |
| **Freshness** | | | FFF (Very fresh) | | | | | | **Completeness** | | | | CC | | | | |
| **Body Condition** | Intestinal fat (0-3) | | | | 3 | Subcutaneous fat (0-3) | | | | 3 | Breast muscle (0-3) | | | 2 | BCI (0-9) | | **8** |
| **Gastrointestinal plastic?** | | Proventriculus | | | | | N | Ventriculus | | | N | **Bursa of Fabricius?** | | | | Y | |
| **Complete GIT?** | | Y | | **If no, details:** | | | | | | | | | | | | | |

**Summary of abnormal/significant findings if present:**

**Scavenging:** Not scavenged.

**External:** Fat body condition. No obvious trauma.

**Internal:** No obvious water in airways. Small black recessed spot (~1mm diameter) on the right liver lobe. Otherwise liver appeared normal. One piece of pale-yellow glass ~5mm diameter in ventriculus, otherwise no anthropogenic debris in the gastrointestinal tract. Moderate-high amount of ingesta in the proventriculus with lots of sand and moderate numbers of squid beaks. Ventriculus empty apart from the glass fragment. No visible ulceration of the ventriculus or proventriculus.

**ST-Wol-18**

| **Collection Year** | | | 2021 | | | | | | **Bird ID** | | | | ST-Wol-18 | | | | |
| --- | --- | --- | --- | --- | --- | --- | --- | --- | --- | --- | --- | --- | --- | --- | --- | --- | --- |
| **Freshness** | | | FFF (Very fresh) | | | | | | **Completeness** | | | | I | | | | |
| **Body Condition** | Intestinal fat (0-3) | | | | 3 | Subcutaneous fat (0-3) | | | | 2 | Breast muscle (0-3) | | | 3 | BCI (0-9) | | **8** |
| **Gastrointestinal plastic?** | | Proventriculus | | | | | N | Ventriculus | | | Y | **Bursa of Fabricius?** | | | | Y | |
| **Complete GIT?** | | Y | | **If no, details:** | | | | | | | | | | | | | |

**Summary of abnormal/significant findings if present:**

**Scavenging:** Significant removal of soft tissues from the head, neck and thoracic inlet area. Hole open into the coelomic cavity in this area. Some rostral pectoral muscle removed. Damage to lungs. Found at the water’s edge on the beach.

**External:** Fat body condition.

**Internal:** Water and sand contamination via the hole in the coelomic cavity from waves on the shore. Damaged lungs, unable to assess for aspiration of water. Liver and kidneys look haemorrhagic. Suspect at least some trauma occurred before death. Proventriculus contained one piece of cuttlefish and almost no other ingesta. Ventriculus contained moderate amounts of ingesta with one plastic pellet, two cuttlefish pieces and a rock.

**ST-Wol-19**

| **Collection Year** | | | 2021 | | | | | | **Bird ID** | | | | ST-Wol-19 | | | | |
| --- | --- | --- | --- | --- | --- | --- | --- | --- | --- | --- | --- | --- | --- | --- | --- | --- | --- |
| **Freshness** | | | FFF (Very fresh) | | | | | | **Completeness** | | | | C | | | | |
| **Body Condition** | Intestinal fat (0-3) | | | | 3 | Subcutaneous fat (0-3) | | | | 3 | Breast muscle (0-3) | | | 2 | BCI (0-9) | | **8** |
| **Gastrointestinal plastic?** | | Proventriculus | | | | | N | Ventriculus | | | Y | **Bursa of Fabricius?** | | | | Y | |
| **Complete GIT?** | | Y | | **If no, details:** | | | | | | | | | | | | | |

**Summary of abnormal/significant findings if present:**

**Scavenging:** Most of left pectoral muscle removed. Internal organs intact.

**External:** Fat body condition. Some bruising/bleeding around the chest area, especially on the left.

**Internal:** No obvious water in airways. Moderate-high amount of ingesta in the proventriculus without plastic. Ventriculus had a small amount of ingesta containing plastic.

**ST-Wol-20**

| **Collection Year** | | | 2021 | | | | | | **Bird ID** | | | | ST-Wol-20 | | | | |
| --- | --- | --- | --- | --- | --- | --- | --- | --- | --- | --- | --- | --- | --- | --- | --- | --- | --- |
| **Freshness** | | | FFF (Very fresh) | | | | | | **Completeness** | | | | CC | | | | |
| **Body Condition** | Intestinal fat (0-3) | | | | 0 | Subcutaneous fat (0-3) | | | | 0 | Breast muscle (0-3) | | | 0 | BCI (0-9) | | **0** |
| **Gastrointestinal plastic?** | | Proventriculus | | | | | N | Ventriculus | | | Y | **Bursa of Fabricius?** | | | | Y | |
| **Complete GIT?** | | Y | | **If no, details:** | | | | | | | | | | | | | |

**Summary of abnormal/significant findings if present:**

**Scavenging:** Not scavenged.

**External:** Emaciated. Small bird.

**Internal:** Water present in air sacs. Gall bladder was very large and full of bile. Liver small but otherwise normal in appearance. Proventriculus empty. Small amount of ingesta containing plastic in the ventriculus. Only a small amount of ingesta in the intestines.

**ST-Wol-21**

| **Collection Year** | | | 2021 | | | | | | **Bird ID** | | | | ST-Wol-21 | | | | |
| --- | --- | --- | --- | --- | --- | --- | --- | --- | --- | --- | --- | --- | --- | --- | --- | --- | --- |
| **Freshness** | | | FFF (Very fresh) | | | | | | **Completeness** | | | | C | | | | |
| **Body Condition** | Intestinal fat (0-3) | | | | 0 | Subcutaneous fat (0-3) | | | | 0 | Breast muscle (0-3) | | | 0 | BCI (0-9) | | **0** |
| **Gastrointestinal plastic?** | | Proventriculus | | | | | N | Ventriculus | | | Y | **Bursa of Fabricius?** | | | | Y | |
| **Complete GIT?** | | Y | | **If no, details:** | | | | | | | | | | | | | |

**Summary of abnormal/significant findings if present:**

**Scavenging:** Minor scavenging skin/muscle around head.

**External:** Emaciated. Small bird. Immature appearing flight feathers all retained feather sheath covering some of the barbs. Bruising and trauma to soft tissues around head.

**Internal:** Fractured frontal bone and bleeding into cranial cavity. Small amount of ingesta in proventriculus, with a large piece of semi-digested kelp, no plastic. Ventriculus contained a large amount of material (~2mL) and felt solid/impacted: large piece of twisted, semi-digested kelp tangled with four pieces of plastic of up to 8.7mm in the largest dimension likely causing obstructive disease. Small amount of ingesta in the intestines only.

**ST-Wol-22**

| **Collection Year** | | | 2021 | | | | | | **Bird ID** | | | | ST-Wol-22 | | | | |
| --- | --- | --- | --- | --- | --- | --- | --- | --- | --- | --- | --- | --- | --- | --- | --- | --- | --- |
| **Freshness** | | | FFF (Very fresh) | | | | | | **Completeness** | | | | C | | | | |
| **Body Condition** | Intestinal fat (0-3) | | | | 2 | Subcutaneous fat (0-3) | | | | 2 | Breast muscle (0-3) | | | 3 | BCI (0-9) | | **7** |
| **Gastrointestinal plastic?** | | Proventriculus | | | | | N | Ventriculus | | | Y | **Bursa of Fabricius?** | | | | Y | |
| **Complete GIT?** | | Y | | **If no, details:** | | | | | | | | | | | | | |

**Summary of abnormal/significant findings if present:**

**Scavenging:** Some subcutaneous fat and pectoral muscle removed from the left side of the chest.

**External:** Good body condition.

**Internal:** Some evidence of bleeding from pectoral muscles. No obvious water in airways. Proventriculus was empty. Ventriculus contained plastic, many squid beaks and a small amount of other liquid ingesta. Small amount of ingesta in intestines.

**ST-Wol-23**

| **Collection Year** | | | 2021 | | | | | | **Bird ID** | | | | ST-Wol-23 | | | | |
| --- | --- | --- | --- | --- | --- | --- | --- | --- | --- | --- | --- | --- | --- | --- | --- | --- | --- |
| **Freshness** | | | FFF (Very fresh) | | | | | | **Completeness** | | | | C | | | | |
| **Body Condition** | Intestinal fat (0-3) | | | | 2 | Subcutaneous fat (0-3) | | | | 1 | Breast muscle (0-3) | | | 2 | BCI (0-9) | | **5** |
| **Gastrointestinal plastic?** | | Proventriculus | | | | | Y | Ventriculus | | | Y | **Bursa of Fabricius?** | | | | Y | |
| **Complete GIT?** | | Y | | **If no, details:** | | | | | | | | | | | | | |

**Summary of abnormal/significant findings if present:**

**Scavenging:** Mild removal of soft tissues around the thoracic inlet area (muscle and fat.)

**External:** Moderate body condition. Peck like marks and bruising on head.

**Internal:** No obvious water in airways. Fractured frontal bone with bleeding into cranial cavity. Proventriculus had a small amount of ingesta with multiple pieces of plastic. Ventriculus contained a small amount of ingesta with multiple pieces of plastic and a few squid beaks. Small amount of ingesta in intestines.

**ST-Wol-24**

| **Collection Year** | | | 2021 | | | | | | **Bird ID** | | | | ST-Wol-24 | | | | |
| --- | --- | --- | --- | --- | --- | --- | --- | --- | --- | --- | --- | --- | --- | --- | --- | --- | --- |
| **Freshness** | | | FFF (Very fresh) | | | | | | **Completeness** | | | | CC | | | | |
| **Body Condition** | Intestinal fat (0-3) | | | | 1 | Subcutaneous fat (0-3) | | | | 0 | Breast muscle (0-3) | | | 0 | BCI (0-9) | | **1** |
| **Gastrointestinal plastic?** | | Proventriculus | | | | | N | Ventriculus | | | Y | **Bursa of Fabricius?** | | | | Y | |
| **Complete GIT?** | | Y | | **If no, details:** | | | | | | | | | | | | | |

**Summary of abnormal/significant findings if present:**

**Scavenging:** Not scavenged.

**External:** Emaciated. No obvious external trauma noted.

**Internal:** No obvious water in airways. Gall bladder very large and full of bile. Small amount of ingesta in the proventriculus without plastic. Ventriculus contained a small amount of ingesta with plastic. Small amount of ingesta in the intestines.

**ST-Wol-25**

| **Collection Year** | | | 2021 | | | | | | **Bird ID** | | | | ST-Wol-25 | | | | |
| --- | --- | --- | --- | --- | --- | --- | --- | --- | --- | --- | --- | --- | --- | --- | --- | --- | --- |
| **Freshness** | | | FFF (Very fresh) | | | | | | **Completeness** | | | | C | | | | |
| **Body Condition** | Intestinal fat (0-3) | | | | 2 | Subcutaneous fat (0-3) | | | | 1 | Breast muscle (0-3) | | | 1 | BCI (0-9) | | **4** |
| **Gastrointestinal plastic?** | | Proventriculus | | | | | N | Ventriculus | | | Y | **Bursa of Fabricius?** | | | | Y | |
| **Complete GIT?** | | Y | | **If no, details:** | | | | | | | | | | | | | |

**Summary of abnormal/significant findings if present:**

**Scavenging:** Minor scavenging of fat and pectoral muscle on the right chest area.

**External:** Low-moderate body condition. No obvious bleeding around scavenged area.

**Internal:** Some water noted in air sacs. Proventriculus had moderate ingesta and no plastic. Ventriculus contained a large amount of ingesta with large numbers of plastic pieces. Moderate ingesta intestines.

**ST-Wol-26**

| **Collection Year** | | | 2021 | | | | | | **Bird ID** | | | | ST-Wol-26 | | | | |
| --- | --- | --- | --- | --- | --- | --- | --- | --- | --- | --- | --- | --- | --- | --- | --- | --- | --- |
| **Freshness** | | | FFF (Very fresh) | | | | | | **Completeness** | | | | II | | | | |
| **Body Condition** | Intestinal fat (0-3) | | | | - | Subcutaneous fat (0-3) | | | | - | Breast muscle (0-3) | | | - | BCI (0-9) | | **-** |
| **Gastrointestinal plastic?** | | Proventriculus | | | | | - | Ventriculus | | | - | **Bursa of Fabricius?** | | | | Y | |
| **Complete GIT?** | | N | | **If no, details:** Mid to caudal oesophagus, proventriculus, ventriculus, and most of intestines removed. | | | | | | | | | | | | | |

**Summary of abnormal/significant findings if present:**

**Scavenging:** Major scavenging. Not enough remaining tissues to perform accurate body condition scores. Most of the gastrointestinal tract removed so no plastic analysis performed. Liver, heart and lungs missing.

**External:** Severe scavenging damage.

**Internal:** Some abdominal fat present in the caudal coelomic cavity. Kidneys appeared normal. Severe scavenging.

**ST-Wol-27**

| **Collection Year** | | | 2021 | | | | | | **Bird ID** | | | | ST-Wol-27 | | | | |
| --- | --- | --- | --- | --- | --- | --- | --- | --- | --- | --- | --- | --- | --- | --- | --- | --- | --- |
| **Freshness** | | | FFF (Very fresh) | | | | | | **Completeness** | | | | C | | | | |
| **Body Condition** | Intestinal fat (0-3) | | | | 2 | Subcutaneous fat (0-3) | | | | 2 | Breast muscle (0-3) | | | 2 | BCI (0-9) | | **6** |
| **Gastrointestinal plastic?** | | Proventriculus | | | | | Y | Ventriculus | | | Y | **Bursa of Fabricius?** | | | | Y | |
| **Complete GIT?** | | Y | | **If no, details:** | | | | | | | | | | | | | |

**Summary of abnormal/significant findings if present:**

**Scavenging:** Badly scavenged around the neck and thoracic inlet area with removal of fat and muscle from this area. Hole into the body cavity in the thoracic inlet area. Bird present on the edge of the waterline on the beach. Lung present but damaged.

**External:** High-moderate body condition. Quite a large, well developed bird.

**Internal:** Likely water contamination after death via the hole in the thoracic inlet area as there was free water with some sand in the coelomic cavity making assessment of possible drowning difficult. Lungs damaged via scavenging activity. Liver a lighter tan-brown colour, rounded and friable. (Suspected fatty liver.) Proventriculus only contained a small amount of ingesta and one piece of plastic. Ventriculus contained moderate amounts of ingesta with multiple pieces of plastic.

**ST-Wol-28**

| **Collection Year** | | | 2021 | | | | | | **Bird ID** | | | | ST-Wol-28 | | | | |
| --- | --- | --- | --- | --- | --- | --- | --- | --- | --- | --- | --- | --- | --- | --- | --- | --- | --- |
| **Freshness** | | | FFF (Very fresh) | | | | | | **Completeness** | | | | I | | | | |
| **Body Condition** | Intestinal fat (0-3) | | | | 2 | Subcutaneous fat (0-3) | | | | 1 | Breast muscle (0-3) | | | 2 | BCI (0-9) | | **5** |
| **Gastrointestinal plastic?** | | Proventriculus | | | | | N | Ventriculus | | | Y | **Bursa of Fabricius?** | | | | Y | |
| **Complete GIT?** | | Y | | **If no, details:** | | | | | | | | | | | | | |

**Summary of abnormal/significant findings if present:**

**Scavenging:** Scavenged head with brain damaged.

**External:** Moderate body condition. Some bleeding in cranial cavity/head area.

**Internal:** No obvious water in airways. Liver on the smaller side but otherwise normal in appearance. Little blood in the heart chambers. Proventriculus empty. Ventriculus had a small amount of ingesta with plastic. Small amount of ingesta in intestines.

**ST-Wol-29**

| **Collection Year** | | | 2021 | | | | | | **Bird ID** | | | | ST-Wol-29 | | | | |
| --- | --- | --- | --- | --- | --- | --- | --- | --- | --- | --- | --- | --- | --- | --- | --- | --- | --- |
| **Freshness** | | | FFF (Very fresh) | | | | | | **Completeness** | | | | I | | | | |
| **Body Condition** | Intestinal fat (0-3) | | | | 3 | Subcutaneous fat (0-3) | | | | 3 | Breast muscle (0-3) | | | 2 | BCI (0-9) | | **8** |
| **Gastrointestinal plastic?** | | Proventriculus | | | | | Y | Ventriculus | | | Y | **Bursa of Fabricius?** | | | | Y | |
| **Complete GIT?** | | Y | | **If no, details:** | | | | | | | | | | | | | |

**Summary of abnormal/significant findings if present:**

**Scavenging:** Scavenged head with damage to brain. About half of the right pectoral muscle removed.

**External:** Fat body condition. Feathers relatively long and mature. Bruising around head.

**Internal:** No obvious water in airways. Bleeding in cranial cavity with brain damaged by scavenging. Frontal and parietal bone fractures. Liver normal in shape and colour but friable. Proventriculus contained plastic with only a small amount of other food ingesta. Ventriculus contained a large number of plastic pieces and moderate ingesta. Reasonable amounts of ingesta in the intestines.

**ST-Wol-30**

| **Collection Year** | | | 2021 | | | | | | **Bird ID** | | | | ST-Wol-30 | | | | |
| --- | --- | --- | --- | --- | --- | --- | --- | --- | --- | --- | --- | --- | --- | --- | --- | --- | --- |
| **Freshness** | | | FFF (Very fresh) | | | | | | **Completeness** | | | | I | | | | |
| **Body Condition** | Intestinal fat (0-3) | | | | 3 | Subcutaneous fat (0-3) | | | | 3 | Breast muscle (0-3) | | | 3 | BCI (0-9) | | **9** |
| **Gastrointestinal plastic?** | | Proventriculus | | | | | N | Ventriculus | | | N | **Bursa of Fabricius?** | | | | Y | |
| **Complete GIT?** | | Y | | **If no, details:** | | | | | | | | | | | | | |

**Summary of abnormal/significant findings if present:**

**Scavenging:** Removal of much of the subcutaneous fat and pectoral muscle on the right chest with hole into the coelomic cavity. Some muscle removed from the upper left chest area. Most of right lung removed.

**External:** Very fat body condition. Large bird.

**Internal:** Kidneys and liver appear large but otherwise normal in shape and colour. Watery blood in the coelomic cavity. Appears to have had bleeding prior to death, however uncertain if water was present in the airways and released into coelomic cavity on damage to air sacs and right lung, or if water entered after death via the hole in the body wall and due to location on water’s edge. Small amount of ingesta without plastic in the proventriculus, ventriculus and intestines. Ventriculus also contained one squid beak.

**ST-Wol-31**

| **Collection Year** | | | 2021 | | | | | | **Bird ID** | | | | ST-Wol-31 | | | | |
| --- | --- | --- | --- | --- | --- | --- | --- | --- | --- | --- | --- | --- | --- | --- | --- | --- | --- |
| **Freshness** | | | FFF (Very fresh) | | | | | | **Completeness** | | | | CC | | | | |
| **Body Condition** | Intestinal fat (0-3) | | | | 0 | Subcutaneous fat (0-3) | | | | 0 | Breast muscle (0-3) | | | 0 | BCI (0-9) | | **0** |
| **Gastrointestinal plastic?** | | Proventriculus | | | | | Y | Ventriculus | | | Y | **Bursa of Fabricius?** | | | | Y | |
| **Complete GIT?** | | Y | | **If no, details:** | | | | | | | | | | | | | |

**Summary of abnormal/significant findings if present:**

**Scavenging:** Not scavenged.

**External:** Emaciated. No sign of external trauma. Small bird with more immature flight feathers than average.

**Internal:** Water observed in air sacs. Small liver, otherwise normal in colour and shape. Small-moderate amount of ingesta in proventriculus with plastic. Ventriculus had many pieces of plastic with only a small amount of other ingesta. Only a small amount of ingesta in the intestines.

**ST-Wol-32**

| **Collection Year** | | | 2021 | | | | | | **Bird ID** | | | | ST-Wol-32 | | | | |
| --- | --- | --- | --- | --- | --- | --- | --- | --- | --- | --- | --- | --- | --- | --- | --- | --- | --- |
| **Freshness** | | | FFF (Very fresh) | | | | | | **Completeness** | | | | CC | | | | |
| **Body Condition** | Intestinal fat (0-3) | | | | 3 | Subcutaneous fat (0-3) | | | | 2 | Breast muscle (0-3) | | | 3 | BCI (0-9) | | **8** |
| **Gastrointestinal plastic?** | | Proventriculus | | | | | N | Ventriculus | | | Y | **Bursa of Fabricius?** | | | | Y | |
| **Complete GIT?** | | Y | | **If no, details:** | | | | | | | | | | | | | |

**Summary of abnormal/significant findings if present:**

**Scavenging:** Not scavenged.

**External:** Fat body condition. Very large, well grown bird. Feathers relatively long and mature. No sign of external trauma.

**Internal:** Water present in air sacs. Proventriculus almost empty with minimal ingesta and no plastic. Ventriculus contained moderate amounts of ingesta and many pieces of plastic. Reasonable amount of ingesta in the intestines.

**ST-Wol-33**

| **Collection Year** | | | 2021 | | | | | | **Bird ID** | | | | ST-Wol-33 | | | | |
| --- | --- | --- | --- | --- | --- | --- | --- | --- | --- | --- | --- | --- | --- | --- | --- | --- | --- |
| **Freshness** | | | FFF (Very fresh) | | | | | | **Completeness** | | | | I | | | | |
| **Body Condition** | Intestinal fat (0-3) | | | | 3 | Subcutaneous fat (0-3) | | | | 2 | Breast muscle (0-3) | | | 1 | BCI (0-9) | | **6** |
| **Gastrointestinal plastic?** | | Proventriculus | | | | | Y | Ventriculus | | | Y | **Bursa of Fabricius?** | | | | Y | |
| **Complete GIT?** | | Y | | **If no, details:** | | | | | | | | | | | | | |

**Summary of abnormal/significant findings if present:**

**Scavenging:** A large amount of soft tissues (fat and muscle) removed from the neck, thoracic inlet (hole present into the coelomic cavity) and right chest. The pectoral muscle and subcutaneous fat has only been removed from the very cranial part of the left chest which is otherwise intact. Lungs were removed.

**External:** Externally appeared a bit underweight, however this is likely due to the below average mass of the pectoral muscles present (1/3). The fat stores were both in the higher range of the body condition scores. (Subcutaneous fat: 2/3, intestinal fat 3/3.) Medium sized bird.

**Internal:** Unable to assess for aspiration of water due to removal of both lungs during scavenging. Proventriculus and ventriculus contained plastic and only a small amount of other ingesta. Intestines contained a lot of gas and little ingesta.

**ST-Wol-34**

| **Collection Year** | | | 2021 | | | | | | **Bird ID** | | | | ST-Wol-34 | | | | |
| --- | --- | --- | --- | --- | --- | --- | --- | --- | --- | --- | --- | --- | --- | --- | --- | --- | --- |
| **Freshness** | | | FFF (Very fresh) | | | | | | **Completeness** | | | | I | | | | |
| **Body Condition** | Intestinal fat (0-3) | | | | 3 | Subcutaneous fat (0-3) | | | | 2 | Breast muscle (0-3) | | | 3 | BCI (0-9) | | **8** |
| **Gastrointestinal plastic?** | | Proventriculus | | | | | N | Ventriculus | | | - | **Bursa of Fabricius?** | | | | Y | |
| **Complete GIT?** | | N | | **If no, details:** Missing ventriculus and proximal intestines. | | | | | | | | | | | | | |

**Summary of abnormal/significant findings if present:**

**Scavenging:** Most of the muscle and subcutaneous fat removed on the right chest with a hole into the coelomic cavity in this area. Ventriculus, proximal part of the small intestines, right lung and the liver were removed.

**External:** Good body condition. Smaller than average bird.

**Internal:** Likely postmortem water and sand contamination of the coelomic cavity due to location the shore’s edge and hole into the body cavity, along with missing lung and damage to air sacs made assessment of whether water was aspirated difficult to determine. Little blood in heart chambers. Proventriculus did not contain plastic and had a small amount of other material that consisted mostly of feather fragments. Ventriculus/proximal intestines unable to be assessed for plastic due to scavenging.

**ST-Wol-35**

| **Collection Year** | | | 2021 | | | | | | **Bird ID** | | | | ST-Wol-35 | | | | |
| --- | --- | --- | --- | --- | --- | --- | --- | --- | --- | --- | --- | --- | --- | --- | --- | --- | --- |
| **Freshness** | | | FFF (Very fresh) | | | | | | **Completeness** | | | | C | | | | |
| **Body Condition** | Intestinal fat (0-3) | | | | 3 | Subcutaneous fat (0-3) | | | | 3 | Breast muscle (0-3) | | | 3 | BCI (0-9) | | **9** |
| **Gastrointestinal plastic?** | | Proventriculus | | | | | N | Ventriculus | | | Y | **Bursa of Fabricius?** | | | | Y | |
| **Complete GIT?** | | Y | | **If no, details:** | | | | | | | | | | | | | |

**Summary of abnormal/significant findings if present:**

**Scavenging:** Most of muscle around the thoracic inlet area had been scavenged with a hole into the coelomic cavity. Internal organs intact.

**External:** Fat body condition. Large, bird with more mature looking flight feathers. Uncertain if the damage done by the gulls was only postmortem or if some premortem damage had occurred.

**Internal:** No obvious water in airways. Little blood in heart chambers. Proventriculus contains mod-large amount of green ingesta without plastic with three squid beaks and cuttlefish fragments. One squid beak was large (22 mm in length.) Ventriculus contained a large amount of green ingesta with a few pieces of plastic. Reasonable amounts of ingesta in the intestines.

**ST-Wol-36**

| **Collection Year** | | | 2021 | | | | | | **Bird ID** | | | | ST-Wol-36 | | | | |
| --- | --- | --- | --- | --- | --- | --- | --- | --- | --- | --- | --- | --- | --- | --- | --- | --- | --- |
| **Freshness** | | | FFF (Very fresh) | | | | | | **Completeness** | | | | CC | | | | |
| **Body Condition** | Intestinal fat (0-3) | | | | 0 | Subcutaneous fat (0-3) | | | | 0 | Breast muscle (0-3) | | | 1 | BCI (0-9) | | **1** |
| **Gastrointestinal plastic?** | | Proventriculus | | | | | N | Ventriculus | | | Y | **Bursa of Fabricius?** | | | | Y | |
| **Complete GIT?** | | Y | | **If no, details:** | | | | | | | | | | | | | |

**Summary of abnormal/significant findings if present:**

**Scavenging:** Not scavenged.

**External:** Emaciated. Small bird. Head trauma from uncertain cause- bruising, small amount of broken skin and missing feathers.

**Internal:** Water in lungs and air sacs. Liver on the large side but otherwise normal in appearance. Proventriculus contained a small amount of green ingesta with pieces of twigs and feather fragments (no plastic.) Ventriculus contained a moderate amount of green ingesta with many pieces of plastic. Some ingesta in intestines.

**ST-Wol-37**

| **Collection Year** | | | 2021 | | | | | | **Bird ID** | | | | ST-Wol-37 | | | | |
| --- | --- | --- | --- | --- | --- | --- | --- | --- | --- | --- | --- | --- | --- | --- | --- | --- | --- |
| **Freshness** | | | FFF (Very fresh) | | | | | | **Completeness** | | | | I | | | | |
| **Body Condition** | Intestinal fat (0-3) | | | | 3 | Subcutaneous fat (0-3) | | | | 3 | Breast muscle (0-3) | | | 3 | BCI (0-9) | | **9** |
| **Gastrointestinal plastic?** | | Proventriculus | | | | | N | Ventriculus | | | N | **Bursa of Fabricius?** | | | | Y | |
| **Complete GIT?** | | Y | | **If no, details:** | | | | | | | | | | | | | |

**Summary of abnormal/significant findings if present:**

**Scavenging:** Most of muscle and subcutaneous fat removed from the right breast area with a hole into the body cavity. Lungs damaged and right lung removed.

**External:** Fat body condition. Reasonably large bird with relatively mature flight feathers.

**Internal:** Unable to assess for aspiration of water due to damage to lungs and air sacs. Little blood in heart chambers. Small amount of green ingesta in the proventriculus with many cuttlefish fragments, no plastic. Ventriculus was almost empty with a very small amount of green ingesta. Not a lot of ingesta in the intestines.

**ST-Wol-38**

| **Collection Year** | | | 2021 | | | | | | **Bird ID** | | | | ST-Wol-38 | | | | |
| --- | --- | --- | --- | --- | --- | --- | --- | --- | --- | --- | --- | --- | --- | --- | --- | --- | --- |
| **Freshness** | | | FFF (Very fresh) | | | | | | **Completeness** | | | |  | | | | |
| **Body Condition** | Intestinal fat (0-3) | | | | 3 | Subcutaneous fat (0-3) | | | | 3 | Breast muscle (0-3) | | | 2 | BCI (0-9) | | **8** |
| **Gastrointestinal plastic?** | | Proventriculus | | | | | N | Ventriculus | | | Y | **Bursa of Fabricius?** | | | | Y | |
| **Complete GIT?** | | Y | | **If no, details:** | | | | | | | | | | | | | |

**Summary of abnormal/significant findings if present:**

**Scavenging:** Removal of muscle and subcutaneous fat from the neck, thoracic inlet area and top (cranial) part of the pectoral muscles. Hole into body cavity present.

**External:** Fat body condition. Some evidence of bleeding in neck area.

**Internal:** No obvious water in airways. Liver was larger than average and a lighter tan-yellow hue. (Suspected fatty liver.) Little blood in heart chambers. Proventriculus contained no plastic and was almost empty, containing mostly feather fragments. Ventriculus contained moderate amounts of green ingesta with plastic, cuttlefish fragments, multiple small squid beaks and three small rocks. Some ingesta in intestines.

**ST-Wol-39**

| **Collection Year** | | | 2021 | | | | | | **Bird ID** | | | | ST-Wol-39 | | | | |
| --- | --- | --- | --- | --- | --- | --- | --- | --- | --- | --- | --- | --- | --- | --- | --- | --- | --- |
| **Freshness** | | | FFF (Very fresh) | | | | | | **Completeness** | | | | C | | | | |
| **Body Condition** | Intestinal fat (0-3) | | | | 2 | Subcutaneous fat (0-3) | | | | 2 | Breast muscle (0-3) | | | 2 | BCI (0-9) | | **6** |
| **Gastrointestinal plastic?** | | Proventriculus | | | | | N | Ventriculus | | | Y | **Bursa of Fabricius?** | | | | Y | |
| **Complete GIT?** | | Y | | **If no, details:** | | | | | | | | | | | | | |

**Summary of abnormal/significant findings if present:**

**Scavenging:** Some (mild scavenging) subcutaneous fat and muscle removed from the left chest and thoracic inlet area.

**External:** High moderate body condition. Medium sized bird. Uncertain if any gull damage was done premortem or scavenging only.

**Internal:** No water in airways. Only a small amount of blood in heart chambers. Moderate amounts of ingesta in the proventriculus containing a lot of oil (no plastic). Only a small amount of green ingesta with several small squid beaks were present in the ventriculus along with a large number of plastics. (Most of what was in the ventriculus was plastic.) Some gas with low amounts of ingesta present in the intestines. Suspected partial obstruction of the gastrointestinal tract due to plastic in the ventriculus.

**ST-Wol-40**

| **Collection Year** | | | 2021 | | | | | | **Bird ID** | | | | ST-Wol-40 | | | | |
| --- | --- | --- | --- | --- | --- | --- | --- | --- | --- | --- | --- | --- | --- | --- | --- | --- | --- |
| **Freshness** | | | FFF (Very fresh) | | | | | | **Completeness** | | | | CC | | | | |
| **Body Condition** | Intestinal fat (0-3) | | | | 2 | Subcutaneous fat (0-3) | | | | 2 | Breast muscle (0-3) | | | 3 | BCI (0-9) | | **7** |
| **Gastrointestinal plastic?** | | Proventriculus | | | | | Y | Ventriculus | | | N | **Bursa of Fabricius?** | | | | Y | |
| **Complete GIT?** | | Y | | **If no, details:** | | | | | | | | | | | | | |

**Summary of abnormal/significant findings if present:**

**Scavenging:** Not scavenged.

**External:** Good body condition. No external signs of trauma. Medium sized bird. Feathers reasonably mature, with only a few flight feathers retaining a small amount of feather sheath at the base of the shaft.

**Internal:** Lungs are heavy and wet indicating likely water aspiration. Liver and kidneys large but otherwise appear normal in colour and shape. Moderate-large amounts of green ingesta in the proventriculus containing oil droplets and feather fragments. Two pieces of plastic, one positioned close to the isthmus (not obviously causing an obstruction in its current position during necropsy, however little food is present in the ventriculus/upper intestines), and a second piece extending up into the mid proventriculus area where it appeared to be associated with a haemorrhagic area of mucosa with shallow ulcerations 2cm x 0.5cm in size. Not perforated. Ventriculus was almost empty and contained a tiny amount of ingesta with one squid beak and a small amount of sand. Little ingesta in the proximal intestines, moderate amounts of food in the distal section to colon.

**ST-Wol-41**

| **Collection Year** | | | 2021 | | | | | | **Bird ID** | | | | ST-Wol-41 | | | | |
| --- | --- | --- | --- | --- | --- | --- | --- | --- | --- | --- | --- | --- | --- | --- | --- | --- | --- |
| **Freshness** | | | FFF (Very fresh) | | | | | | **Completeness** | | | | I | | | | |
| **Body Condition** | Intestinal fat (0-3) | | | | 2 | Subcutaneous fat (0-3) | | | | 2 | Breast muscle (0-3) | | | 2 | BCI (0-9) | | **6** |
| **Gastrointestinal plastic?** | | Proventriculus | | | | | N | Ventriculus | | | Y | **Bursa of Fabricius?** | | | | Y | |
| **Complete GIT?** | | Y | | **If no, details:** | | | | | | | | | | | | | |

**Summary of abnormal/significant findings if present:**

**Scavenging:** Much of the subcutaneous fat and pectoral muscle was removed from the right side of the chest with a hole into the coelomic cavity. Heart removed; otherwise internal organs were intact.

**External:** High moderate body condition. Medium sized bird. No obvious bleeding.

**Internal:** Lungs very wet, indicating likely aspiration of water. Proventriculus only contained a small amount of ingesta mostly made up by two squid beaks (one quite large.) Ventriculus contained many plastic pieces and little other ingesta. Little ingesta in intestines.

**ST-Wol-42**

| **Collection Year** | | | 2021 | | | | | | **Bird ID** | | | | ST-Wol-42 | | | | |
| --- | --- | --- | --- | --- | --- | --- | --- | --- | --- | --- | --- | --- | --- | --- | --- | --- | --- |
| **Freshness** | | | FFF (Very fresh) | | | | | | **Completeness** | | | | C | | | | |
| **Body Condition** | Intestinal fat (0-3) | | | | 3 | Subcutaneous fat (0-3) | | | | 3 | Breast muscle (0-3) | | | 3 | BCI (0-9) | | **9** |
| **Gastrointestinal plastic?** | | Proventriculus | | | | | N | Ventriculus | | | Y | **Bursa of Fabricius?** | | | | Y | |
| **Complete GIT?** | | Y | | **If no, details:** | | | | | | | | | | | | | |

**Summary of abnormal/significant findings if present:**

**Scavenging:** Moderate scavenging of pectoral muscle on the upper right chest area. Hole into the body cavity with minor damage to right lung.

**External:** Very fat body condition. Large, well grown bird with reasonably mature flight feathers. No obvious bleeding.

**Internal:** Fluid present in lungs and air sacs indicating likely aspiration of water. Empty proventriculus. Plastic in the ventriculus with little other ingesta. Only small amounts of ingesta in the intestines.

**ST-Wol-43**

| **Collection Year** | | | 2021 | | | | | | **Bird ID** | | | | ST-Wol-43 | | | | |
| --- | --- | --- | --- | --- | --- | --- | --- | --- | --- | --- | --- | --- | --- | --- | --- | --- | --- |
| **Freshness** | | | FFF (Very fresh) | | | | | | **Completeness** | | | | CC | | | | |
| **Body Condition** | Intestinal fat (0-3) | | | | 3 | Subcutaneous fat (0-3) | | | | 3 | Breast muscle (0-3) | | | 3 | BCI (0-9) | | **9** |
| **Gastrointestinal plastic?** | | Proventriculus | | | | | Y | Ventriculus | | | Y | **Bursa of Fabricius?** | | | | Y | |
| **Complete GIT?** | | Y | | **If no, details:** | | | | | | | | | | | | | |

**Summary of abnormal/significant findings if present:**

**Scavenging:** Not scavenged.

**External:** Very fat body condition. Large, well grown bird with reasonably mature flight feathers. No sign of external trauma.

**Internal:** Lots of water in air sacs and heavy wet lungs indicating likely aspiration of water. Small amount of ingesta in the proventriculus with multiple pieces of plastic, some extending into the isthmus area. Ventriculus contained multiple pieces of plastic with otherwise only a small amount of ingesta. Many squid beaks and cuttlefish fragments were present in both the proventriculus and ventriculus. Low amounts of ingesta in intestines.
